# ELMO2 association with G*α*i2 regulates pancreatic cancer cell chemotaxis and metastasis

**DOI:** 10.7717/peerj.8910

**Published:** 2020-04-06

**Authors:** Yecheng Wang, Hongyan Li, Fei Li

**Affiliations:** Department of General Surgery, Xuanwu Hospital, Capital Medical University, Beijing, China

**Keywords:** Pancreatic cancer, ELMO2, Gαi2, Metastasis, Cell migration, Chemotaxis

## Abstract

**Background:**

Pancreatic cancer is a highly lethal disease. Nearly half of the patients have distant metastasis and remain asymptomatic. Emerging evidence suggests that the chemokine, CXCL12, has a role in cancer metastasis. The interaction between CXCL12 and CXCR4 activates heterotrimeric G proteins, which regulates actin polymerization and cancer cell migration. However, the molecular mechanisms underlying pancreatic cancer cell migration are still largely obscure. Here, we addressed the role of ELMO2 in chemotaxis and metastasis of pancreatic cancer cells.

**Methods:**

Pancreatic cancer cell lines PANC-1 and AsPC-1 and siRNA-mediated knockdown of ELMO2 were used to determine the effects of ELMO2 on cancer cell chemotaxis, invasion, migration. Co-immunoprecipitation assays were carried out to identify interacting partners of ELMO2.

**Results:**

ELMO2 knockdown inhibited pancreatic cancer cell chemotaxis, migration, invasion, and F-actin polymerization. Co-immunoprecipitation assays revealed that ELMO2 interacted with G*α*i2 and that CXCL12 triggered G*α* i2-dependent membrane translocation of ELMO2. Thus, ELMO2 is a potential therapeutic target for pancreatic cancer.

## Introduction

Pancreatic cancer is one of the most malignant cancers of the digestive system. Currently, it is the fourth leading cause of cancer-related death due to early invasion and rapid metastasis (Kleeff et al., 2016; Siegel, Miller & Jemal, 2018). Because of the lack of reliable markers for early diagnosis and aggressive tumor biology, the 5-year overall survival rate is still extremely low, and, despite important clinical advancements, the outcome is unfavorable in most patients (Conroy et al., 2016; Garrido-Laguna & Hidalgo, 2015). Therefore, it is of vital importance to discover and characterize the molecular mechanisms underlying pancreatic cancer cell migration and metastasis. This may help identify novel factors involved in multi-step tumor metastasis and improve the treatments.

ELMO (Engulfment and Cell Motility) is a family of related scaffold proteins involved in intracellular signaling networks and with a high degree of evolutionary conservation. In mammals, the ELMO protein family consists of three isoforms: ELMO1, ELMO2, and ELMO3. As the mammalian homologs of *Caenorhabditis elegans* CED-12, the ELMO proteins play a major role in cell migration and cytoskeletal rearrangements (Gumienny et al., 2001). Although they lack intrinsic catalytic activity, ELMO proteins can function as adaptors to regulate the activity of plasma membrane and cytoplasmic proteins (Patel, Pelletier & Cote, 2011). Previous studies have shown that ELMO protein interactions with a number of different proteins activate signaling pathways that cause cell migration or promote cell movement. Proteins interacting with ELMO, such as Gαi2, G β *γ*, and Nck-1, are mostly cell membrane-associated and involved in the regulation of cytoskeletal organization (Fritsch et al., 2013; Li et al., 2013; Zhang et al., 2014). Interestingly, ELMO family members such as ELMO1 and ELMO3 have been implicated in a variety of malignant cancers, such as glioma, breast cancer, colorectal cancer, hepatocellular carcinoma, and non-small-cell lung carcinoma (Fan et al., 2015; Jarzynka et al., 2007; Jiang et al., 2011; Peng et al., 2016; Zhang et al., 2015). In previous studies, the researches mostly focused on the function of ELMO1 and EMLO3 in tumor development, invasion, and formation of metastasis. However, the studies about the role and function of ELMO2 were very few. Early reports only showed that ELMO2 localized to cell–cell contacts regulating both integrin- and cadherin-based adhesions, which facilitated to reposition the cells from migration to strong cell–cell adhesion (Toret, Collins & Nelson, 2014). Unfortunately, the biological behaviors and molecular mechanisms of ELMO2 remained unclear. To our knowledge no previous studies have illustrated a relationship between ELMO2 and invasiveness in pancreatic cancer. This study aimed to fully understand the function and mechanisms of ELMO2 in pancreatic cancer chemotaxis and metastasis.

G proteins, also known as guanine nucleotide-binding proteins, are a family of molecular transducers involved in the transmission of signals generated by a variety of stimuli, such as chemokines, neurotransmitters, and hormones. G proteins are typically represented by the membrane-associated heterotrimeric G proteins, which are activated by G protein-coupled receptors (GPCRs), and are engaged in cell signaling. Heterotrimeric G proteins consist of three major subunits, alpha (*α*), beta (β), and gamma (γ). During the past two decades, the function of G proteins has been extensively investigated. Signaling molecules like chemokines bind to the extracellular GPCR domain, after which an intracellular domain facilitates the dissociation of the heterotrimeric G proteins, Gαi and G βγ, which in turn activates a cascade of intracellular signaling events (Fraser, 2008; Xu et al., 2010). GPCRs and G proteins may cooperate in the regulation of cell actin cytoskeleton. The accumulation of actin filaments at leading-edge protrusions of the cell membrane increases cell mobility and promotes cell migration (Hurst & Hooks, 2009; Kim et al., 2013; Muller et al., 2001). However, little information is available on the role of G proteins in the migration and metastasis of pancreatic cancer cells.

In metastasis, cancer cells detach from the primary tumor, travel through the bloodstream or lymph system, and form a new tumor in other organs or tissues. To metastasize or spread, cancer cells need to invade, escape from a proper vessel, and settle at a distant site (Condeelis & Segall, 2003). Chemotaxis is the movement of an organism or cell in response to a chemical stimulus (Iglesias & Devreotes, 2008). Chemokines are a family of small cytokines, signaling proteins secreted by a variety of cells. They induce chemotaxis by interacting with specific chemokine receptors on the surfaces of target cells. The crucial role of chemotaxis in the recruitment of inflammatory cells to infection sites is a long-established concept (Jin et al., 2009). Interestingly, recent studies have shown that chemotaxis is critical for cancer cell dissemination (Condeelis, Singer & Segall, 2005; Murphy, 2001). A complex network of chemokines contributes to chemotaxis in tumor cells, regulating cancer cell growth, invasion, and metastatic progression (Balkwill, 2004; Swaney, Huang & Devreotes, 2010). A particularly important role in chemotaxis is played by the chemokine receptor, CXCR4, and by its ligand, CXCL12 (also known as SDF1, stromal cell-derived factor (1), which initiate directed cell migration in various kinds of cancer (Archibald et al., 2012; Hu et al., 2014; Li et al., 2014; Yang et al., 2015). The CXCR4/CXCL12 interaction triggers downstream signaling cascades that may promote metastatic progression (Guyon, 2014). However, the molecular mechanism by which this complex affects metastasis in pancreatic cancer remains to be elucidated.

In this study, we investigated the role of ELMO2 and CXCL12 in pancreatic cancer using cancer cell lines. The results of this study are expected to provide novel insights into the metastatic progression of pancreatic cancer cells.

## Materials & Methods

### Cell culture

The pancreatic cancer cell lines, PANC-1 and AsPC-1, were purchased from American Type Culture Collection (Manassas, VA, USA). All pancreatic cancer cell lines were cultured in RPMI-1640 medium (Hyclone, Shanghai, China) supplemented with 10% fetal bovine serum (FBS; Gibco Invitrogen Corporation, Australia) and were incubated at 37 ° C in a humidified atmosphere containing 5% CO_2_.

### Western blotting

After indicated treatments, cells were collected and total proteins were extracted with PMSF (Beyotime Biotechnology, shanghai, China) containing RIPA lysis buffer (Beyotime Biotechnology, shanghai, China) as instructed. Protein samples were quantified by Pierce™ BCA Protein Assay Kit (Thermo Scientific) via spectrophotometer (Thermo Scientific, USA) at 562 nm. Protein loading buffer (Sigma) was applied to denature protein samples. Total protein samples (20 µg/lane) were separated by 10% sodium dodecyl sulfate polyacrylamide gel electrophoresis (SDS-PAGE), and transferred to a polyvinylidene difluoride membrane (GE Healthcare Amersham™ Hybond™). The membranes were blocked with 5% skim milk for one hour at room temperature and incubated with the primary antibodies anti-ELMO2 (ab2240, Abcam), anti-Gαi2 (sc-13534, Santa Cruz Biotechnology) or anti-GAPDH (ab8245, Abcam) at 4 °C overnight, and then were washed three times with PBS containing 0.1% Tween-20 (PBST). The membranes were then incubated with the horseradish peroxidase-conjugated secondary antibodies anti-goat (ab6885, Abcam), anti-mouse (sc-516102, Santa Cruz Biotechnology) or anti-rabbit (ab6721, Abcam) for 1 h at room temperature. Finally, the bound proteins were visualized using the SuperSignal™ West Pico PLUS Chemiluminescent Substrate (Thermo Scientific) and analyzed with FluorChemHD2 system (ProteinSimple, USA).

### Transient transfection

Pancreatic cancer cell lines were cultured until they reached 60–80% of confluence before transfection. To reduce the expression of ELMO2 and Gαi2, specific siRNAs were used for *in vitro* transfection. Cells were then incubated for 48 h, followed by protein expression analysis by western blotting. The sequences of ELMO2 siRNA were 5′-CCCAGAGUAUUAUACCCUCCGUUAU-3′, 5′-CCCACUACAGUGAGAUGCUGGCAUU-3′, and 5′-CACAUCAAUCCAGCCAUGGA- CUUUA-3′. The sequences of G *α*i2 siRNA were 5′-GAGGACCUGAAUAAGCGCAAAGACA-3′, 5′-ACGCCGUCACCGAUGUCAUCAA-3′, and 5′-CCGACACCAAGAACGUGCAG- UUCGU-3′. To enhance the expression of ELMO2 and Gαi2, the overexpression plasmids GV362 and GV141, respectively, were transfected into PANC-1 cells. Both plasmids were purchased from Genechem Co., Ltd (Shanghai, China). All transfections were performed using Lipofectamine 3,000 reagent (Invitrogen) in accordance with the manufacturer’s instructions. For the knockdown of ELMO2 throughout the studies, we chose siRNA_1 whose sequence was 5′-CCCAGAGUAUUAUACCCUCCGUUAU-3′, to perform the following experiments.

### Exogenous Co-immunoprecipitation (Co-IP)

PANC-1 cells were plated in 10-cm culture dishes before transfection. To obtain a high level of exogenous ELMO2 and G *α*i2 expression, PANC-1 cells were transfected with GV362 Flag-ELMO2 and GV141 Flag-Gαi2, respectively. Cells were collected and total proteins were extracted with PMSF (Beyotime Biotechnology, shanghai, China) containing RIPA lysis buffer (Beyotime Biotechnology, shanghai, China). First, we incubated the cell lysates with monoclonal anti-flag antibodies (cat. F3165, Sigma) with continuous mixing overnight. Cell lysates were also immunoprecipitated with control rabbit IgG antibodies (CST-2729, Cell Signaling Technology). Next, PureProteomeTM protein A/G mix magnetic beads (Merck-Millipore) were added to the antibody-antigen complex and subjected to continuous mixing. Third, the precipitates were eluted from the magnetic beads by boiling in electrophoresis sample buffer, separated by SDS-PAGE, and detected with anti-Gαi2 (sc-13534, Santa Cruz Biotechnology) and anti-ELMO2 (ab2240, Abcam) antibodies.

### Immunofluorescence

Briefly, PANC-1 cells were plated in 24-well plates containing round glass coverslips (one per well) and incubated for 24 h to obtain stable attachment to the glass coverslips. Before stimulation with CXCL12 (R&D Systems, Inc.), cells were serum-starved for 3 h, followed by incubation with CXCL12 (100 ng/ml) at 37 °C for 1 h. A solution containing 4% paraformaldehyde was used for cell fixation. Cell membranes were permeabilized with 0.1% Triton X-100. Donkey serum was used for blocking non-specific interactions, based on the species in which the secondary antibody was raised. After the blocking step, cells were incubated with diluted primary antibodies anti-ELMO2 (ab2240, Abcam) or anti-Gαi2 (sc-13534, Santa Cruz Biotechnology) overnight.

The cells were subsequently stained with Alexa Fluor 488-conjugated secondary antibody (cat. A32814, Invitrogen) or 546-conjugated secondary antibody (cat. A10036, Invitrogen) for 1 h at room temperature. Finally, cells on coverslips were mounted and visualized using a Leica TCS SP5 II confocal microscope (Leica Microsystems CMS GmbH).

### Wound-healing assay

Pancreatic cancer cell lines were seeded in 6-well plates and divided into three groups: normal, control, and siELMO2. Cells were cultured until they reached an 80–90% density (ca. 24 h). The cell monolayer was gently and slowly scratched with a 10-µl pipette tip across the well. Then, the wells were gently washed twice with PBS to remove the detached cells. The medium was replaced by RPMI 1640 medium with 1% FBS. Finally, photographs of the monolayer were taken with a microscope at various time points (0, 3, 6, 9, 12, and 24 h).

### Chemotaxis assay

The Chemotaxis assays were performed as described by the manufacturer (Neuro Probe) and Sun et al. (2005). In this assay, a 48-well microchemotaxis chamber (AP48 chemotaxis chamber, Neuro Probe) was used, and prepared 50 µl cell suspension (2 ×10^5^ cells per ml) were placed on the upper chamber and were allowed to migrate through the permeable filter membrane (PFB8, Polycarbonate membranes, 25 × 80 mm, Neuro Probe) into the lower chamber. A solution containing the chemokine (0, 10, 100, 1,000 ng/ml CXCL12) was placed below the cell-permeable membrane. After a 3-h incubation in 5% CO2 at 37 °C, the cells that had migrated through the 8-µm filter membrane were fixed, stained, and counted in three separate fields (×400) by a microscope.

### Cell invasion assay

Invasion assays were performed using a transwell chamber inserted with a polycarbonic membrane (cat. 3422 Corning, USA). To reproduce appropriate *in vivo* environments for 2D and 3D cell movements, we added 80 µl of extracellular matrix (Corning 356234) into the upper compartment of the transwell cell culture inserts. CXCL12 (0, 10, 100, 1,000 ng/ml) was added to the lower well of the plates as an attractant. 2 × 10^4^ cells suspended in 100 µl serum-free medium were seeded into the upper chamber. The plates were incubated for 24 h at 37 °C. Then, the cells on the lower side of the insert membrane were fixed. Finally, the cells on the lower side of the filter were counted under a microscope.

### Adhesion assay

Briefly, a fibronectin (Sigma-Aldrich Corporation) solution was previously prepared and stored at 4 ° C. Then, 96-well plates (Costar-3599, Corning, US) were coated with fibronectin (10 *μ*g/ml in PBS) at room temperature for 1 h. After coating, the fibronectin solution was removed. Thermally denatured BSA was added to the plates, followed by an incubation of 1 h at 37 ° C. Then, the plates were washed twice with serum-free RPMI 1,640 medium. CXCL12 (0, 10, 100, and 1,000 ng/ml) was added to the wells of fibronectin-coated plates. After the addition of the prepared 100 µl cell suspension (5 ×10^5^ cells per ml), the plates were incubated at 37 ° C for 30 min and then washed thrice with PBS to remove non-adherent cells. Next, a 10 µl Cell Counting Kit-8 (CCK-8) solution (Dojindo, Japan) was added to each well and incubated for 1.5 h. Finally, the adherent cells were stained and quantified at OD450 using a Microplate Reader (Thermo) according to the manufacturer’s instructions. The cell adhesion ratio of the assay was calculated according to the following formula: cell adhesion (%) = OD of the adhered cells /OD of the total cells ×100%.

### F-actin polymerization assay

Cellular F-actin measurement was done as described by Tsuboi (2006). Pancreatic cancer cells were stimulated with 100 ng/ml CXCL12 for the designated time points at 37 ° C. Then, the cells were fixed with 4% paraformaldehyde, permeabilized with 0.1% Triton X-100, and stained with Alexa Fluor 568-phalloidin (Invitrogen) for 60 min at room temperature. The level of F-actin was measured by a microplate fluorescence reader. The results were expressed as relative F-actin values, as follows:

\begin{eqnarray*}\mathrm{F}-\mathrm{actin} \mathrm{t/ F}-\mathrm{actin} 0=(\mathrm{fluorescence} \mathrm{t/ mg} ml-1)/(\mathrm{fluorescence} t0/\mathrm{mg} \mathrm{ml}-1). \end{eqnarray*}

### Statistical analysis

The statistical analyses were performed with GraphPad Prism 8 (La Jolla, CA, USA). The experimental data are expressed as the mean ± SD. We designed each experiment with 3 replicates, which were done all at the same time. And each experiment was performed three times. One-way and two-way ANOVA were used to analyze the data. A *p* value below 0.05 was considered statistically significant.

## Results

### Role of ELMO2 in the migration and chemotaxis of pancreatic cancer cells

**Figure 1:**
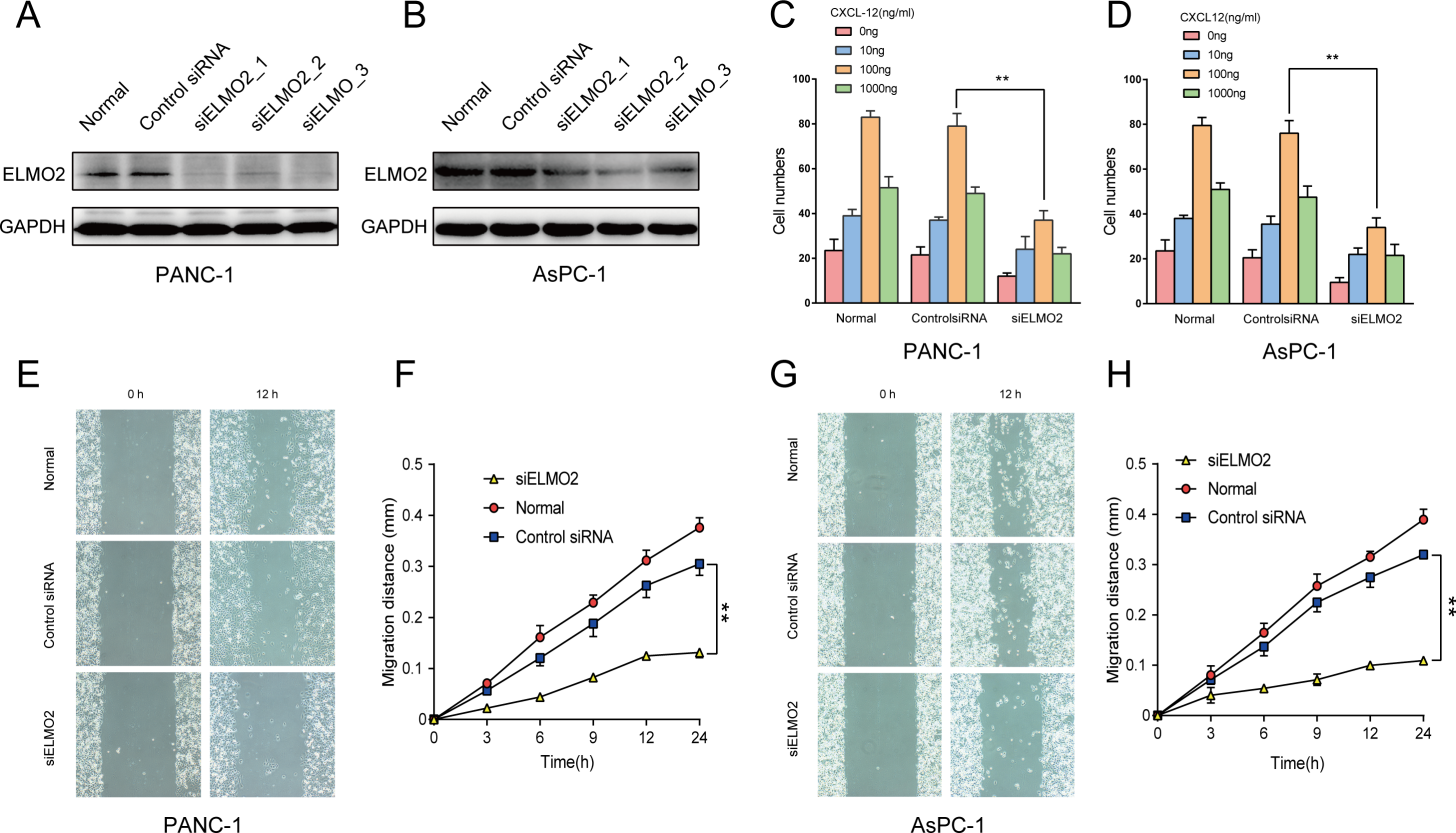
Function of ELMO2 in pancreatic cancer cell migration and chemotaxis. (A, B) Western blot shows an evident knockdown of ELMO2 in human pancreatic cell lines. GAPDH was used as a loading control for western blot. (C, D) Chemotaxis in ELMO2 knockdown cells (data are the mean of three independent experiments; two-way ANOVA, ^∗∗^*p* < 0.001). (E–H) Wound healing assay in siELMO2 cells. Pancreatic cancer cells were seeded in 6-well plates and incubated until the monolayer reached 80–90% confluence (ca. 24 h of growth). The medium was replaced by an RPMI 1640 medium containing 1% fetal bovine serum. The gap distance was measured at 0, 3, 6, 9, 12, and 24 h (data are the mean of three independent experiments; two-way ANOVA, ^∗∗^*p* < 0.001).

To explore the role played by ELMO2 in the process of cell migration, we initially investigated its expression level in pancreatic cancer cell lines. The reasons why PANC-1 and AsPC-1 were chosen in this study were as follows: Firstly, information concerning the clinical course of the sites where cell lines were deprived from was important in defining the biologic and pathologic characteristics of the tumor cell lines. Both these two cell lines were derived from patients with an adenocarcinoma in the head of the pancreas and they shared similar phenotypic characteristics, such as adhesion, invasion and migration. Secondly, the cell population doubling times for PANC-1 and AsPC-1 were very close which made it more convenient for our experimental operation. Small interfering RNA (siRNA) was used to suppress ELMO2 expression (Fig. 1A). Then, a wound-healing assay was utilized to evaluate cell migration. The decreased expression of ELMO2 reduced the migration capacity of PANC-1 and AsPC-1 cells (Fig. 1C). Moreover, a chemotaxis assay indicated that CXCL12 could distinctly enhance the chemotactic ability of PANC-1 and AsPC-1 cells, while ELMO2 silencing inhibited the CXCL12-induced chemotaxis in these cell lines (Fig. 1B).

### Knockdown of ELMO2 inhibited invasion, adhesion, and F-actin polymerization in PANC-1 and AsPC-1 cells

**Figure 2:**
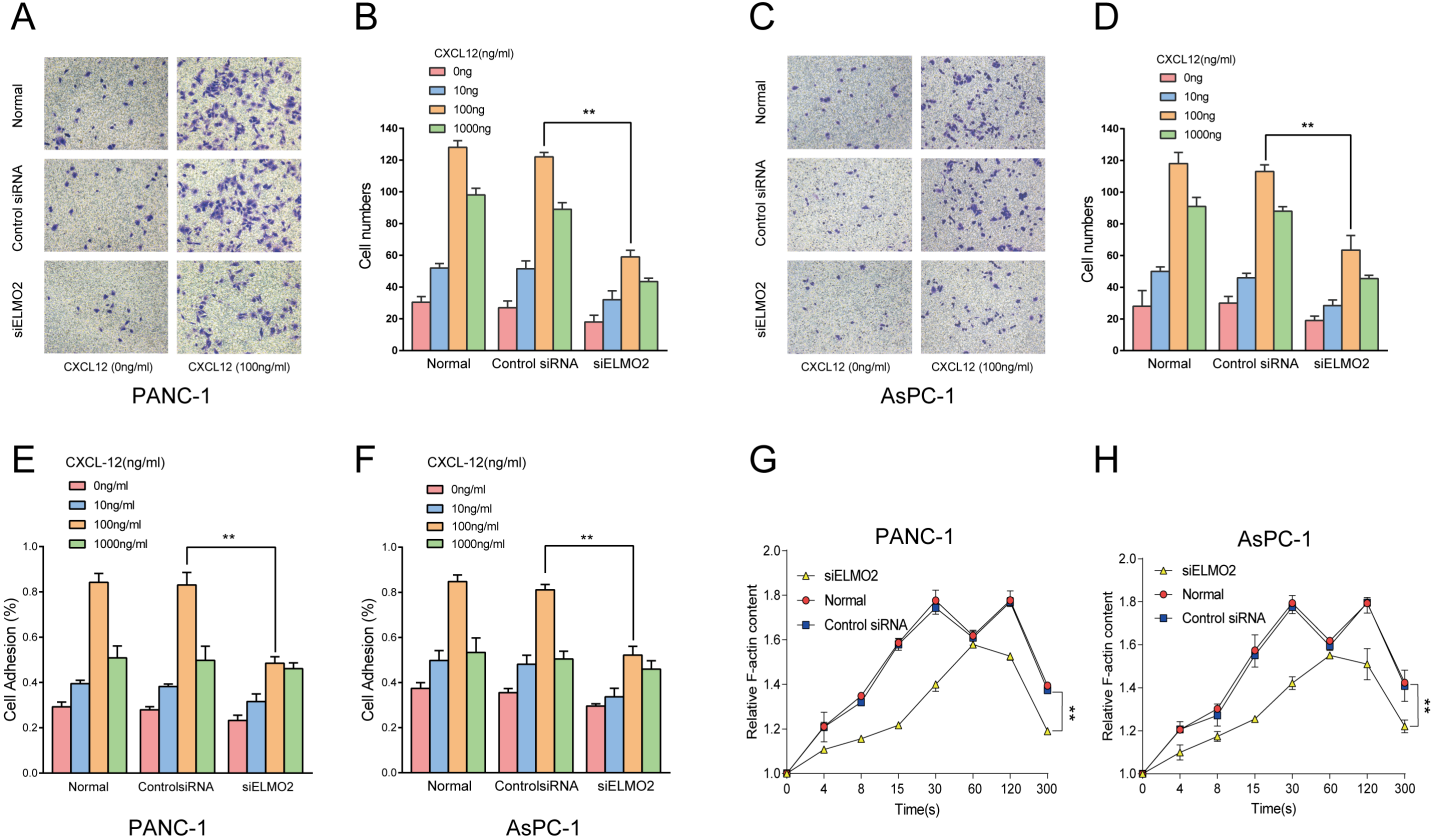
Knockdown of ELMO2 inhibited F-actin polymerization and invasion in pancreatic cancer cells. (A–D) The invasion assay showed that ELMO2 knockdown decreased the CXCL12-mediated invasive abilities of PANC-1 and AsPC-1 cells (data are the mean of three independent experiments; two-way ANOVA, ^∗∗^*p* < 0.001). (E, F) Adhesion assay in PANC-1 and AsPC-1 cells. The cell adhesion rate was much higher in normal cells than in siELMO2 cells (data are the mean of three independent experiments; two-way ANOVA, ^∗∗^*p* < 0.001). (G, H) ELMO2 knockdown reduced actin polymerization in pancreatic cancer cells. F-actin value was measured at different time points (0, 4, 8, 15, 30, 60, 120, and 300 s). Time course of relative F-actin content in normal, control, and siELMO2 cells upon CXCL12 stimulation (data are the mean of three independent experiments; two-way ANOVA, ^∗∗^*p* < 0.001).

Next, a cell invasion assay was performed to monitor the movement of pancreatic cancer cells through an extracellular matrix. We found that ELMO2 knockdown suppressed CXCL12-induced invasiveness in both PANC-1 and AsPC-1 cells (Fig. 2A). Furthermore, cell adhesion assay suggested that ELMO2 downregulation by siRNA weakened the adhesion ability of PANC-1 and AsPC-1 cells (Fig. 2B). When CXCL12 combines with its receptor CXCR4, intracellular signaling events induce membrane protrusions because of actin polymerization. These events enhance the motility of cancer cells and promote chemotaxis and invasion. CXCL12 generated a transient F-actin accumulation in PANC-1 and AsPC-1 cells, in line with previous findings (Yan et al., 2012). Notably, F-actin filaments were clearly reduced in siELMO2 cells within 30 s (Fig. 2C). These results suggested that ELMO2 knockdown inhibited F-actin polymerization in pancreatic cancer cells. Thus, ELMO2 might participate in CXCL12-mediated invasion.

### ELMO2 interacts with G *α*i2

**Figure 3:**
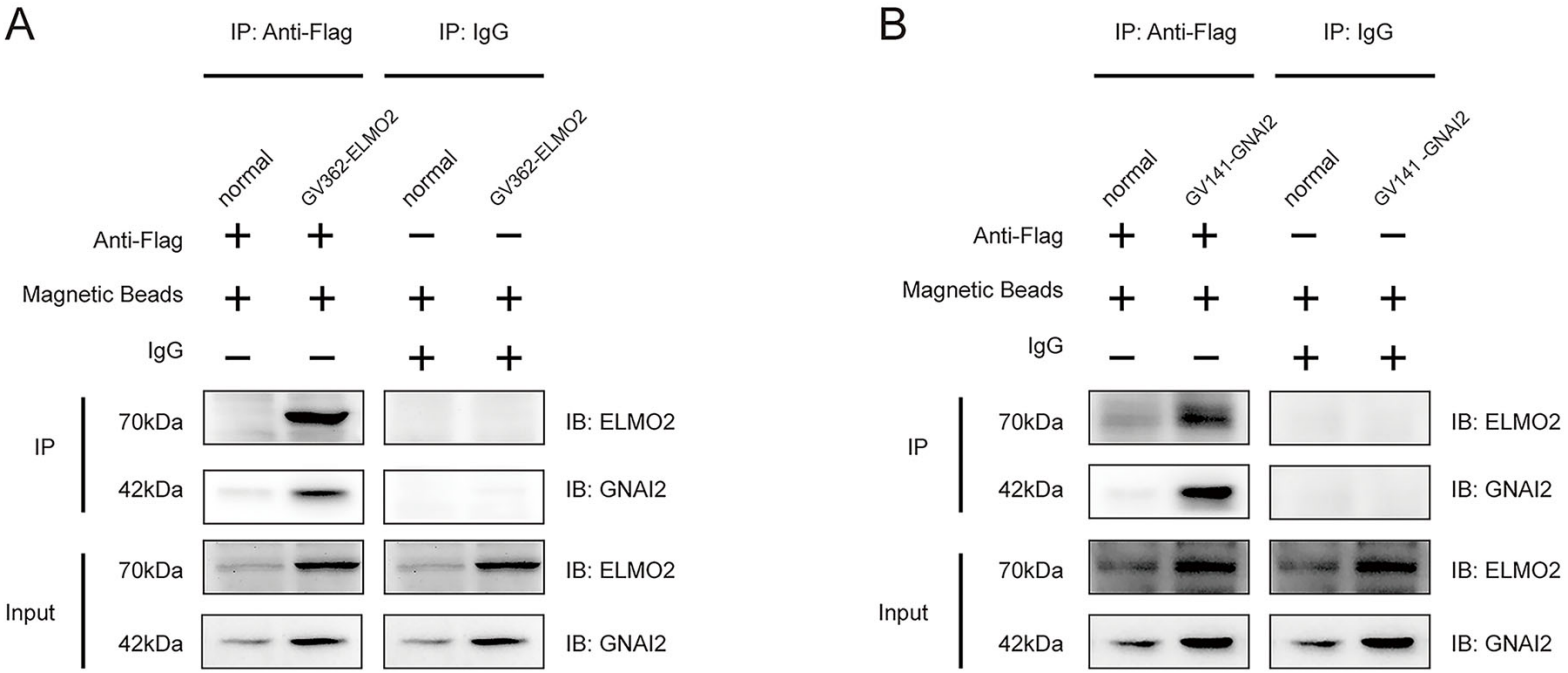
ELMO2 interacts with Gαi2. (A) PANC-1 cells were transfected with GV362 Flag-ELMO2 to increase ELMO2 expression. Cell lysates were immunoprecipitated with anti-Flag antibody, and magnetic beads were used to capture the immune complexes. The eluted proteins were separated by SDS-PAGE and detected with specific antibodies. (B) Exogenous Gαi2 was overexpressed by transfection with the GV141-GNAI2-Flag plasmid. Proteins of the immune complex were pulled down by anti-Flag antibody and detected by anti-ELMO2 or anti-Gαi2 antibodies. Input was the total protein lysates which were prepared from cells with RIPA lysis buffer. IP referred to the immunoprecipitate that was eluted from the beads after the immunoprecipitation.

Previous reports have shown that the association between ELMO1 and Gαi2 contributes to actin polymerization in human breast cancer cells. Thus, it was reasonable to expect that G *α*i2 interacts with ELMO2 in pancreatic cancer cells. To verify this possibility, a Co-IP assay was performed. First, exogenous overexpression of ELMO2 was successfully induced by transfecting PANC-1 cells with the GV362-ELMO2-Flag plasmid. Then, ELMO2-Flag, along with its endogenous interactors, was captured from PANC-1 cell lysates using specific anti-Flag antibody. Interestingly, Gαi2 was found among the ELMO2-interacting partners, as assessed by immunoblotting with G *α*i2 antibody (Fig. 3A). Moreover, when Gαi2-Flag was overexpressed following cell transfection with the GV141-GNAI2-Flag plasmid, endogenous ELMO2 was co-precipitated by the anti-Flag antibody along with Gαi2-Flag (Fig. 3B). Taken together, our results confirmed the physical association between ELMO2 and G *α*i2 in pancreatic cancer cells.

### Cell stimulation with CXCL12 results in ELMO2 membrane translocation

**Figure 4:**
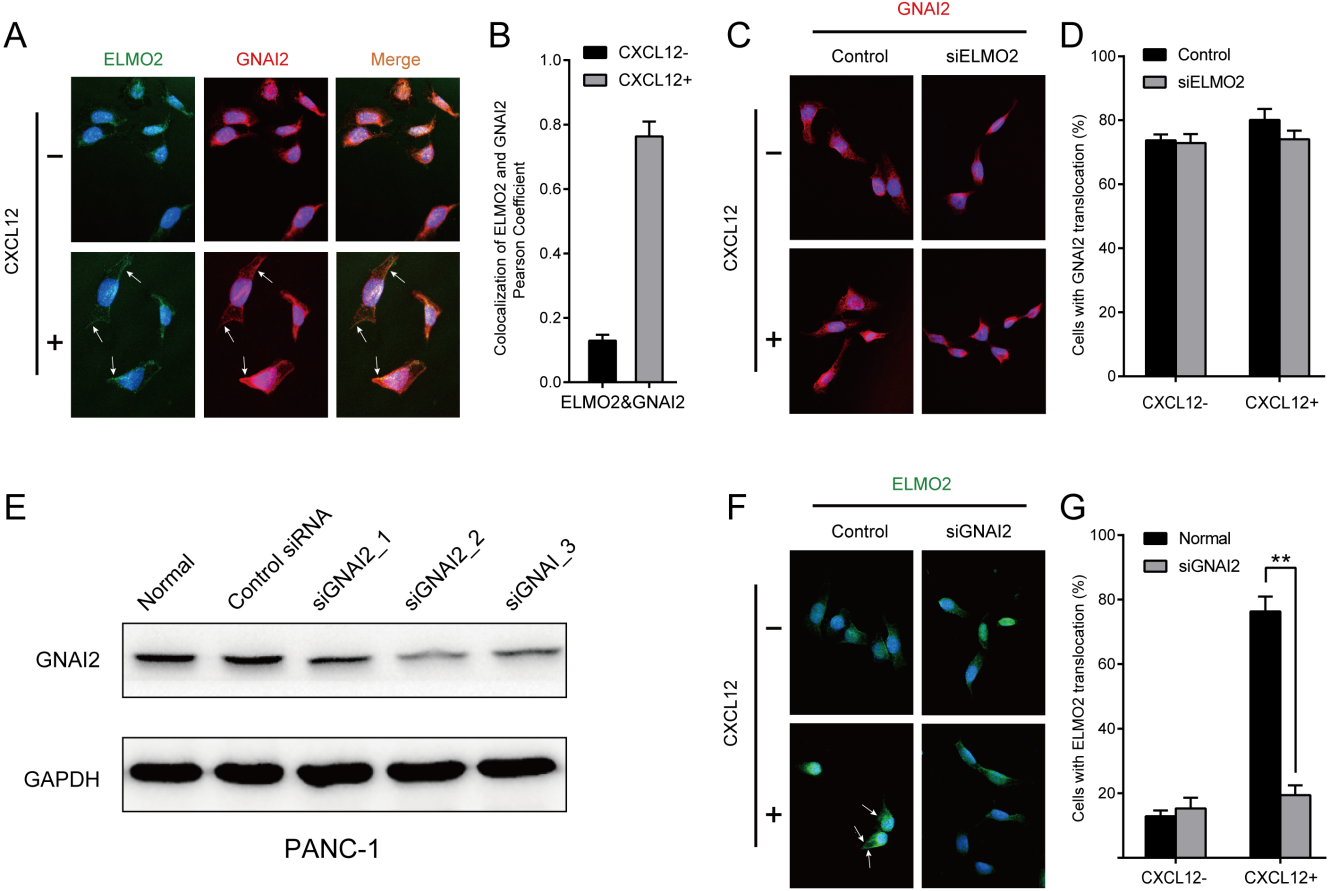
CXCL12 stimulation results in ELMO2 membrane translocation. (A, B) Plasma membrane colocalizations of Gαi2 and ELMO2 were evident upon pancreatic cancer cell stimulation with CXCL12. The extent of colocalization was calculated through ImageJ software. (C, D) No significant changes in plasma membrane-associated Gαi2 fluorescence were detected in ELMO2 knockdown cells, even with CXCL12 stimulation. One-way ANOVA, *p* > 0.05. (E) Western blot clearly shows the Gαi2 knockdown in siRNA-transfected PANC-1 cells. GAPDH was used as a loading control. (F, G) ELMO2 membrane translocation was reduced in Gαi2 knockdown cells, even in the presence of CXCL12 stimulation. Twenty-five images were analyzed by ImageJ software. One-way ANOVA, ^∗∗^*p* < 0.001. The arrows indicated the plasma membrane colocalization of Gαi2 or ELMO2.

To further investigate the interaction networks involving ELMO2 and Gαi2, immunofluorescence microscopy was used to examine the subcellular localization of the two proteins. In unstimulated cells, clear Gαi2-specific fluorescence, but not ELMO2 fluorescence, was detected at the plasma membrane. Interestingly, after CXCL12 stimulation, ELMO2 was also detected on the plasma membrane. According to the colocalization analysis performed on a pixel by pixel basis, Gαi2 and ELMO2 were clearly found to co-localize on the plasma membrane after CXCL12 stimulation of pancreatic cancer cells (Fig. 4A). Next, the impact of Gαi2 silencing on ELMO2 localization, and vice versa was explored in PANC-1 cells transfected with the appropriate siRNAs (Figs. 4B, 4C). Interestingly, the membrane translocation of ELMO2 was reduced in Gαi2-knockdown cells, even in the presence of CXCL12 stimulation, while ELMO2 knockdown had no significant impact on the level of plasma membrane Gαi2 (Figs. 4C, 4D). Thus, Gαi2 might be a key factor for chemokine-induced ELMO2 recruitment to the plasma membrane.

## Discussion

Pancreatic cancer is one of the most malignant cancers, causing high morbidity and mortality. This is due to the intrinsic characteristics of this malignancy, such as rapid tissue invasion and metastasis. To acquire invasive and metastatic capabilities, cancer cells need to undergo multiple cellular changes, including oncogene-triggered signaling cascades. Several proteins are involved in these changes at the genetic and biochemical levels. ELMO family proteins are orthologs of *C. elegans* CED-12. They possess no catalytic activity, but associate with other proteins, serving as upstream activators and regulators of cytoskeletal rearrangements, thus favoring cell motility. Several studies have suggested a role of ELMO proteins in cancer. For instance, ELMO1 was clearly related to the invasive phenotype of glioma cells. In addition, the migratory and invasive abilities of glioma cells increase with the level of ELMO1 expression. Other studies have demonstrated that the overexpression of ELMO1 promotes cell motility and invasion in hepatocellular carcinoma and serous ovarian cancer (Li et al., 2019; Wang et al., 2014). ELMO3 has also been reported to participate in events related to metastasis in several types of cancer, including lung cancer, colorectal cancer, and squamous-cell carcinoma of the head and neck (Fan et al., 2015; Kadletz et al., 2017; Peng et al., 2016). However, the function of ELMO2 in pancreatic cancer progression and metastasis has been poorly investigated. During the chemotaxis assay, a permeable filter separated the upper and lower wells of the Neuro Probe AP48 chemotaxis chamber. Interestingly, cell migration through the permeable filter could be inhibited when ELMO2 was reduced. Based on our data, the decrease in the number of migrated cells under ELMO2 knockdown condition without CXCL12 stimulation was statistically significant. The possible reason for this was that the decreased expression of ELMO2 reduced the migration capacity of tumor cells. In this study, we showed that ELMO2 knockdown inhibits CXCL12-mediated migration, chemotaxis, adhesion, and invasion of pancreatic cancer cells. CXCL12 interaction with its receptor, CXCR4, causes intracellular actin polymerization, which is necessary for pancreatic cancer cell migration and invasion. We found that stimulation with CXCL12 induces a noticeable increase in F-actin in pancreatic cancer cells, which can be prevented by ELMO2 knockdown. Therefore, ELMO2 is a boosting factor for the migration and metastasis of pancreatic cancer cells. Further studies will be needed to exhaustively characterize pathologically relevant ELMO2 interactions with other proteins.

During the past few years, extensive efforts have been made to identify potential interactors of ELMO proteins. It has been reported that brain-specific angiogenesis inhibitor (BAI3), a G protein-coupled receptor binding to ELMO1, regulated myoblast fusion (Hamoud et al., 2014). Moreover, a member of the Nck protein family, Nck-1, interacts with ELMO1 and controls the activity of the Rho family GTPase, Rac1, which is involved in the reorganization of the actin cytoskeleton. Furthermore, the membrane-bound proteins Gαi2 and G βγ have been found to associate with ELMO1. These proteins activate downstream signaling factors that have an impact on the restructuring of the actin cytoskeleton and promote the migration of cancer cells. However, the mechanism by which ELMO2 interaction with its putative partner, Gαi2, affects the process of metastasis has not been investigated in pancreatic cancer. In this study, co-immunoprecipitation experiments demonstrated that exogenous ELMO2 directly interacted with endogenous Gαi2, and vice versa. Interestingly, we found that the expression of GNAI2 had increased in the GV362-ELMO2 condition and when we overexpressed GNAI2 it seemed that also the expression level of ELMO2 increased. Overexpression of ELMO2 or Gαi2 might force tumor cells to generate more endogenous Gαi2 or ELMO2 to interact with in order to work together for directing migration and invasion of cancer. Future studies are needed to understand how these two proteins influence over the expression of one another. The interaction of ELMO2 with Gαi2 was enhanced by CXCL12 stimulation. Moreover, CXCL12 stimulation promoted Gαi2-mediated membrane translocation of ELMO2. Interestingly, when the expression of Gαi2 was suppressed in human pancreatic cancer cell line PANC-1, ELMO2 translocation was substantially reduced, even in the presence of CXCL12 stimulation. It was suggested that Gαi2 plays an indispensable role in ELMO2 translocation to the plasma membrane. Our results confirmed this finding and demonstrated that a physiologically relevant interaction between ELMO2 and Gαi2 promoted actin polymerization in pancreatic cancer cells. We thus hypothesize that CXCL12 binding to the G-protein-coupled receptor, CXCR4, triggered a signal that was transmitted to the cell interior, causing ELMO2 recruitment to the plasma membrane. The latter event was dependent on Gαi2. Thus, intracellular signals generated by the newly assembled CXCL12/ CXCR4 protein complex resulted in actin polymerization and invasive cell migration.

In summary, we showed that ELMO2 plays an essential role in CXCL12-mediated chemotaxis, migration, and invasion of human pancreatic cancer lines. Gαi2 interacted directly with ELMO2 to promote metastatic changes. Considering these results, ELMO2 may be regarded as a promising target for the treatment of pancreatic cancer metastasis. Further research is needed to uncover the role of ELMO-related signaling in different types of cancer, identify valuable prognostic biomarkers, and develop therapeutic strategies centered on ELMO signaling.

##  Supplemental Information

10.7717/peerj.8910/supp-1 Supplemental Information 1 ELMO2 knockdown inhibited pancreatic cancer cell chemotaxis, migration, invasion, cell adhesion and F-actin polymerization

10.7717/peerj.8910/supp-2 Figure S2 Gαi2 was found to be a key factor for chemokine-induced ELMO2 recruitment to the plasma membrane To further investigate interaction networks involving ELMO2 and Gαi2, immunofluorescence microscopy was used to examine the subcellular localization of the two proteins.

10.7717/peerj.8910/supp-3 Figure S3 Co-immunoprecipitation assays revealed that ELMO2 interacted with Gαi2 Our results confirmed the physical association between ELMO2 and Gαi2 in pancreatic cancer cells.

## Additional Information and Declarations

### Data Availability

The following information was supplied regarding data availability: The raw data are available in the Supplementary Files.
