# Supplementary figures and images for: ELMO2 association with Gαi2 regulates pancreatic cancer cell chemotaxis and metastasis (part 1 of 2)
Source: PeerJ. 2020 Apr 6;8:e8910. doi: 10.7717/peerj.8910 (PMC7144586; doi:10.7717/peerj.8910)

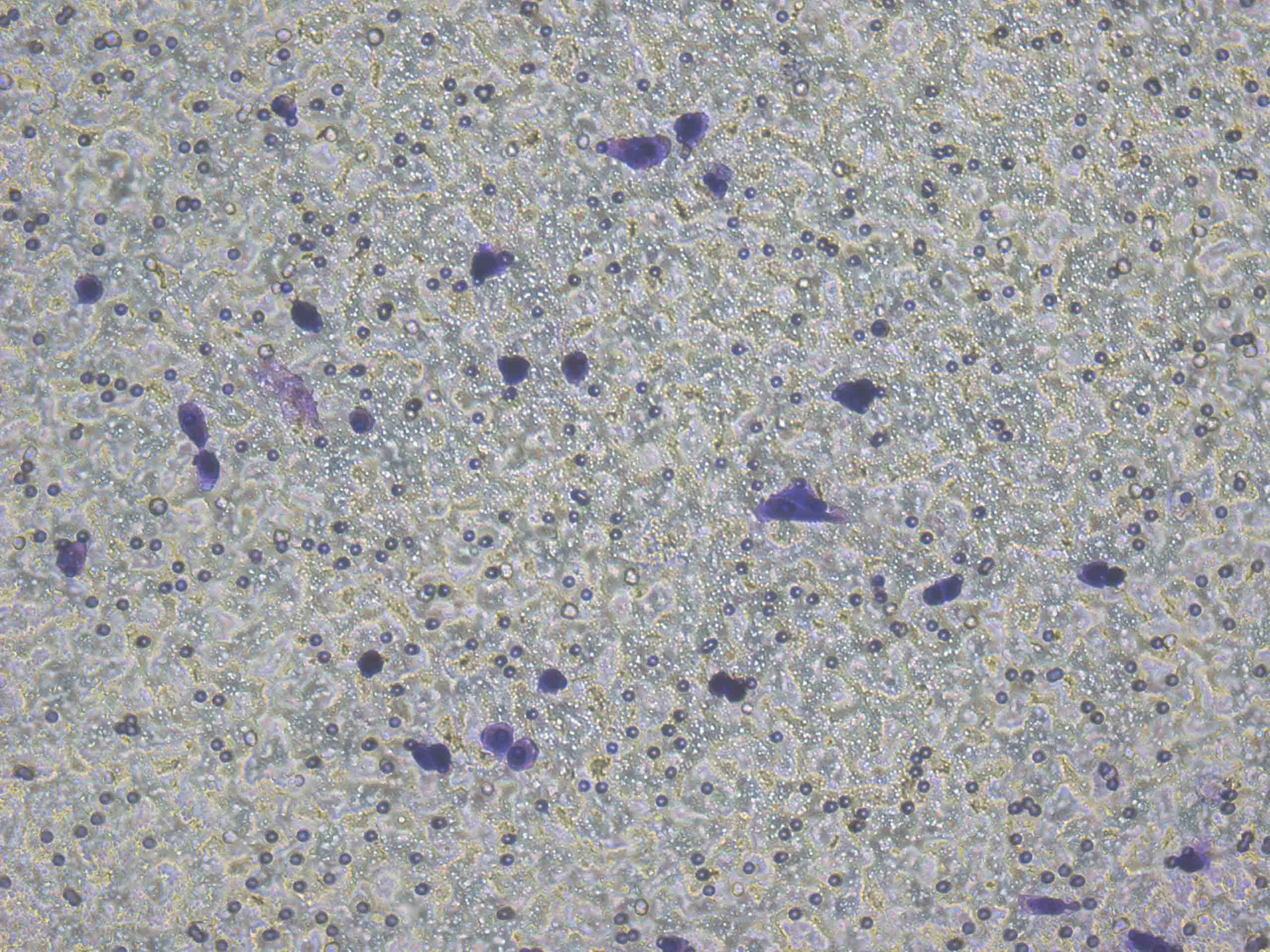

Supplement: Supplemental Information 1 [file peerj-08-8910-s001.zip › invasion_asssy/aspc-1/1/0ng-Control.jpg]

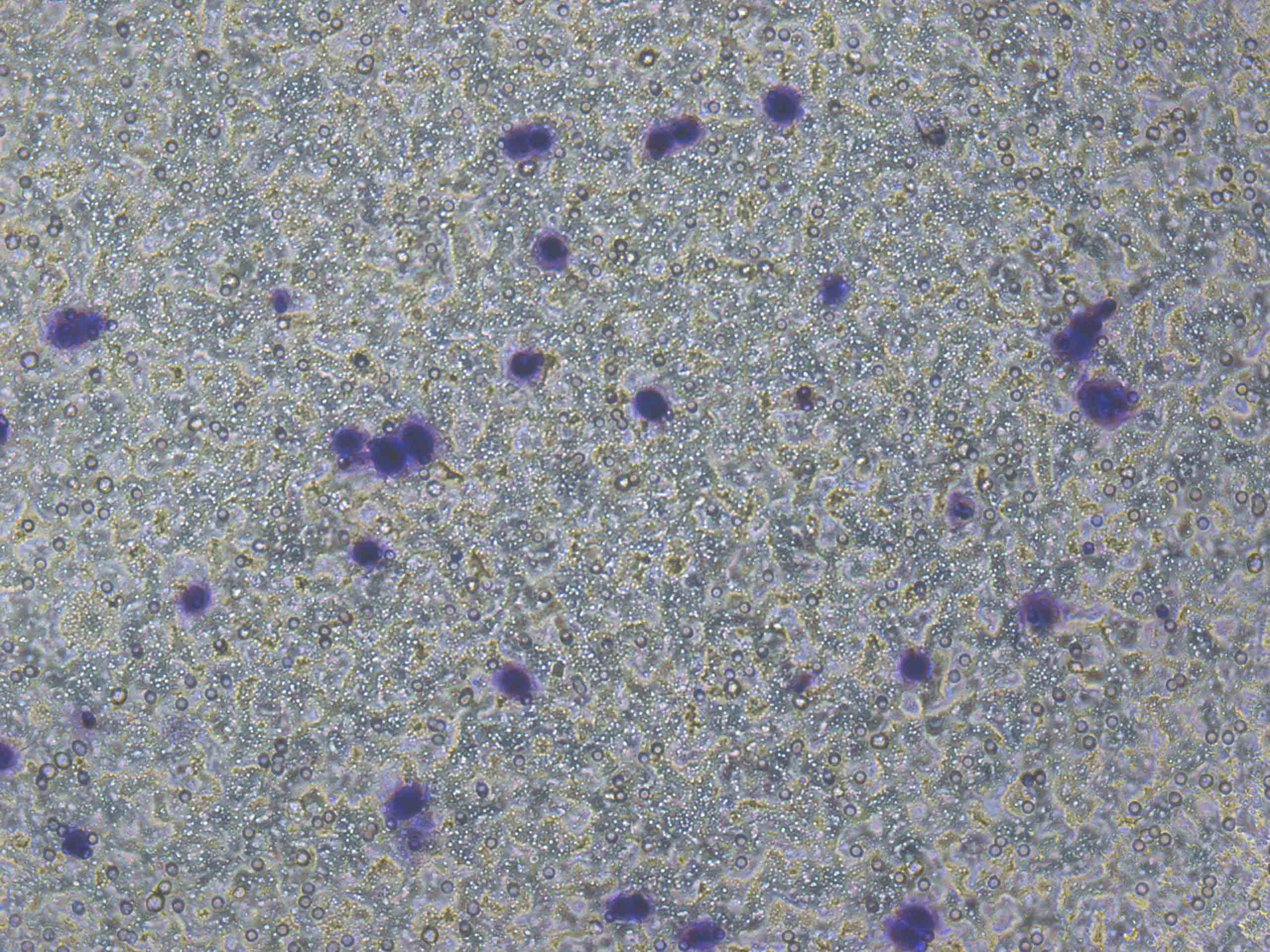

Supplement: Supplemental Information 1 [file peerj-08-8910-s001.zip › invasion_asssy/aspc-1/1/0ng-Normal.jpg]

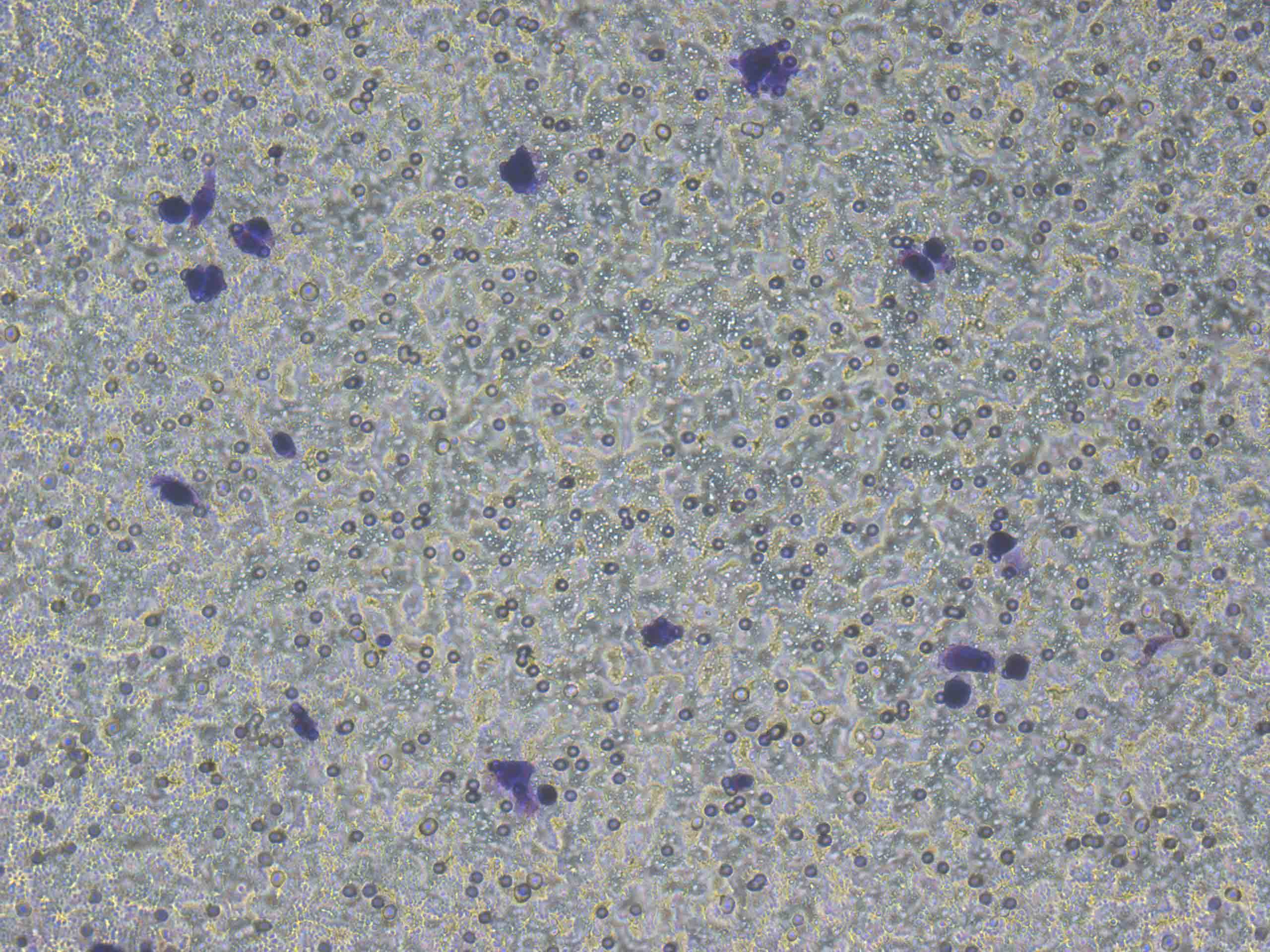

Supplement: Supplemental Information 1 [file peerj-08-8910-s001.zip › invasion_asssy/aspc-1/1/0ng-Si.jpg]

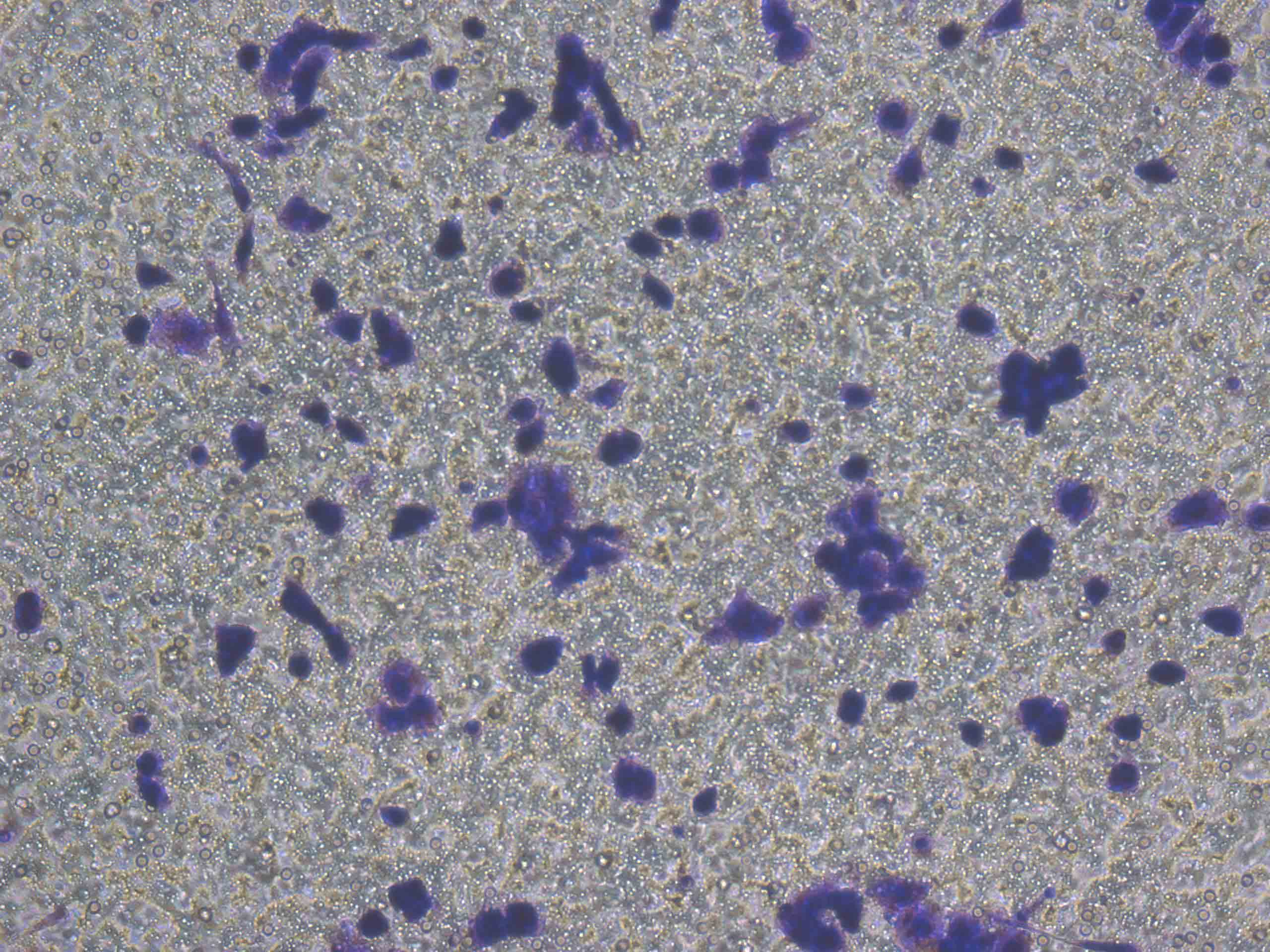

Supplement: Supplemental Information 1 [file peerj-08-8910-s001.zip › invasion_asssy/aspc-1/1/100ng-Control.jpg]

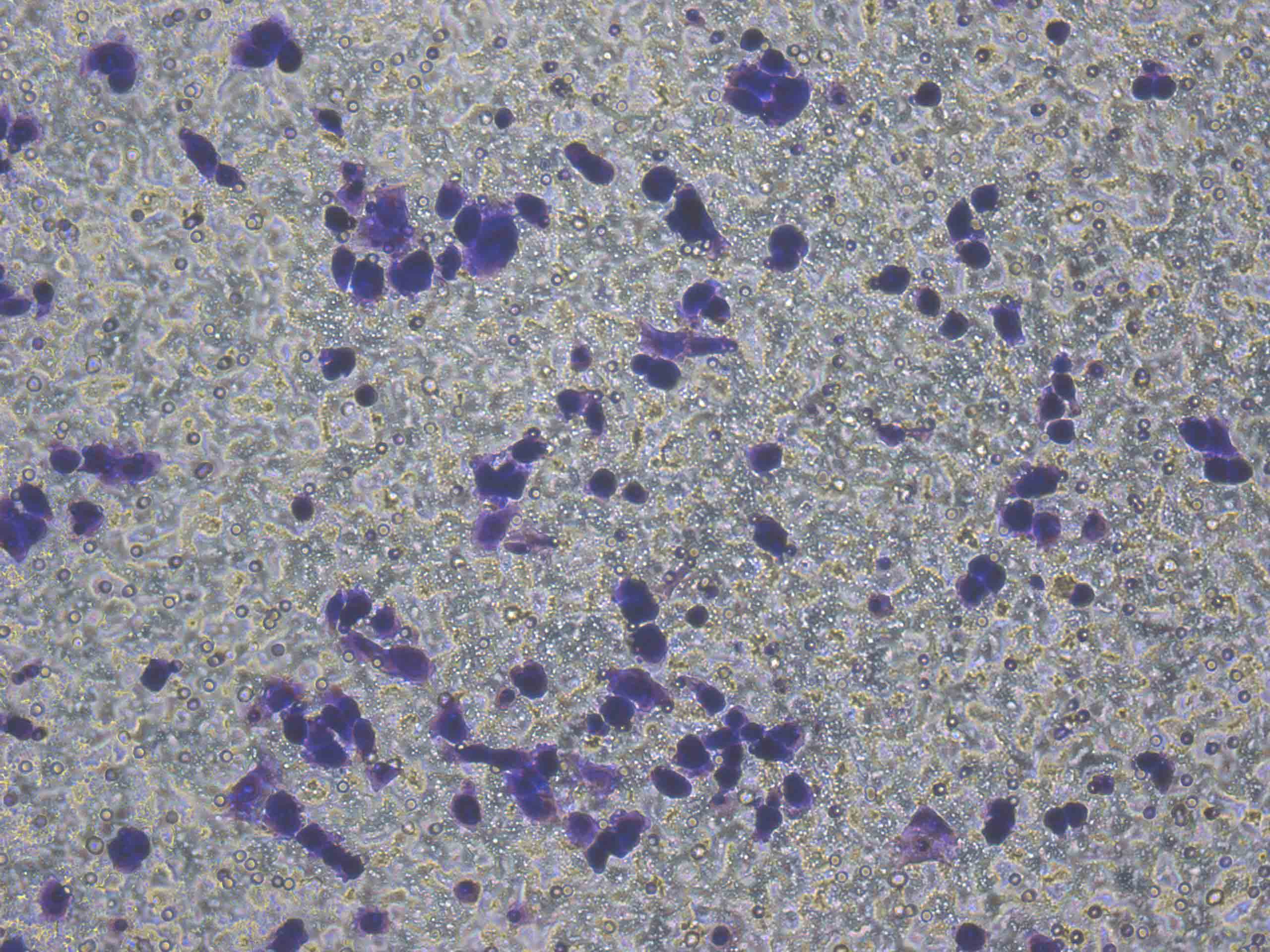

Supplement: Supplemental Information 1 [file peerj-08-8910-s001.zip › invasion_asssy/aspc-1/1/100ng-Normal.jpg]

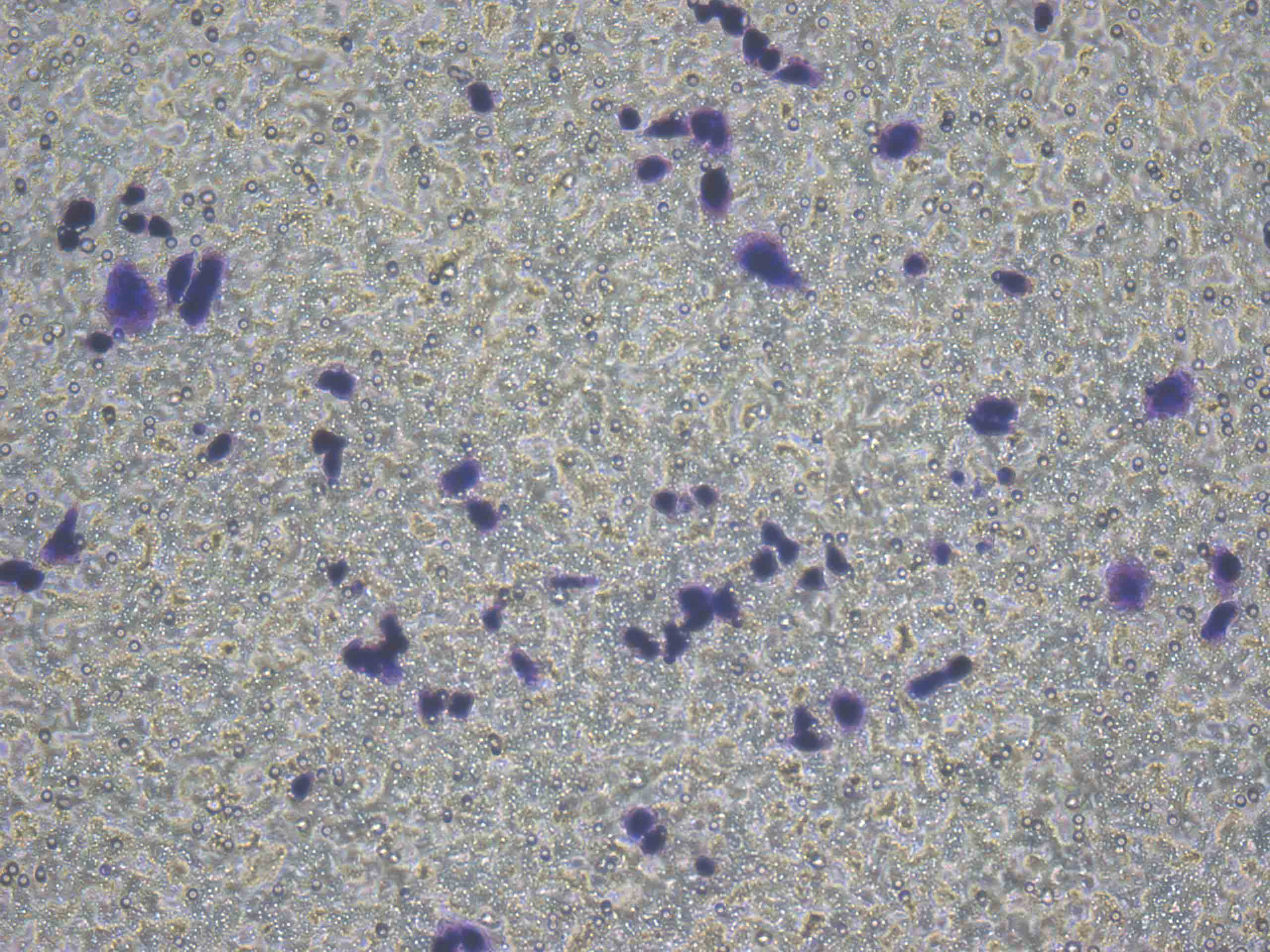

Supplement: Supplemental Information 1 [file peerj-08-8910-s001.zip › invasion_asssy/aspc-1/1/100ng-Si.jpg]

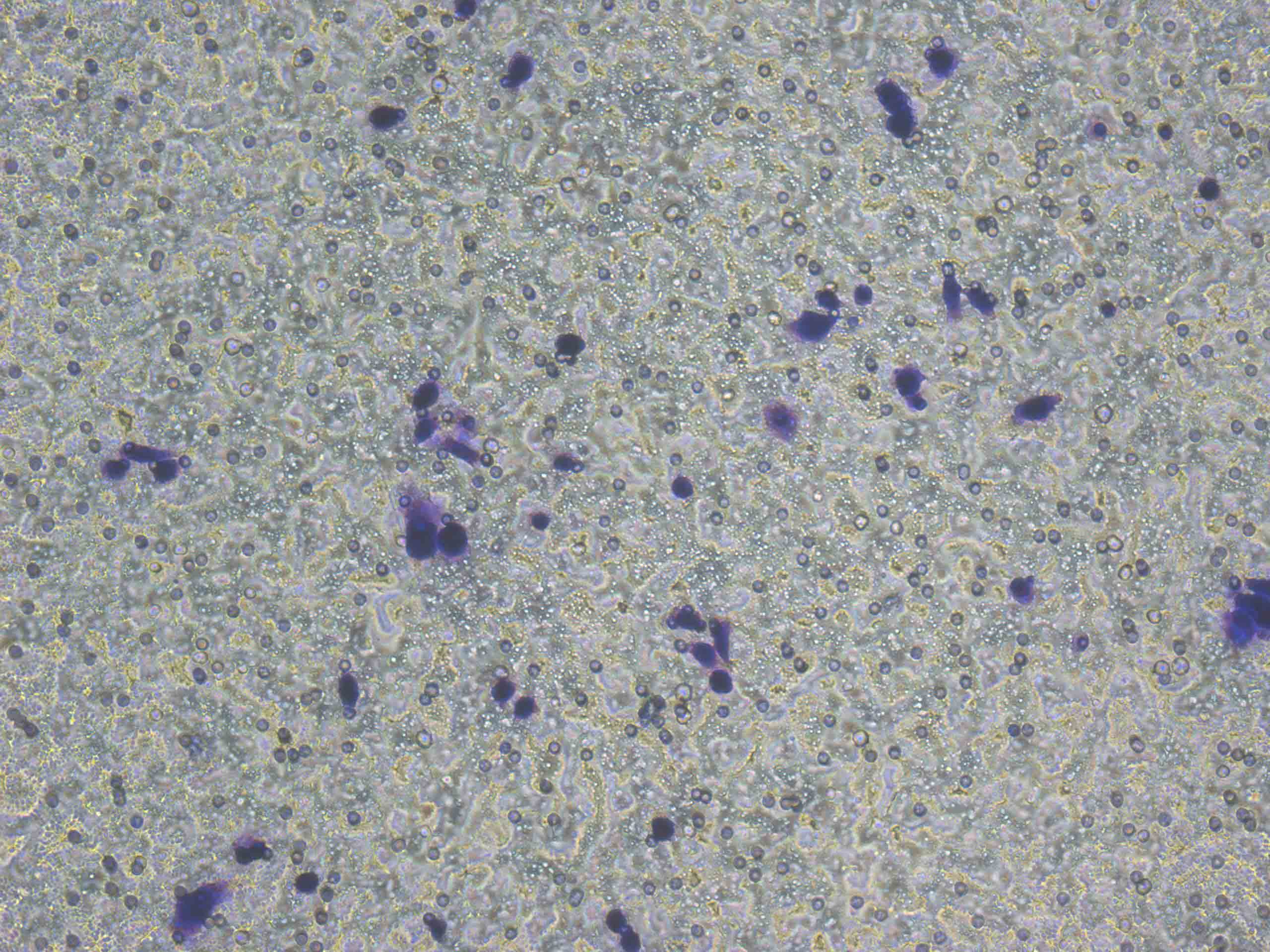

Supplement: Supplemental Information 1 [file peerj-08-8910-s001.zip › invasion_asssy/aspc-1/2/0ng-Control.jpg]

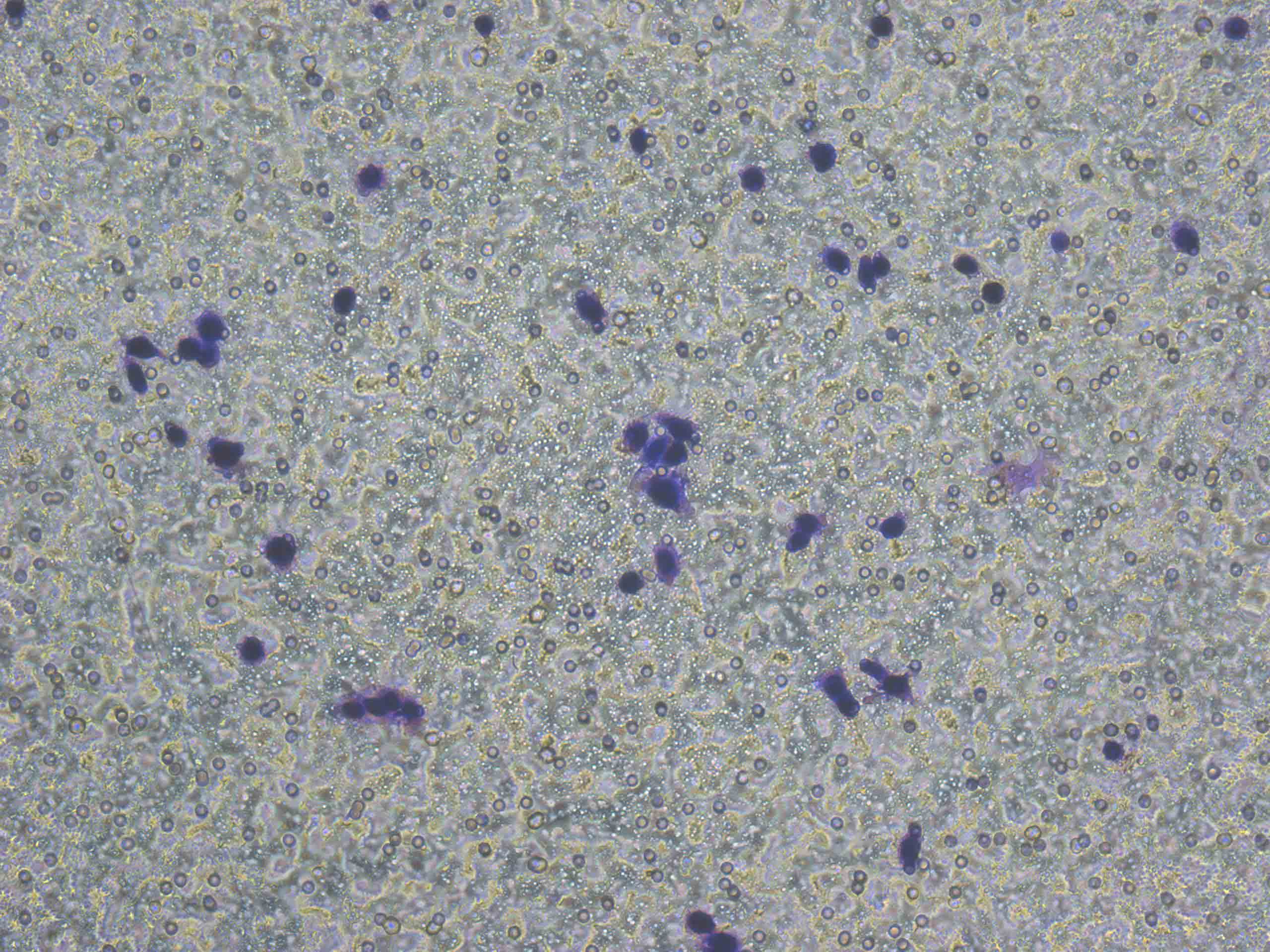

Supplement: Supplemental Information 1 [file peerj-08-8910-s001.zip › invasion_asssy/aspc-1/2/0ng-Normal.jpg]

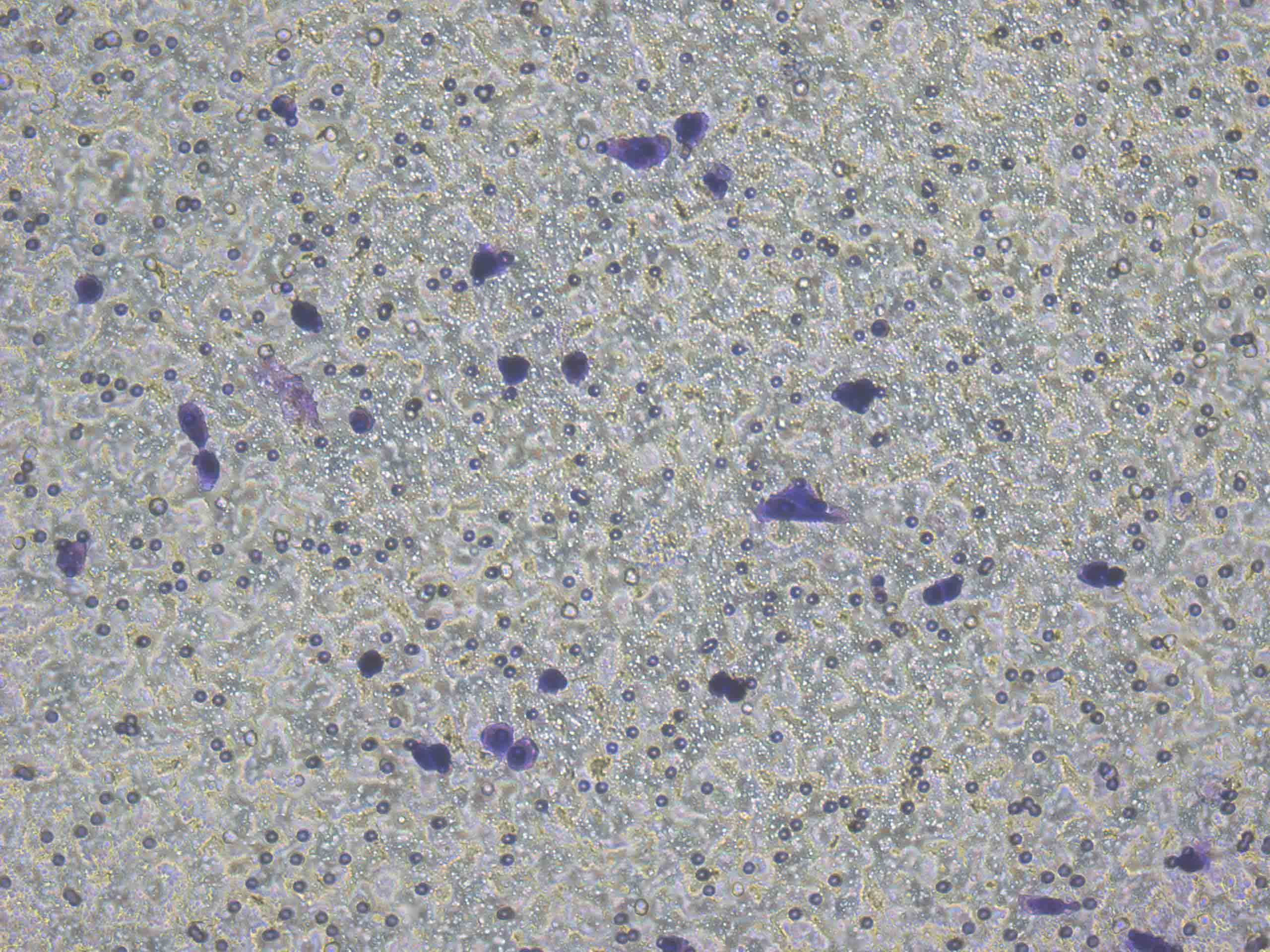

Supplement: Supplemental Information 1 [file peerj-08-8910-s001.zip › invasion_asssy/aspc-1/2/0ng-si.jpg]

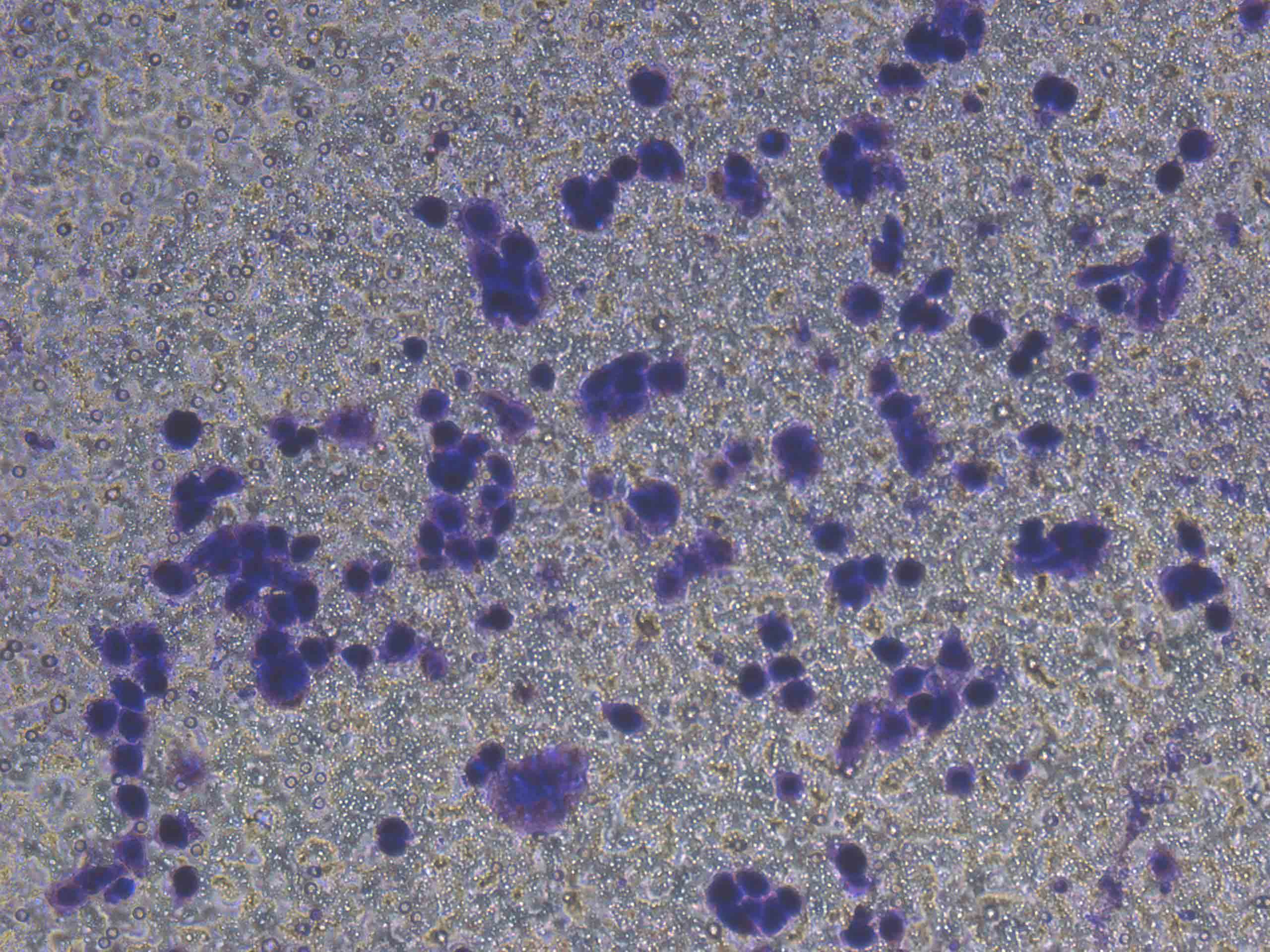

Supplement: Supplemental Information 1 [file peerj-08-8910-s001.zip › invasion_asssy/aspc-1/2/100ng-Control.jpg]

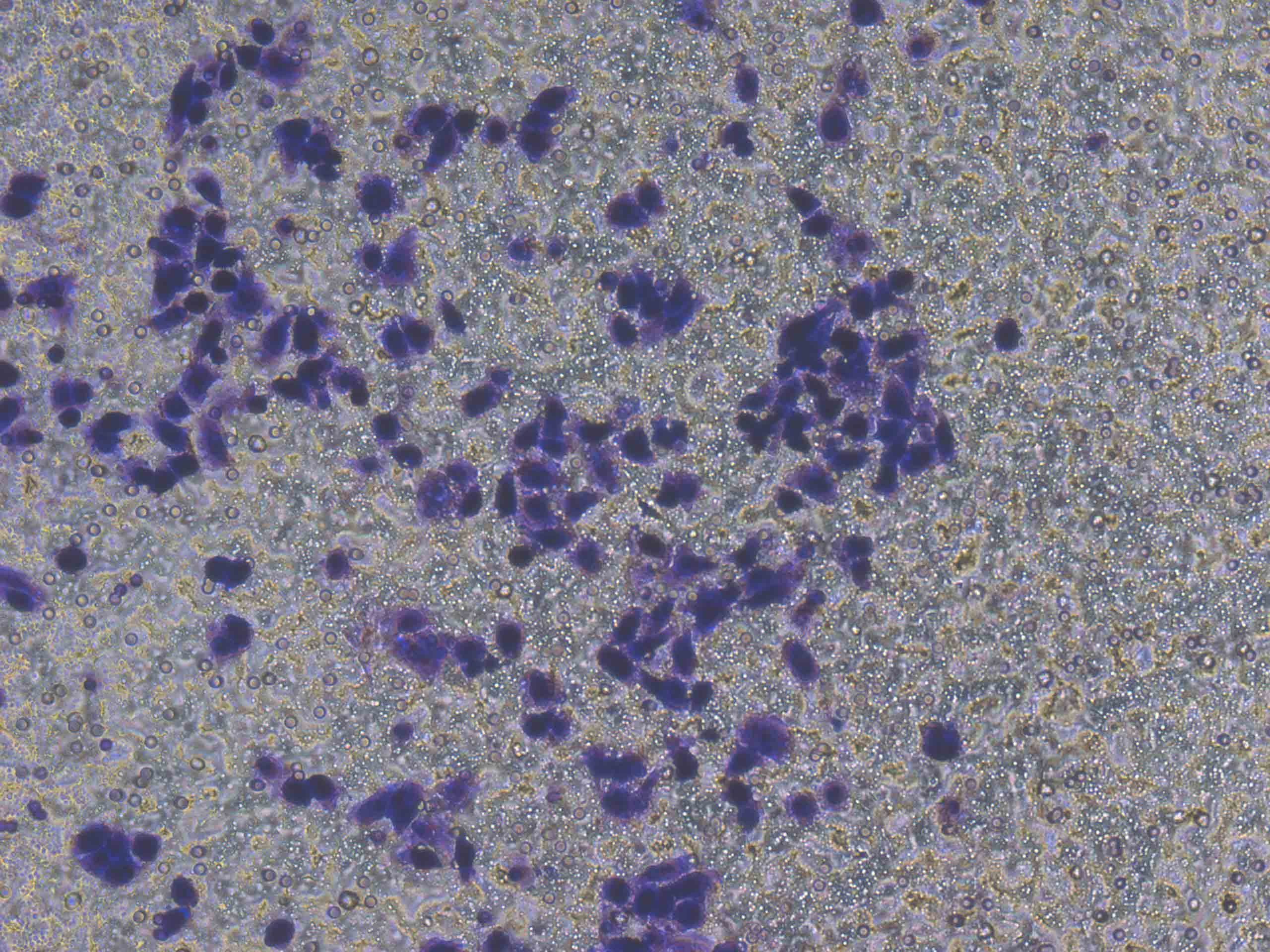

Supplement: Supplemental Information 1 [file peerj-08-8910-s001.zip › invasion_asssy/aspc-1/2/100ng-Normal.jpg]

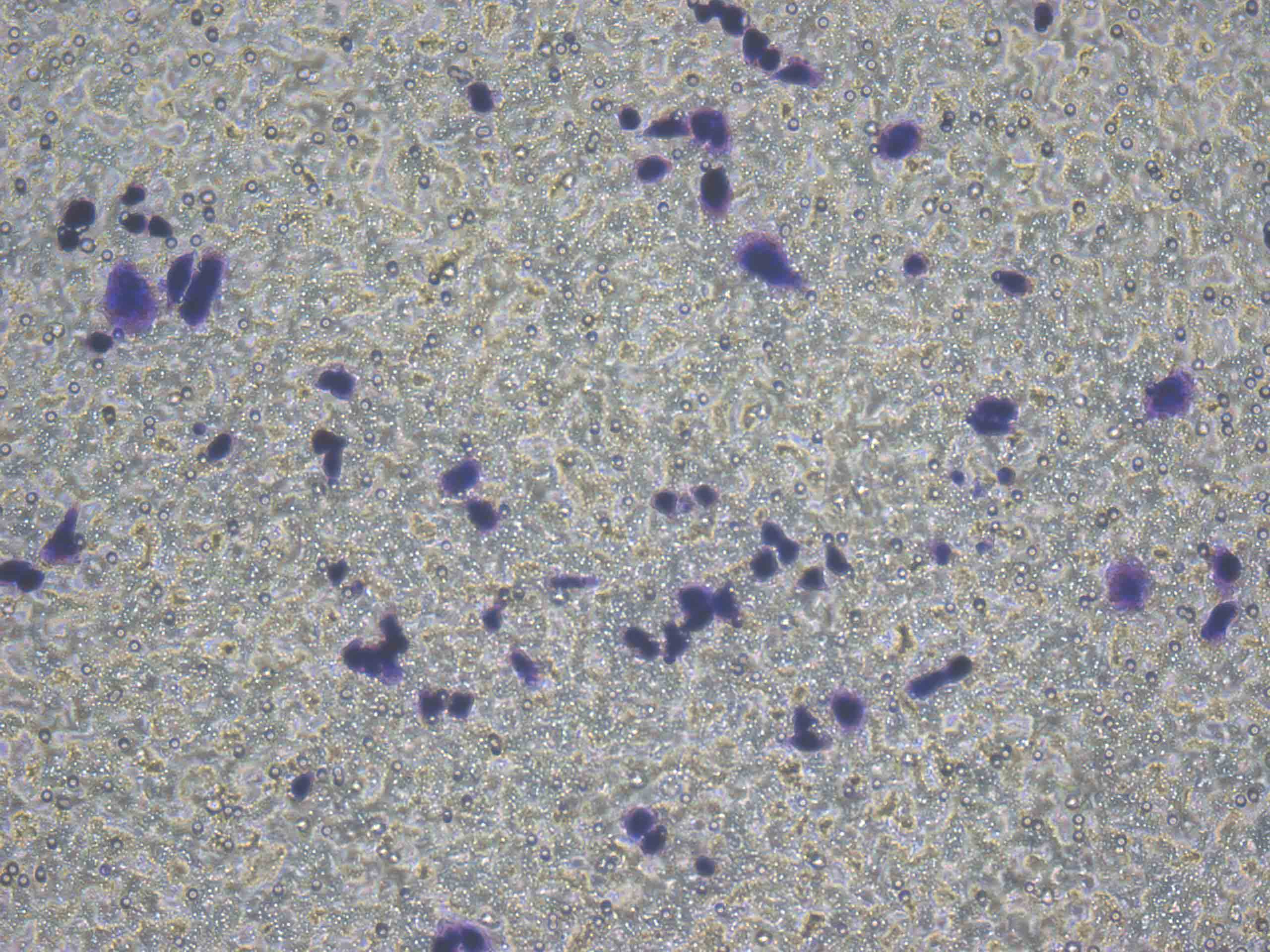

Supplement: Supplemental Information 1 [file peerj-08-8910-s001.zip › invasion_asssy/aspc-1/2/100ng-si.jpg]

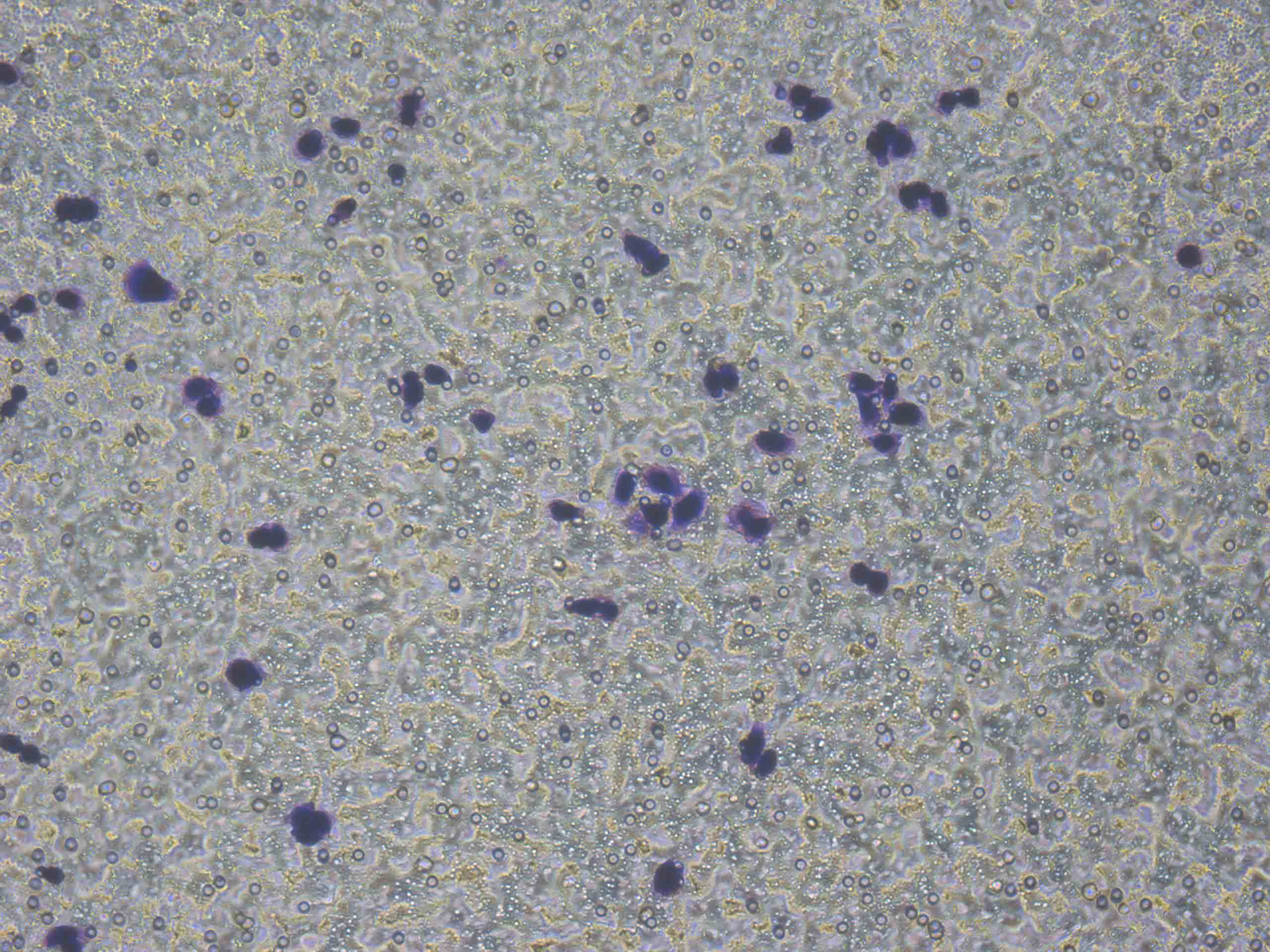

Supplement: Supplemental Information 1 [file peerj-08-8910-s001.zip › invasion_asssy/aspc-1/3/0ng-Control.jpg]

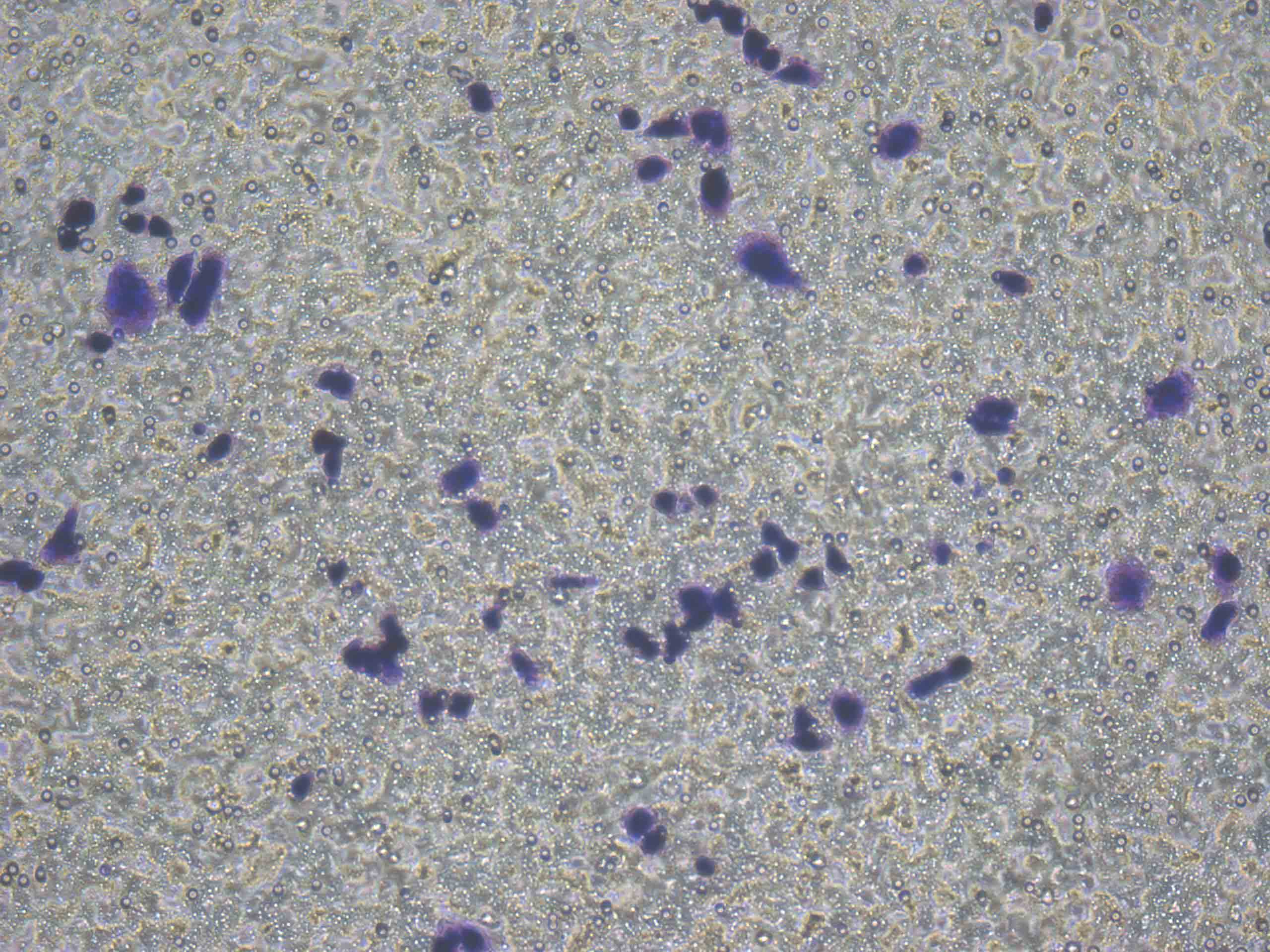

Supplement: Supplemental Information 1 [file peerj-08-8910-s001.zip › invasion_asssy/aspc-1/3/0ng-Normal.jpg]

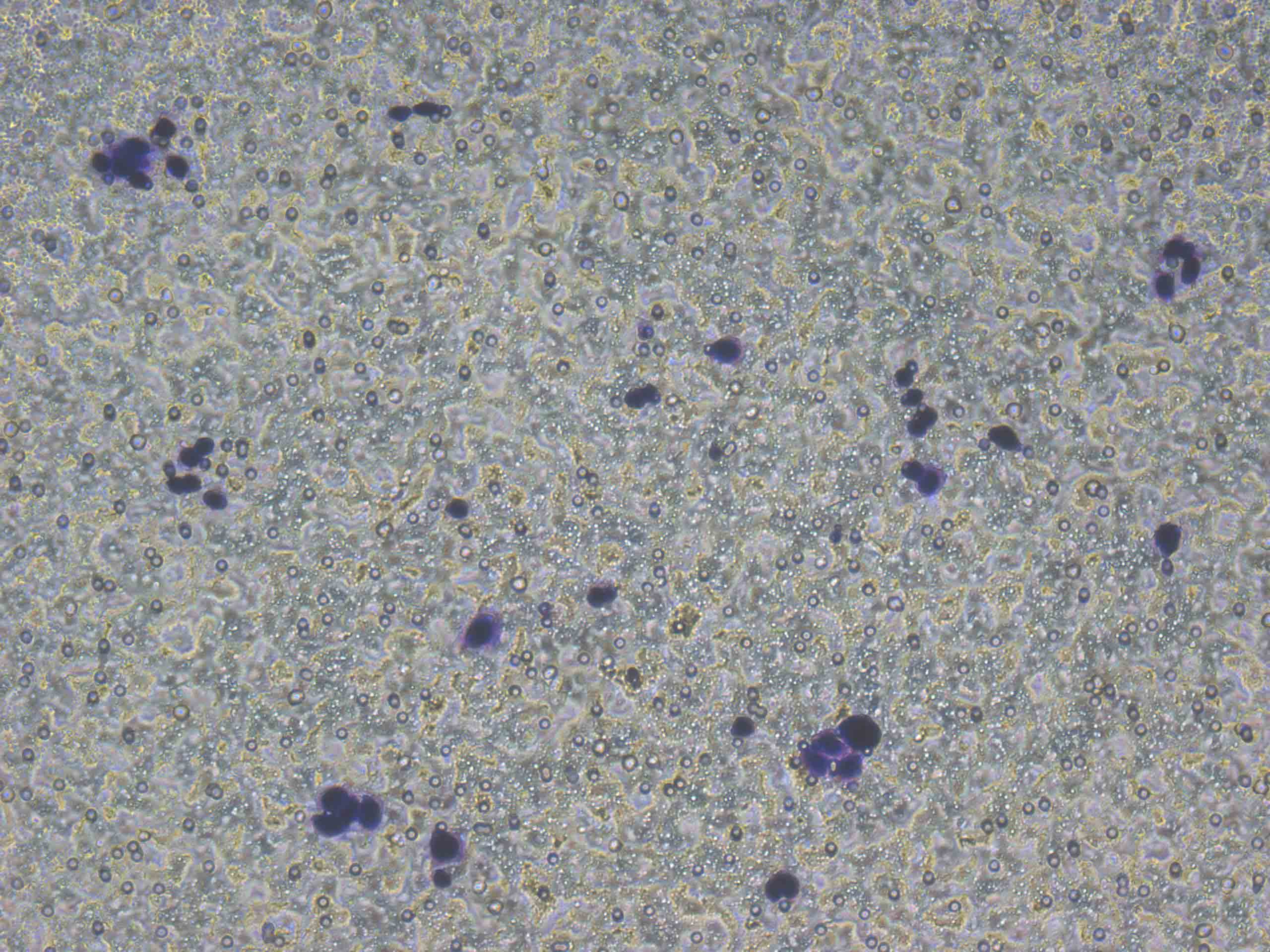

Supplement: Supplemental Information 1 [file peerj-08-8910-s001.zip › invasion_asssy/aspc-1/3/0ng-si.jpg]

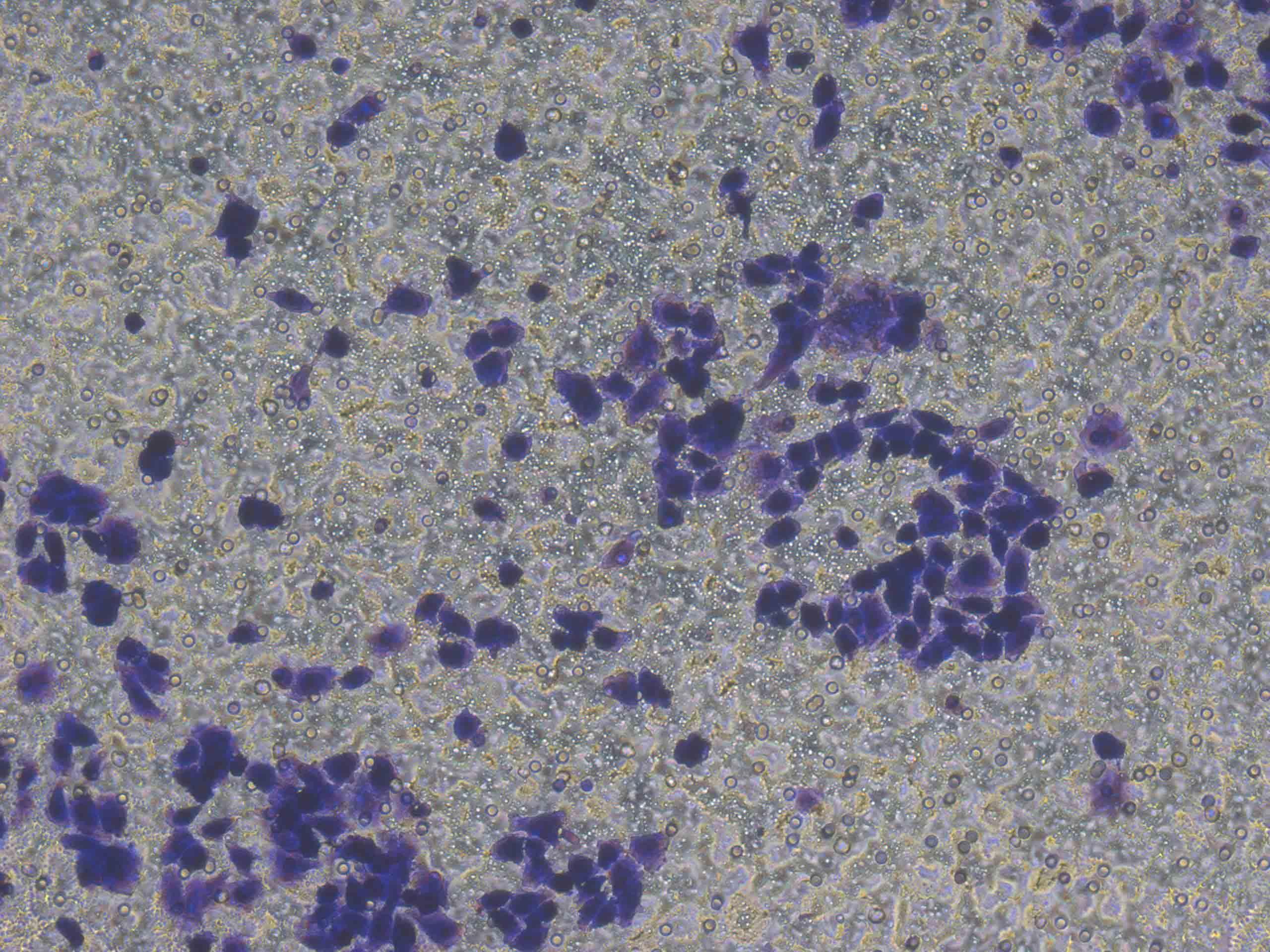

Supplement: Supplemental Information 1 [file peerj-08-8910-s001.zip › invasion_asssy/aspc-1/3/100ng-Control.jpg]

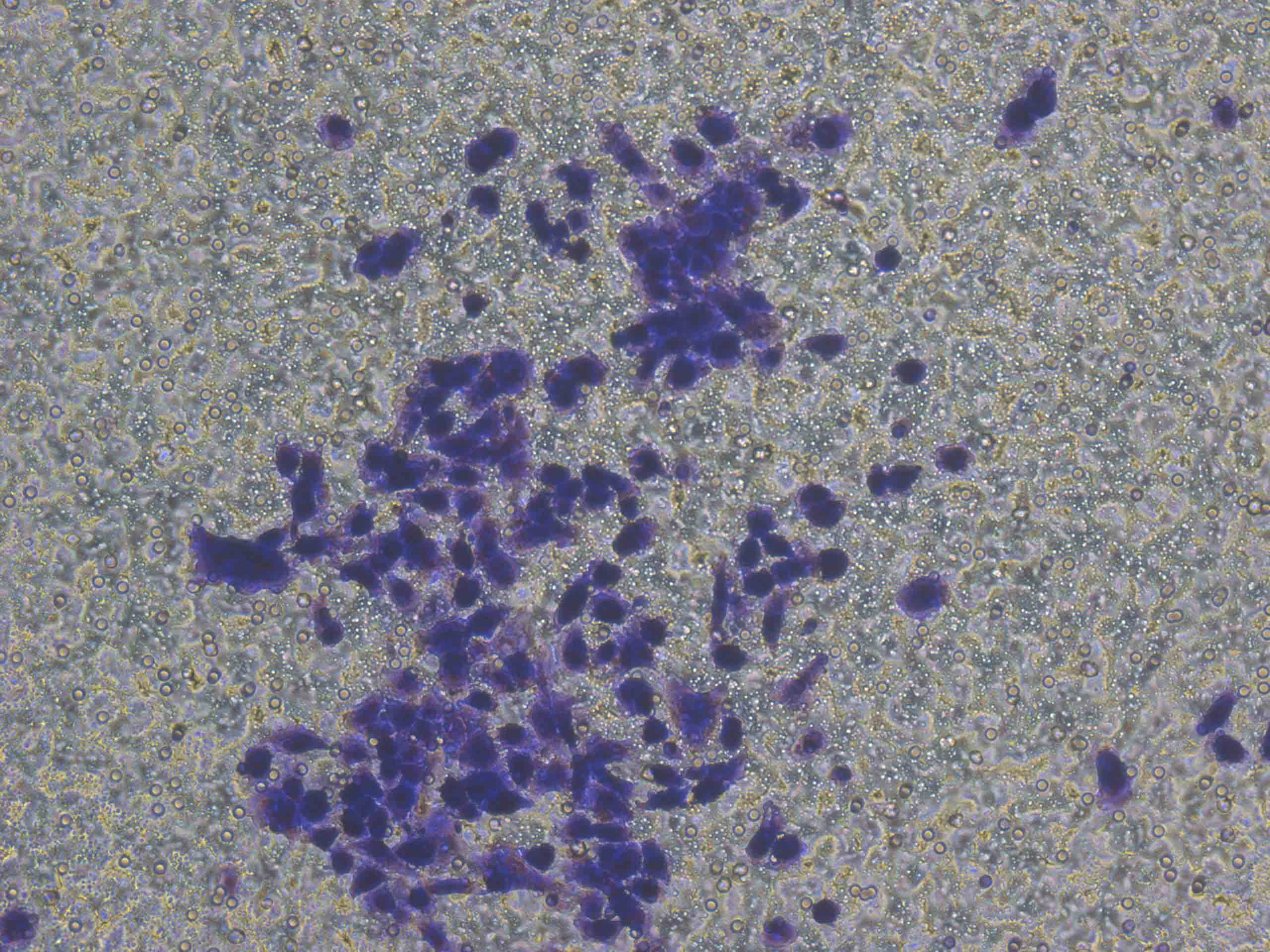

Supplement: Supplemental Information 1 [file peerj-08-8910-s001.zip › invasion_asssy/aspc-1/3/100ng-Normal.jpg]

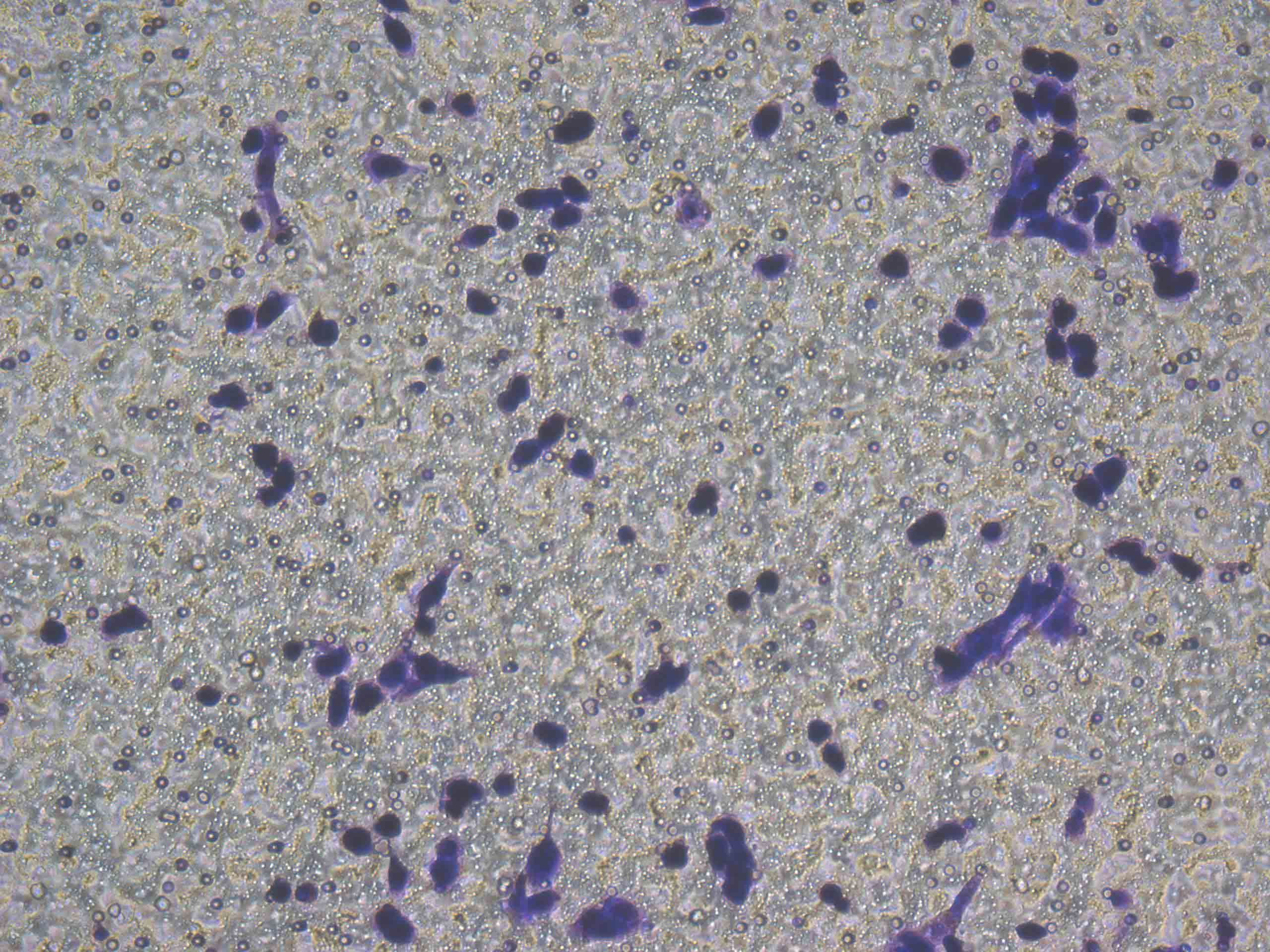

Supplement: Supplemental Information 1 [file peerj-08-8910-s001.zip › invasion_asssy/aspc-1/3/100ng-si.jpg]

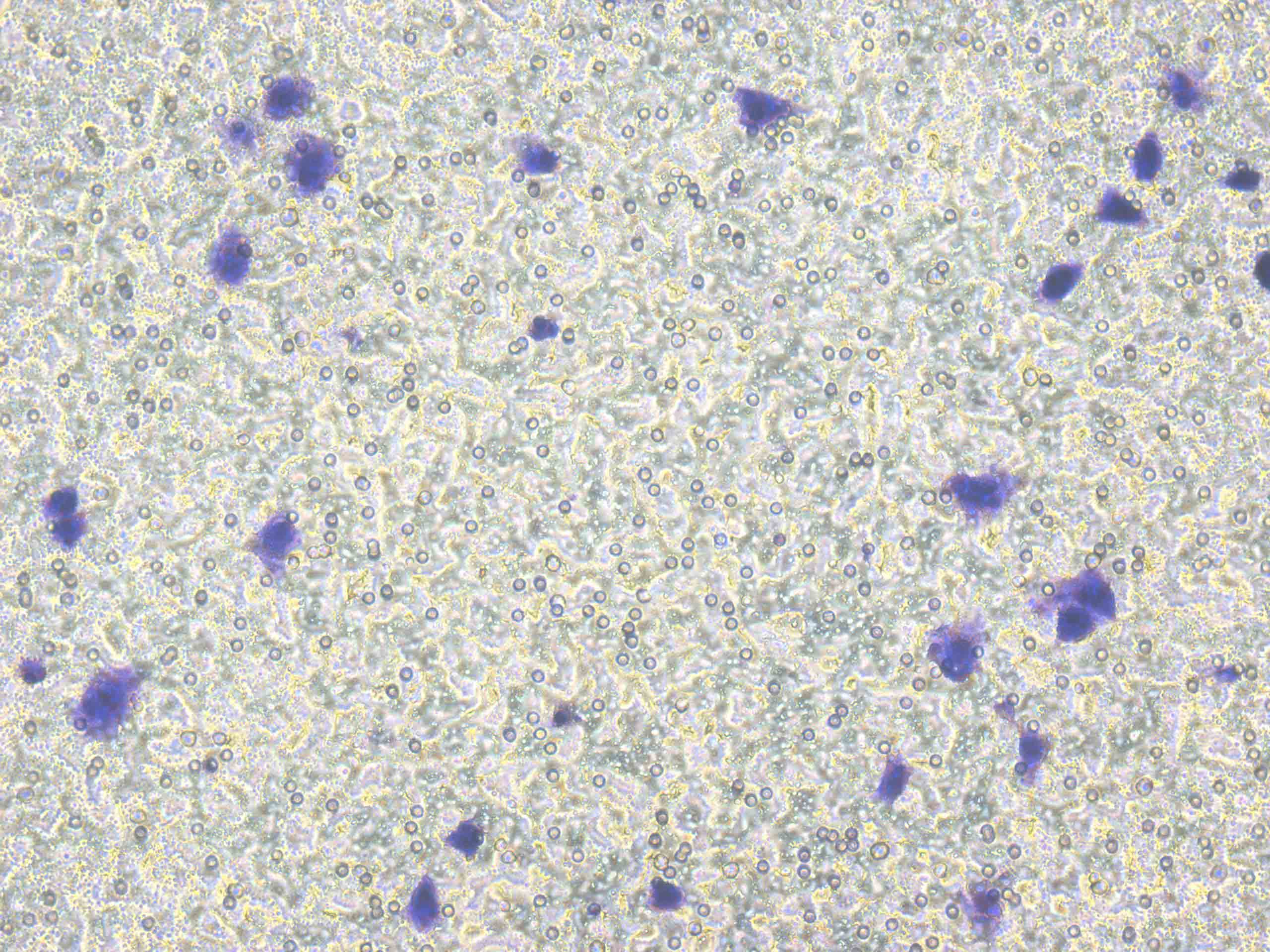

Supplement: Supplemental Information 1 [file peerj-08-8910-s001.zip › invasion_asssy/panc-1/1/0ng-Control.jpg]

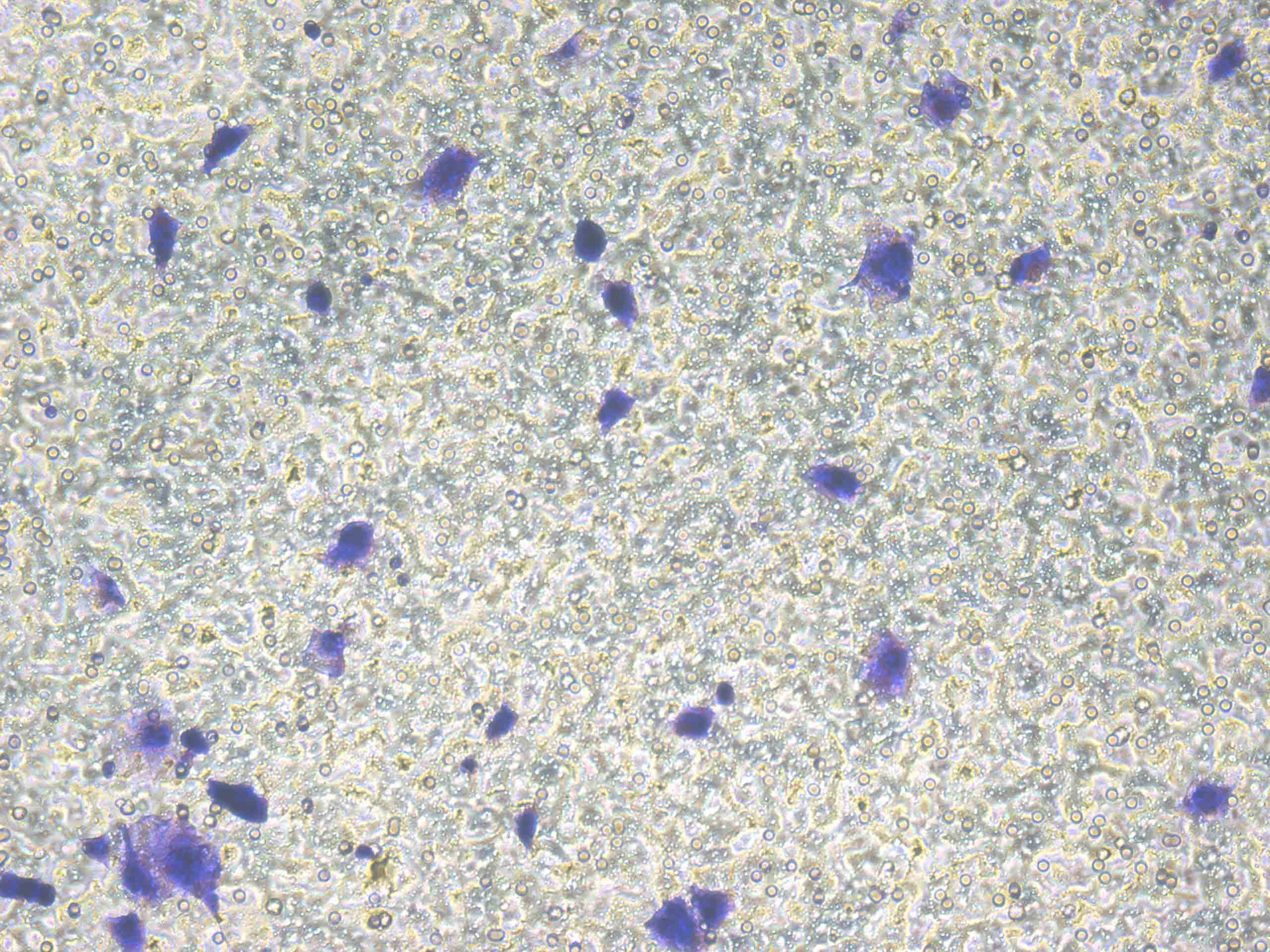

Supplement: Supplemental Information 1 [file peerj-08-8910-s001.zip › invasion_asssy/panc-1/1/0ng-Normal.jpg]

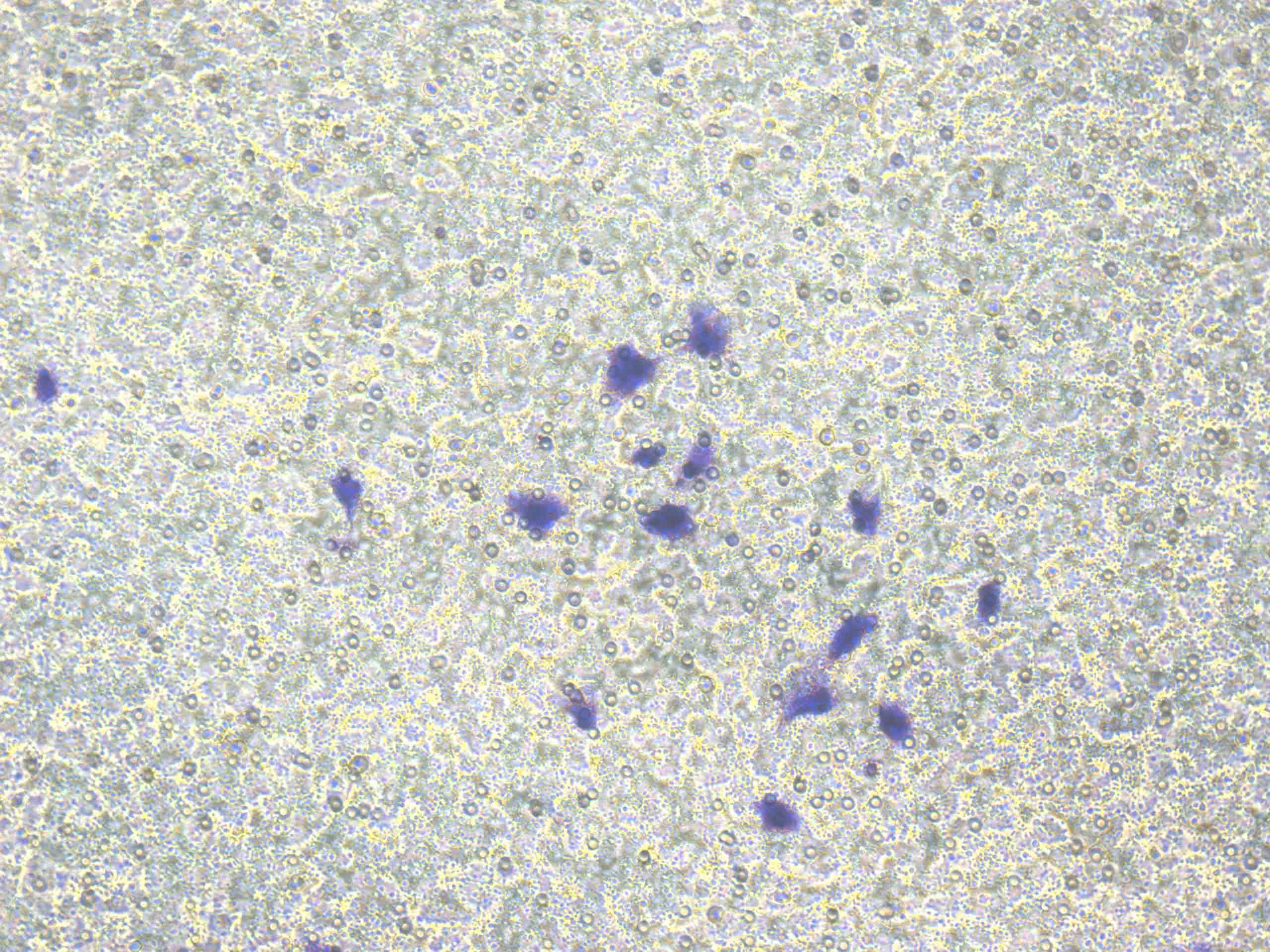

Supplement: Supplemental Information 1 [file peerj-08-8910-s001.zip › invasion_asssy/panc-1/1/0ng-Si.jpg]

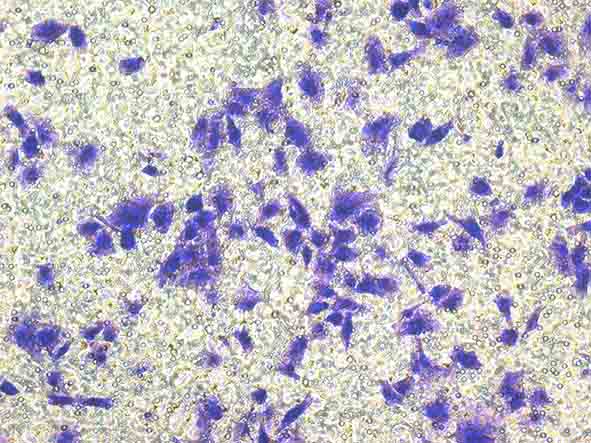

Supplement: Supplemental Information 1 [file peerj-08-8910-s001.zip › invasion_asssy/panc-1/1/100ng-Control.jpg]

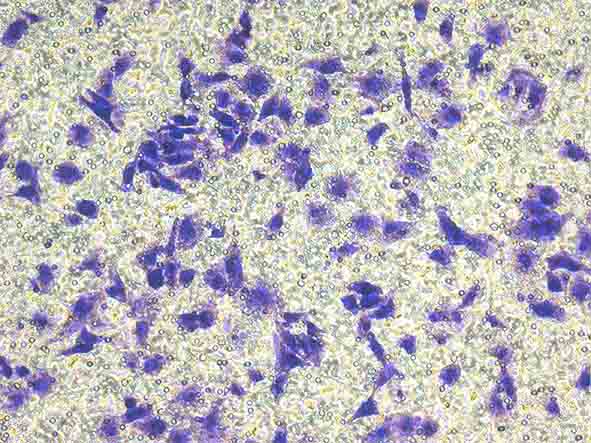

Supplement: Supplemental Information 1 [file peerj-08-8910-s001.zip › invasion_asssy/panc-1/1/100ng-Normal.jpg]

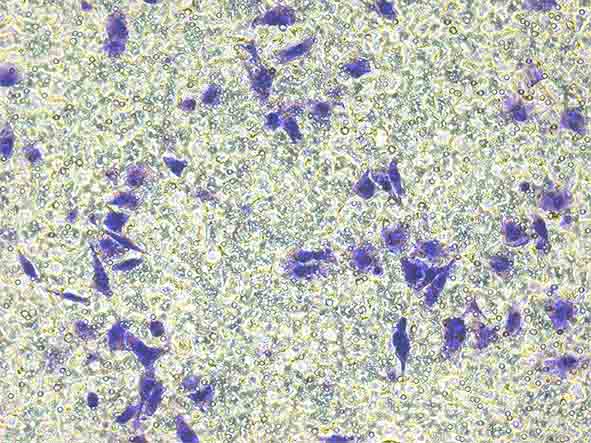

Supplement: Supplemental Information 1 [file peerj-08-8910-s001.zip › invasion_asssy/panc-1/1/100ng-Si.jpg]

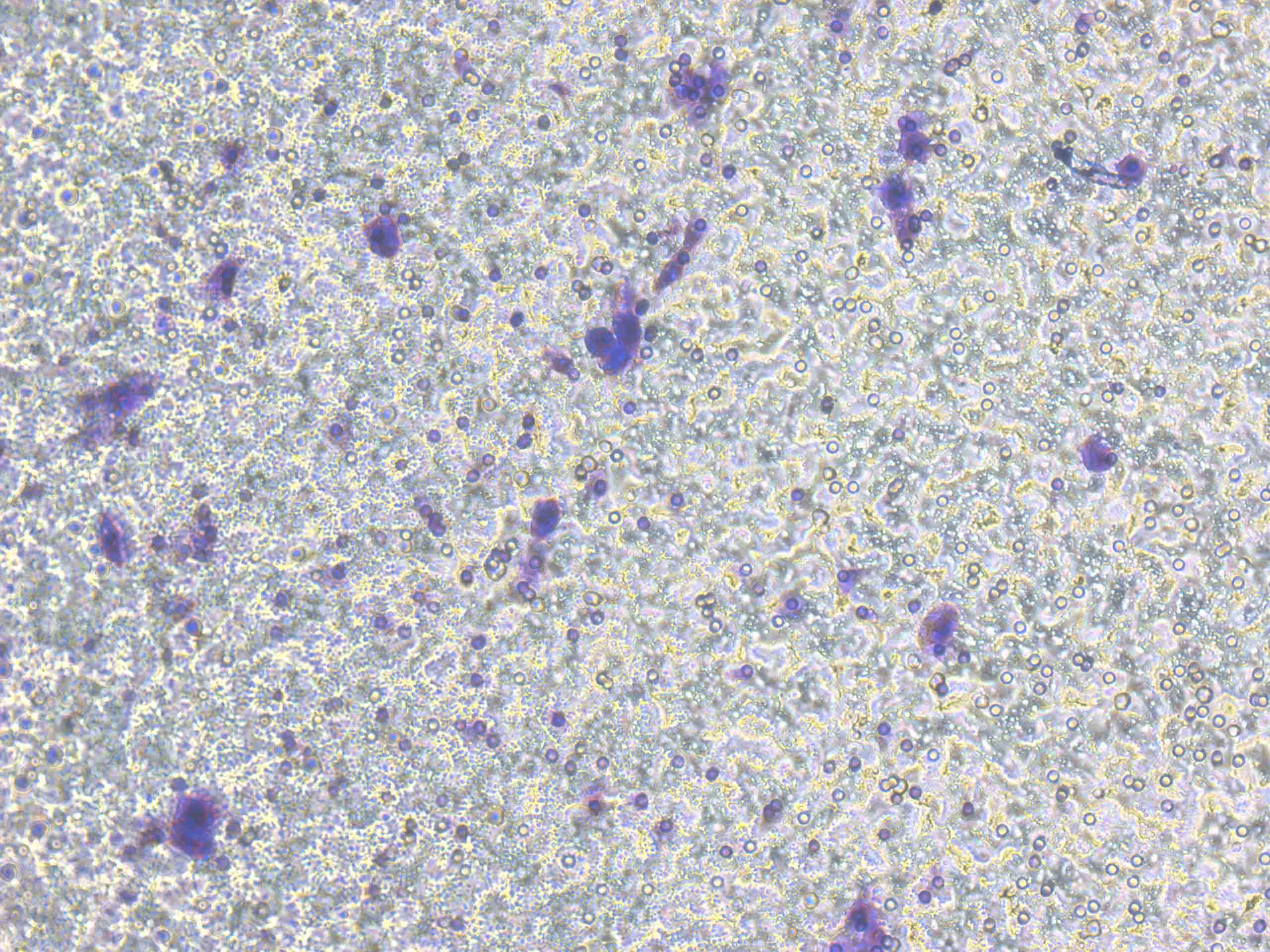

Supplement: Supplemental Information 1 [file peerj-08-8910-s001.zip › invasion_asssy/panc-1/2/0ng-Control.jpg]

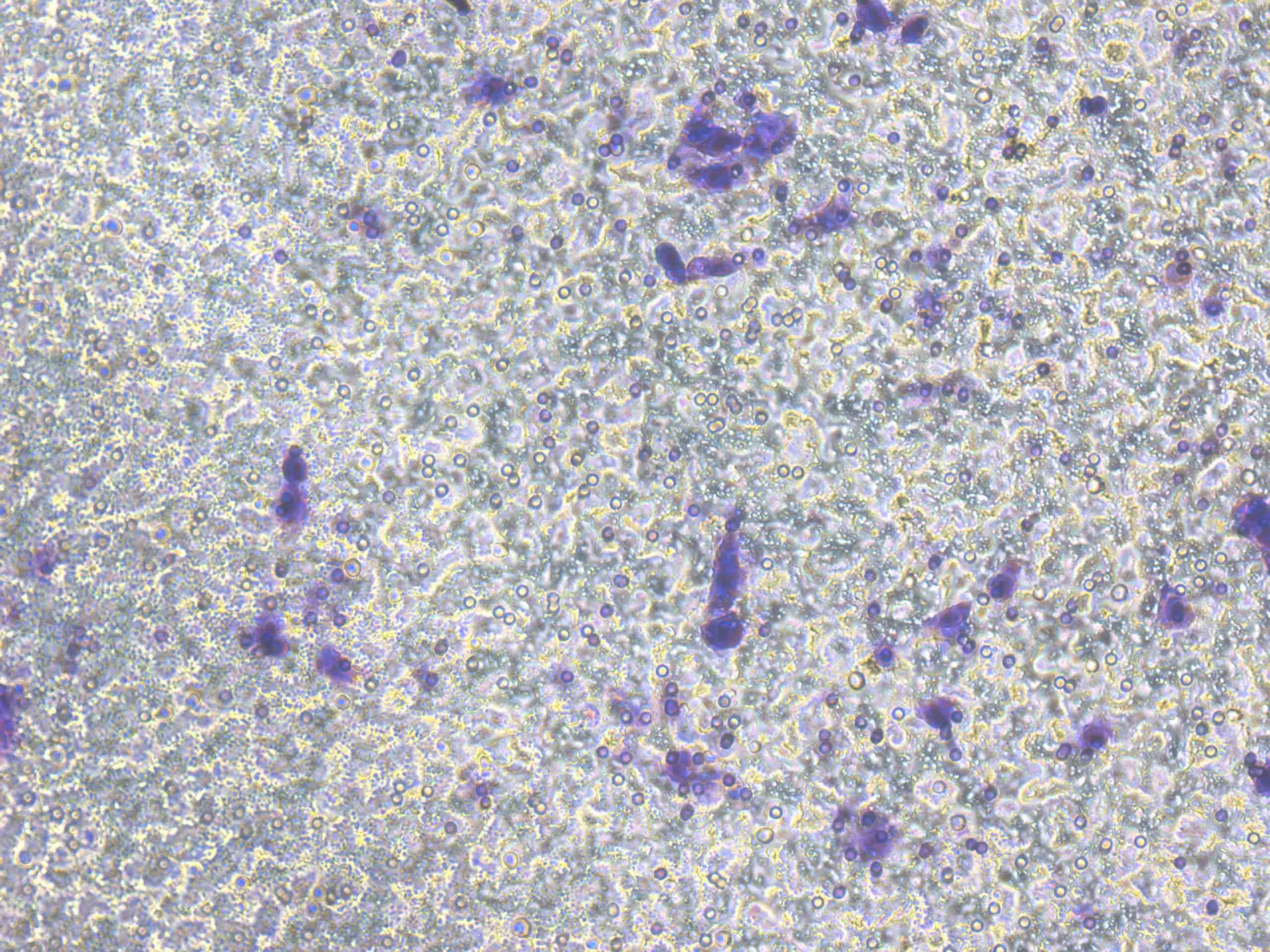

Supplement: Supplemental Information 1 [file peerj-08-8910-s001.zip › invasion_asssy/panc-1/2/0ng-Normal.jpg]

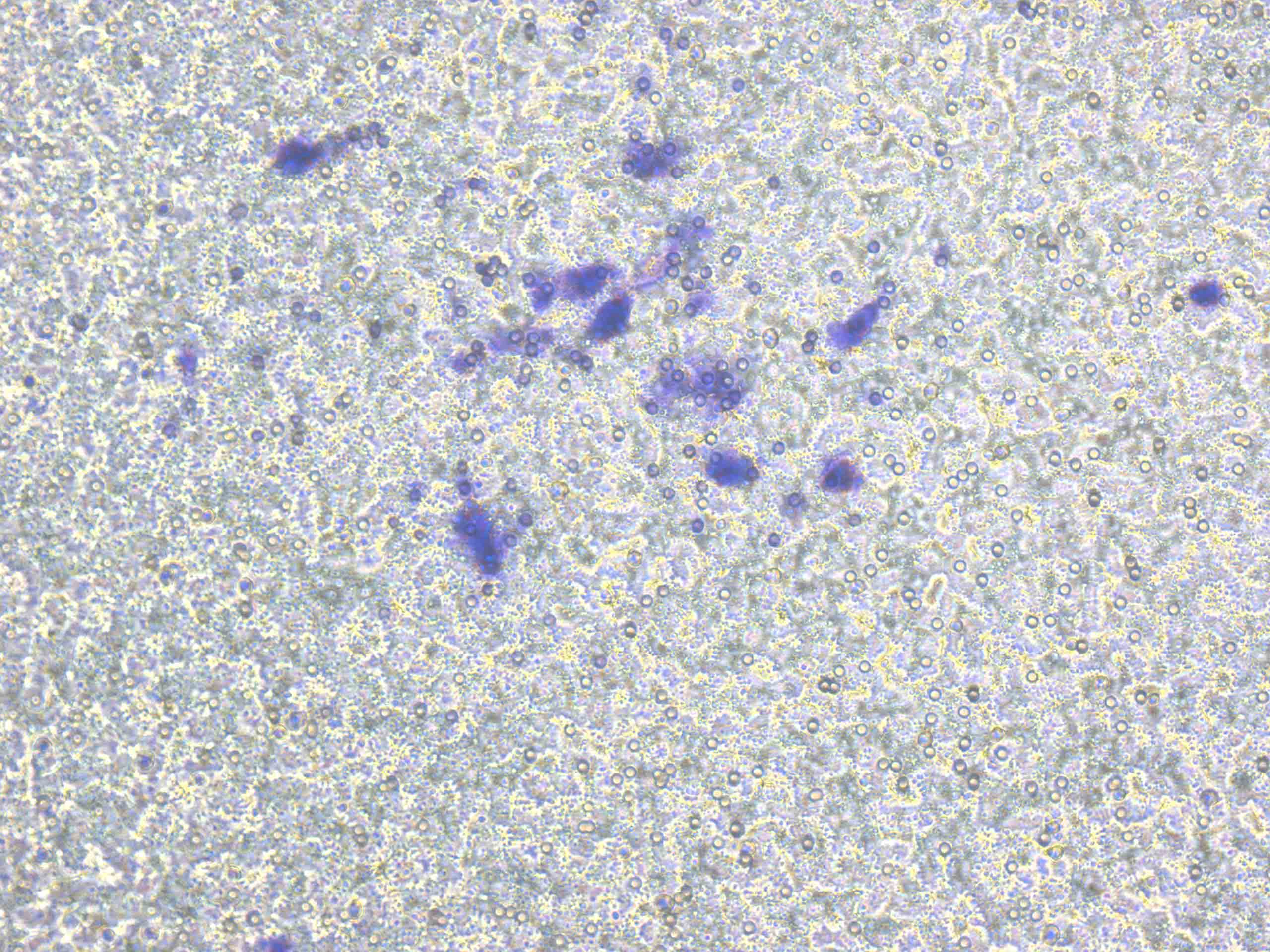

Supplement: Supplemental Information 1 [file peerj-08-8910-s001.zip › invasion_asssy/panc-1/2/0ng-si.jpg]

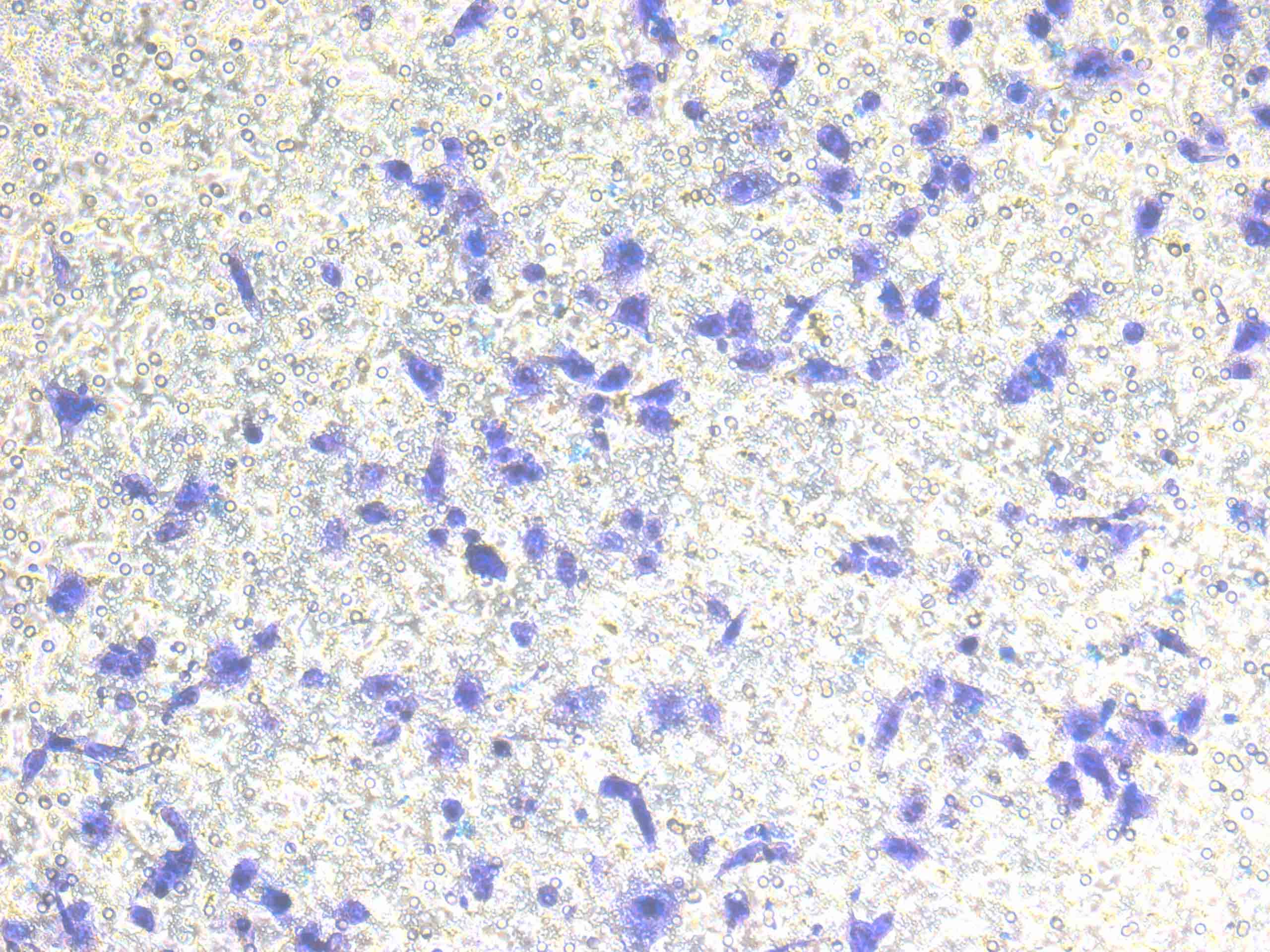

Supplement: Supplemental Information 1 [file peerj-08-8910-s001.zip › invasion_asssy/panc-1/2/100ng-Control.jpg]

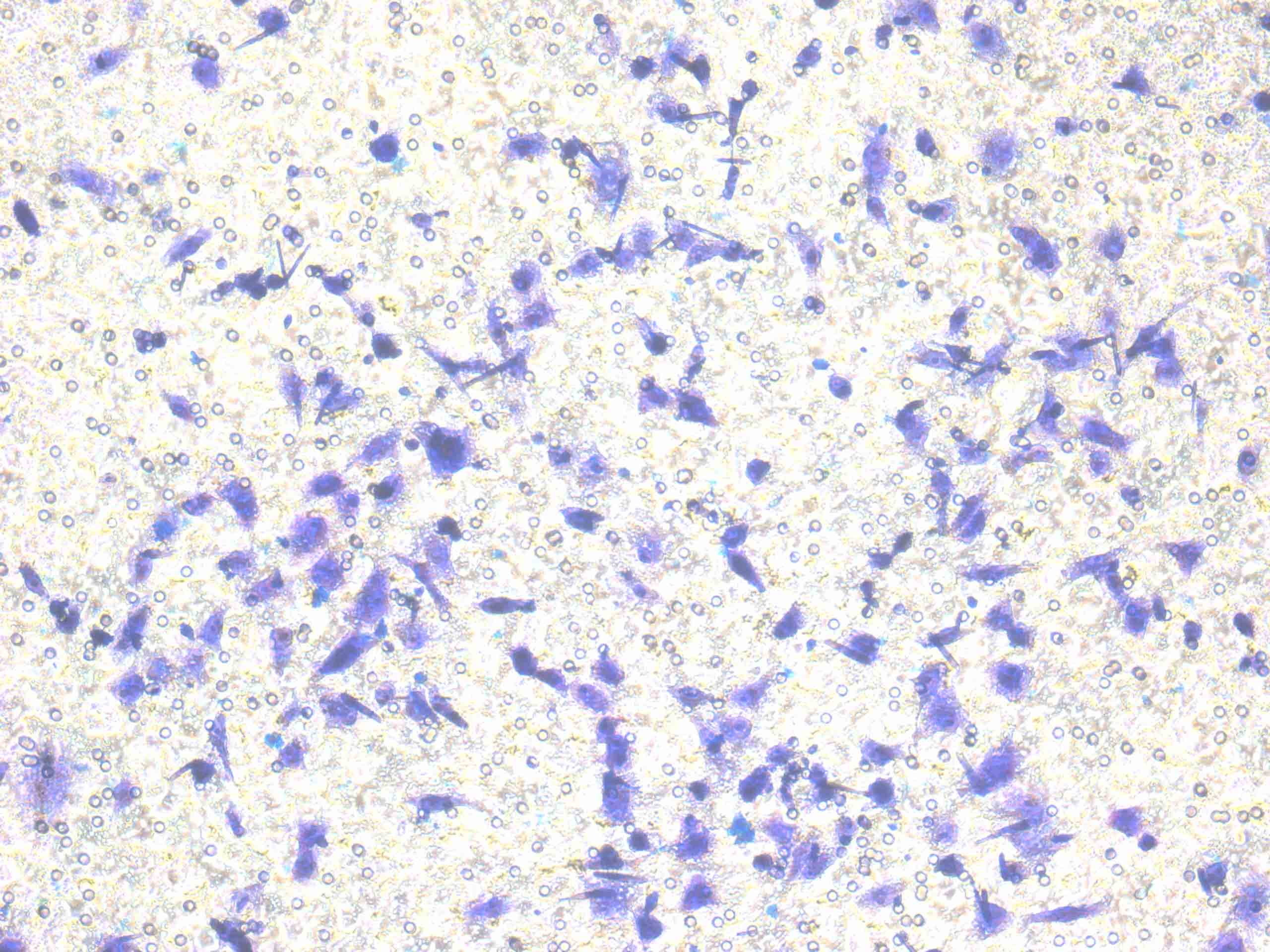

Supplement: Supplemental Information 1 [file peerj-08-8910-s001.zip › invasion_asssy/panc-1/2/100ng-Normal.jpg]

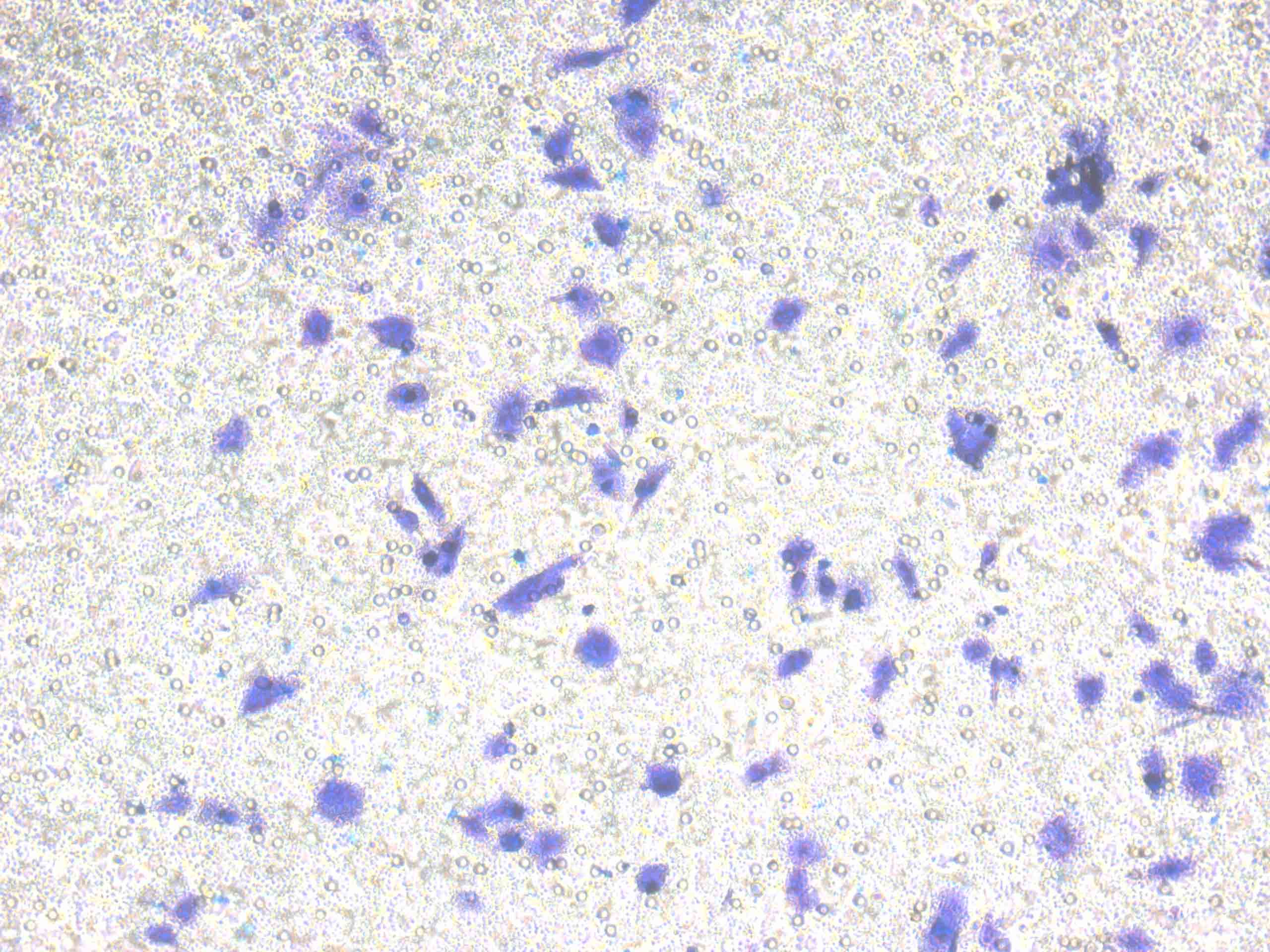

Supplement: Supplemental Information 1 [file peerj-08-8910-s001.zip › invasion_asssy/panc-1/2/100ng-Si.jpg]

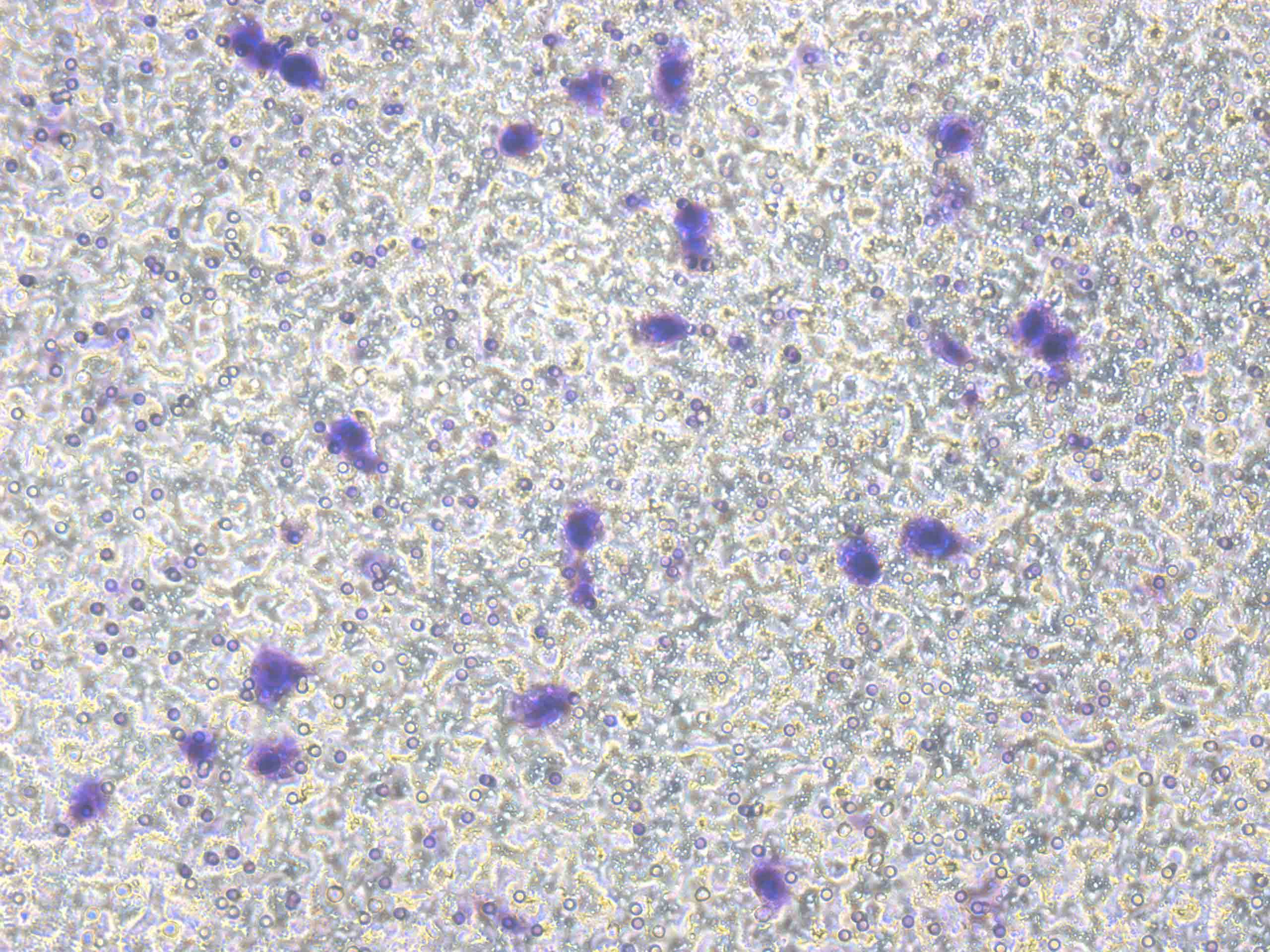

Supplement: Supplemental Information 1 [file peerj-08-8910-s001.zip › invasion_asssy/panc-1/3/0ng-Control.jpg]

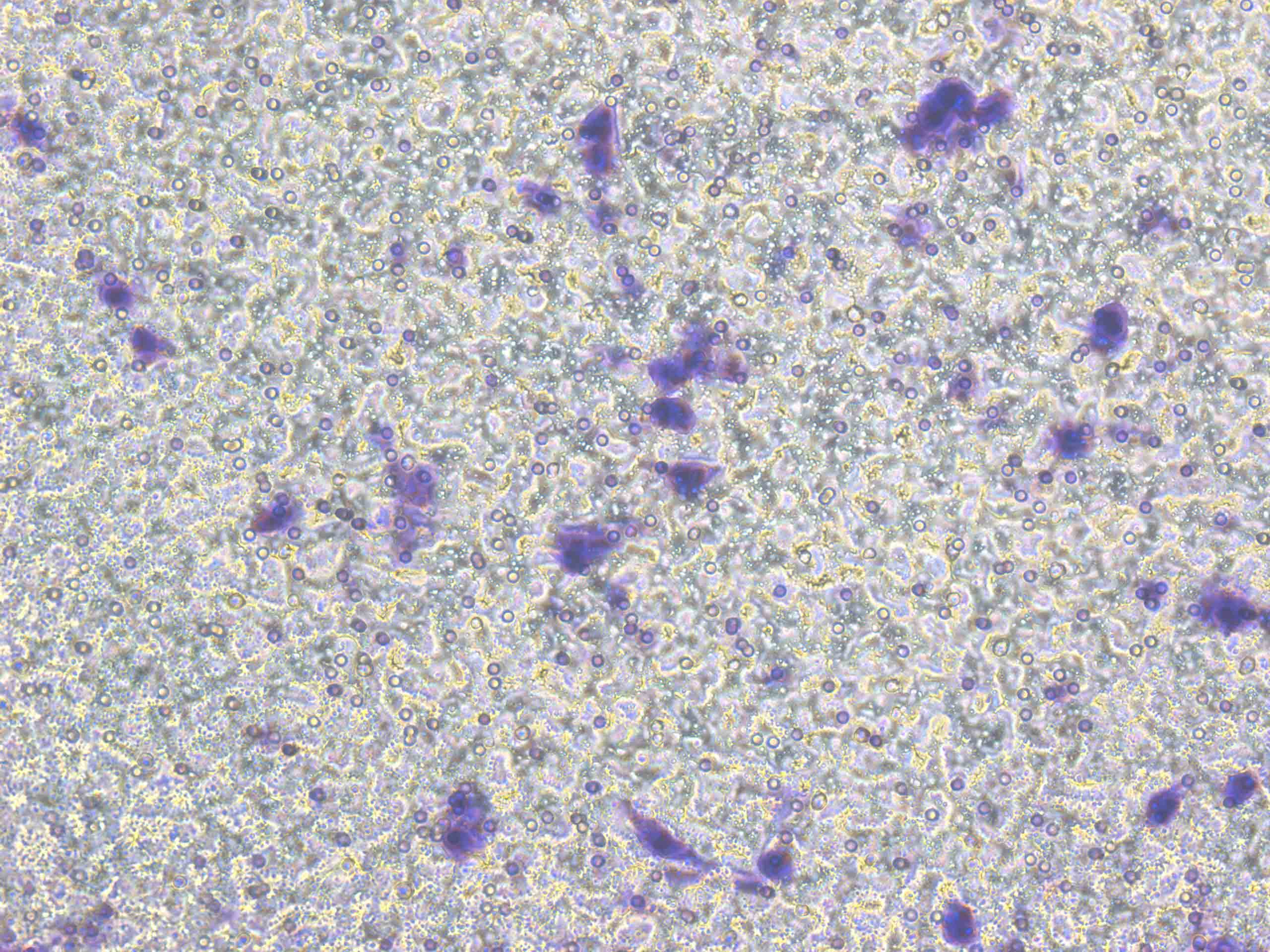

Supplement: Supplemental Information 1 [file peerj-08-8910-s001.zip › invasion_asssy/panc-1/3/0ng-Normal.jpg]

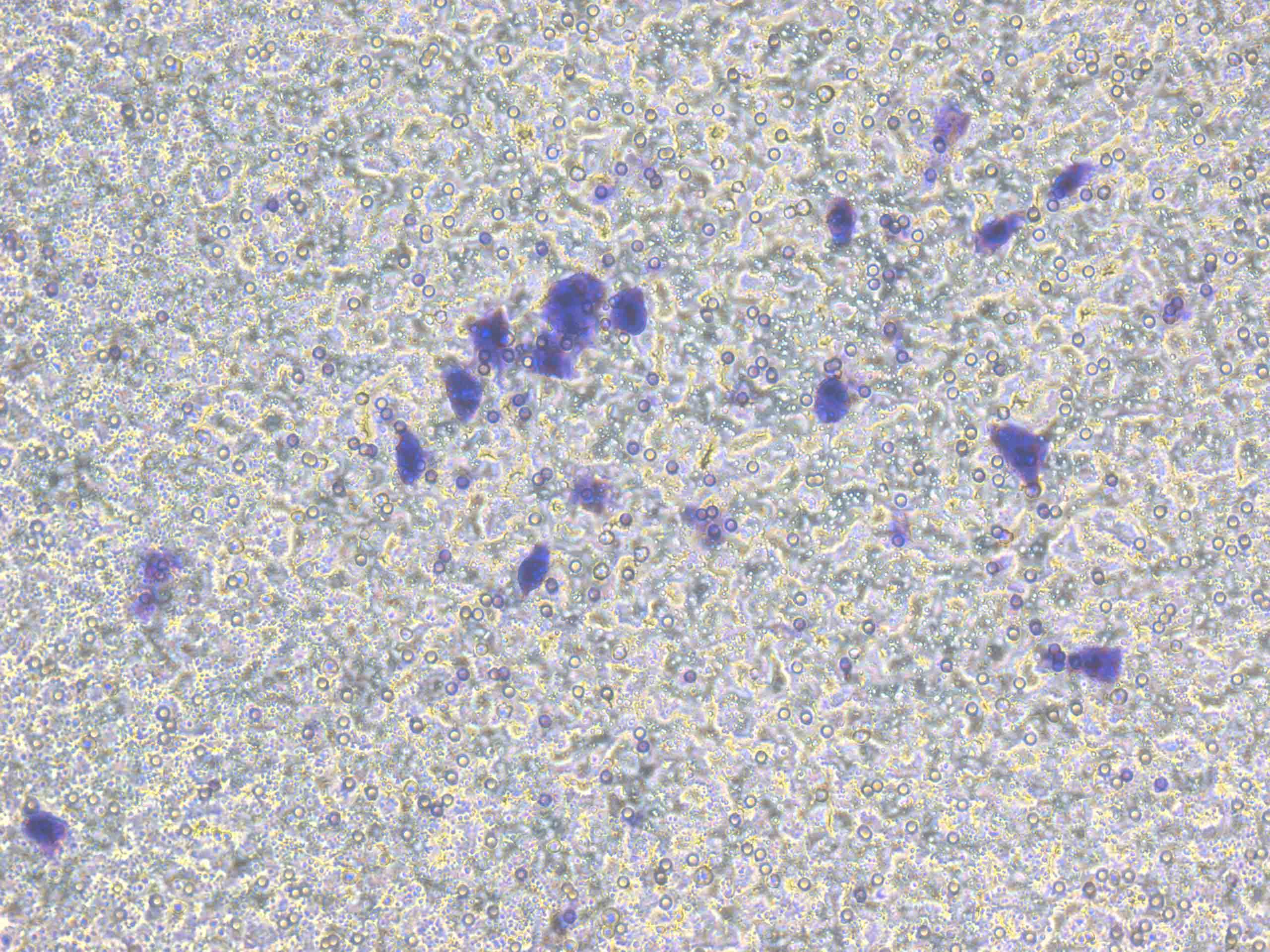

Supplement: Supplemental Information 1 [file peerj-08-8910-s001.zip › invasion_asssy/panc-1/3/0ng-si.jpg]

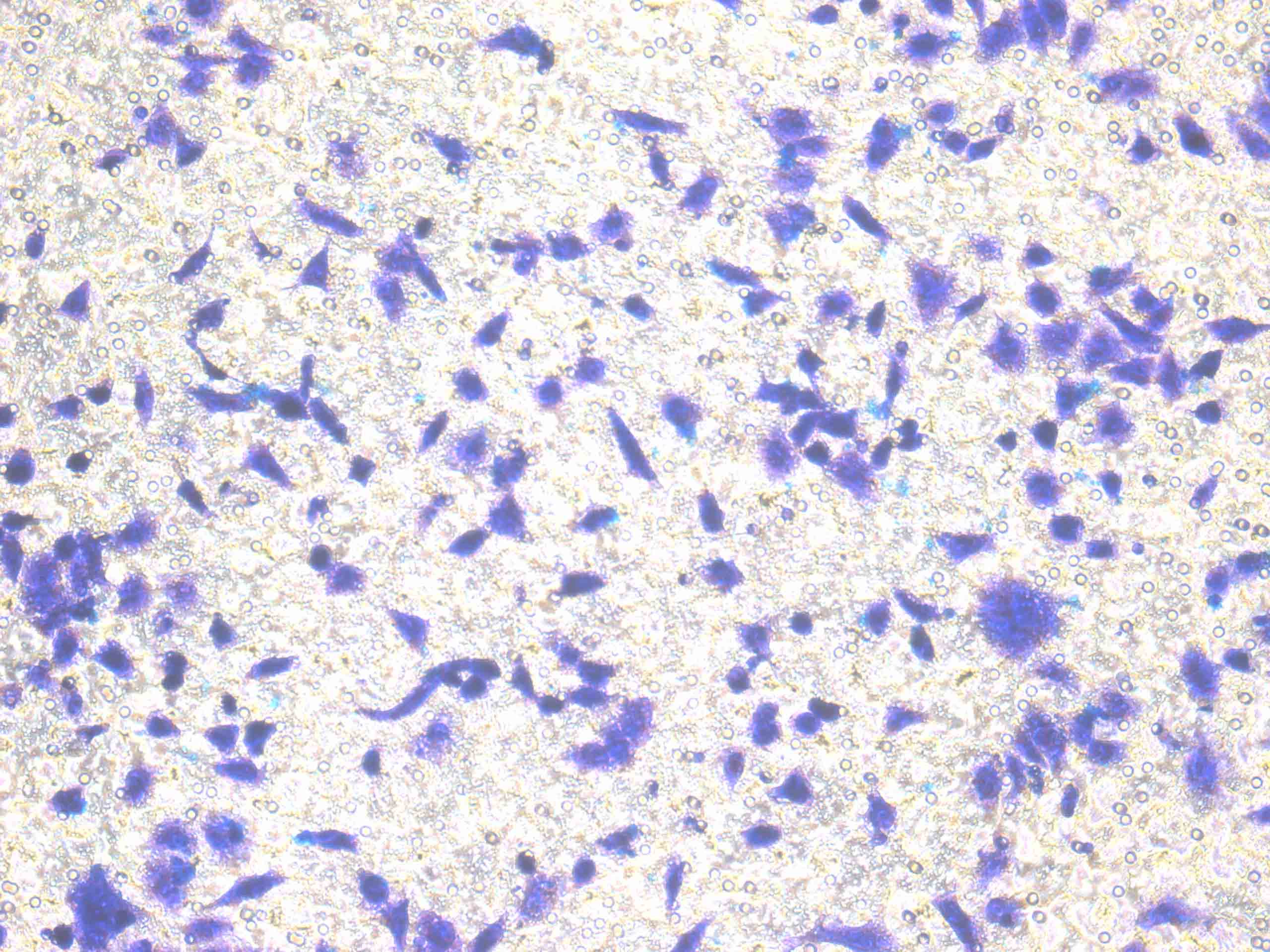

Supplement: Supplemental Information 1 [file peerj-08-8910-s001.zip › invasion_asssy/panc-1/3/100ng-Control.jpg]

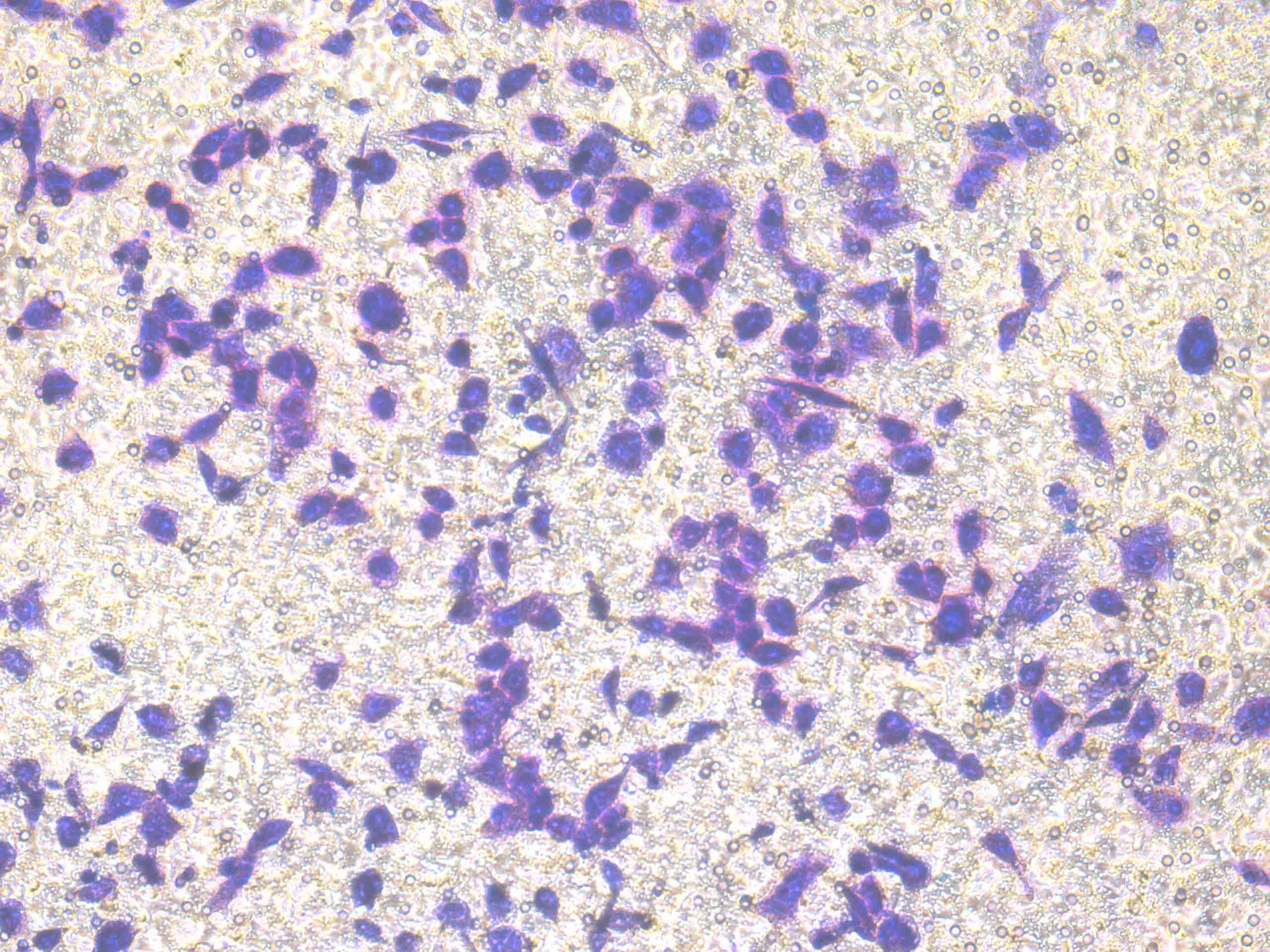

Supplement: Supplemental Information 1 [file peerj-08-8910-s001.zip › invasion_asssy/panc-1/3/100ng-Normal.jpg]

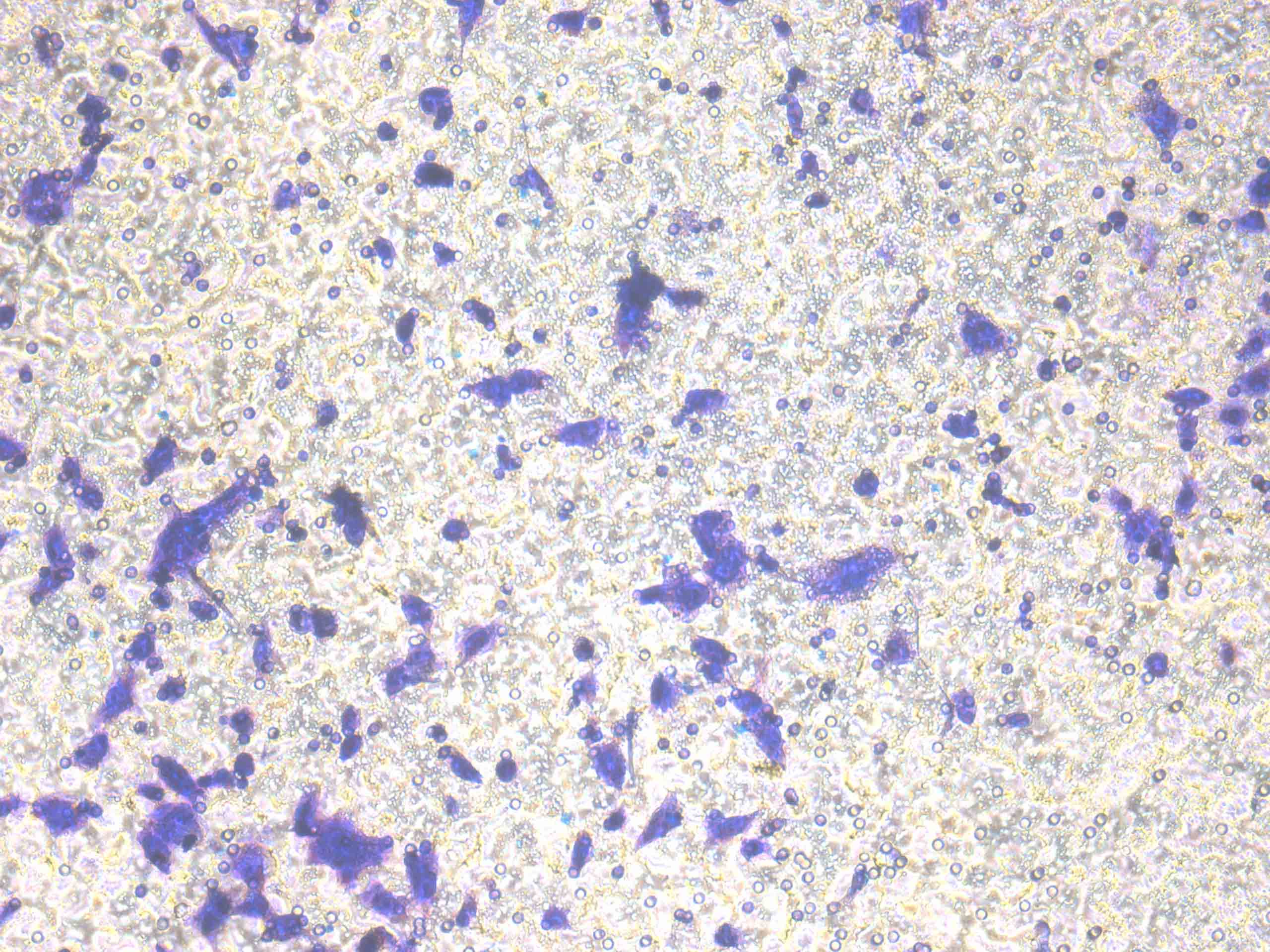

Supplement: Supplemental Information 1 [file peerj-08-8910-s001.zip › invasion_asssy/panc-1/3/100ng-si.jpg]

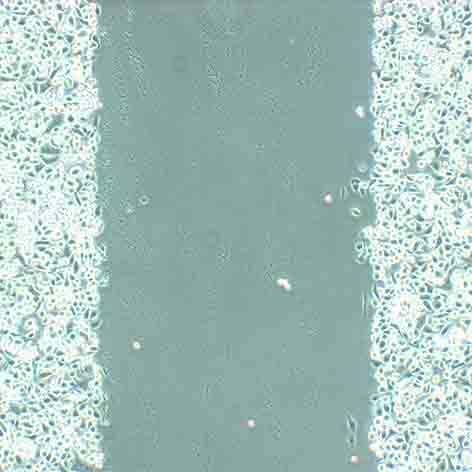

Supplement: Supplemental Information 1 [file peerj-08-8910-s001.zip › scratching_assay/acpc-1/1/aspc--0h--control.jpg]

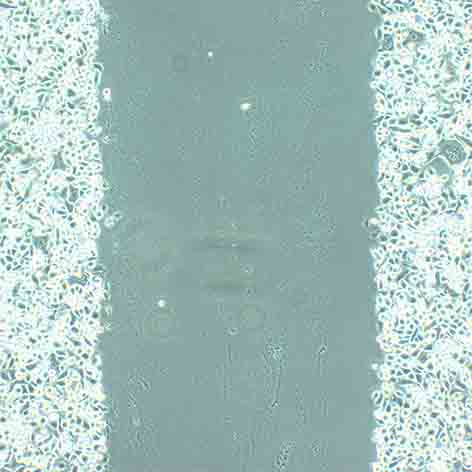

Supplement: Supplemental Information 1 [file peerj-08-8910-s001.zip › scratching_assay/acpc-1/1/aspc--0h--normal.jpg]

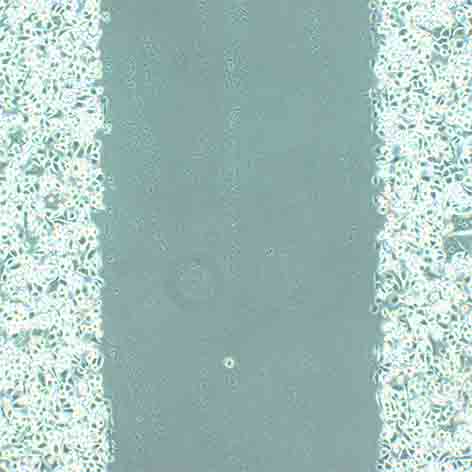

Supplement: Supplemental Information 1 [file peerj-08-8910-s001.zip › scratching_assay/acpc-1/1/aspc--0h--si.jpg]

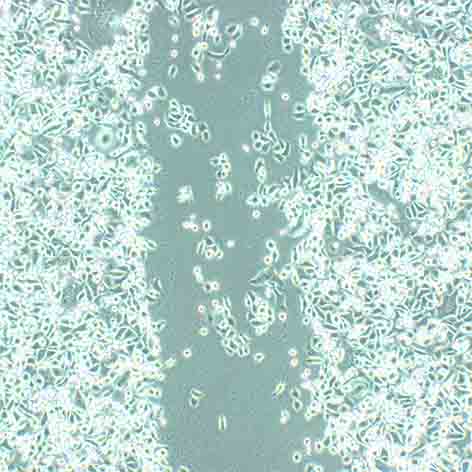

Supplement: Supplemental Information 1 [file peerj-08-8910-s001.zip › scratching_assay/acpc-1/1/aspc--12h--control.jpg]

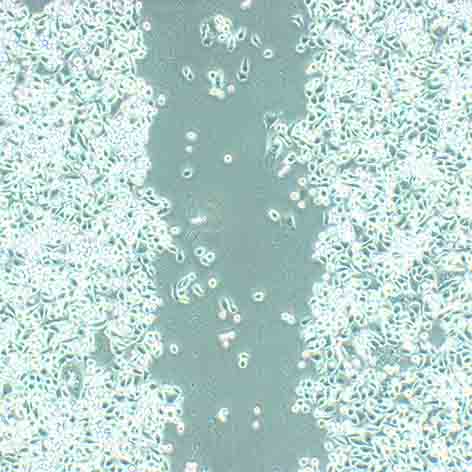

Supplement: Supplemental Information 1 [file peerj-08-8910-s001.zip › scratching_assay/acpc-1/1/aspc--12h--normal.jpg]

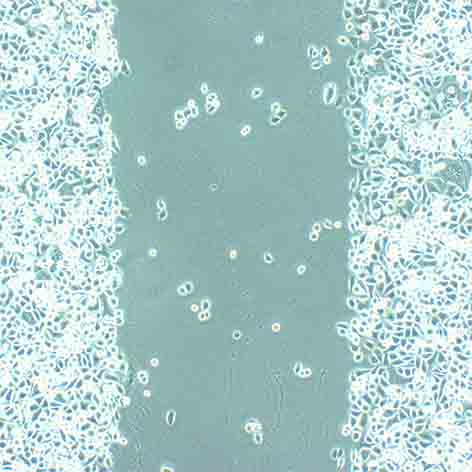

Supplement: Supplemental Information 1 [file peerj-08-8910-s001.zip › scratching_assay/acpc-1/1/aspc--12h--si.jpg]

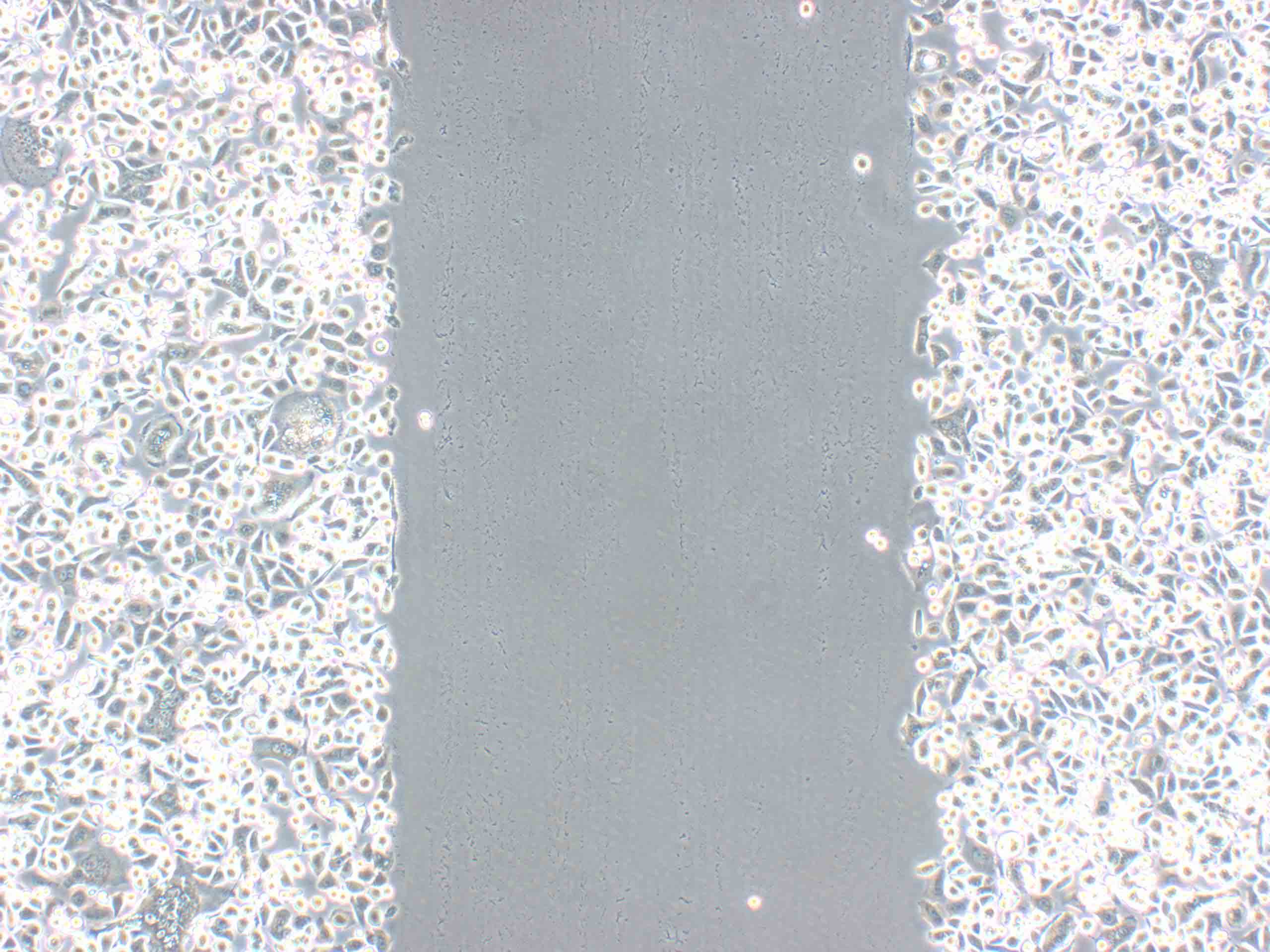

Supplement: Supplemental Information 1 [file peerj-08-8910-s001.zip › scratching_assay/acpc-1/2/Control-0h.jpg]

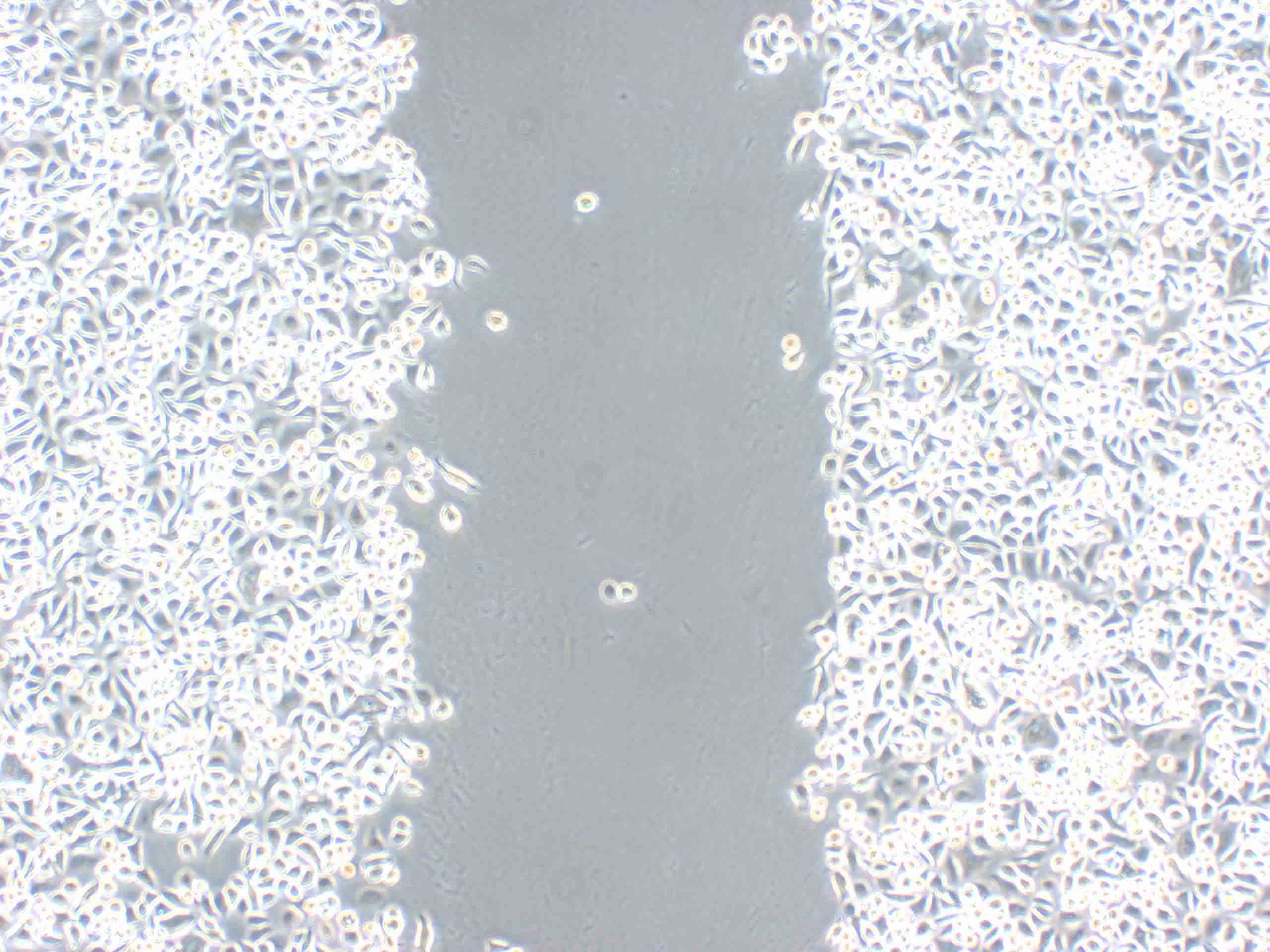

Supplement: Supplemental Information 1 [file peerj-08-8910-s001.zip › scratching_assay/acpc-1/2/Control-12h.jpg]

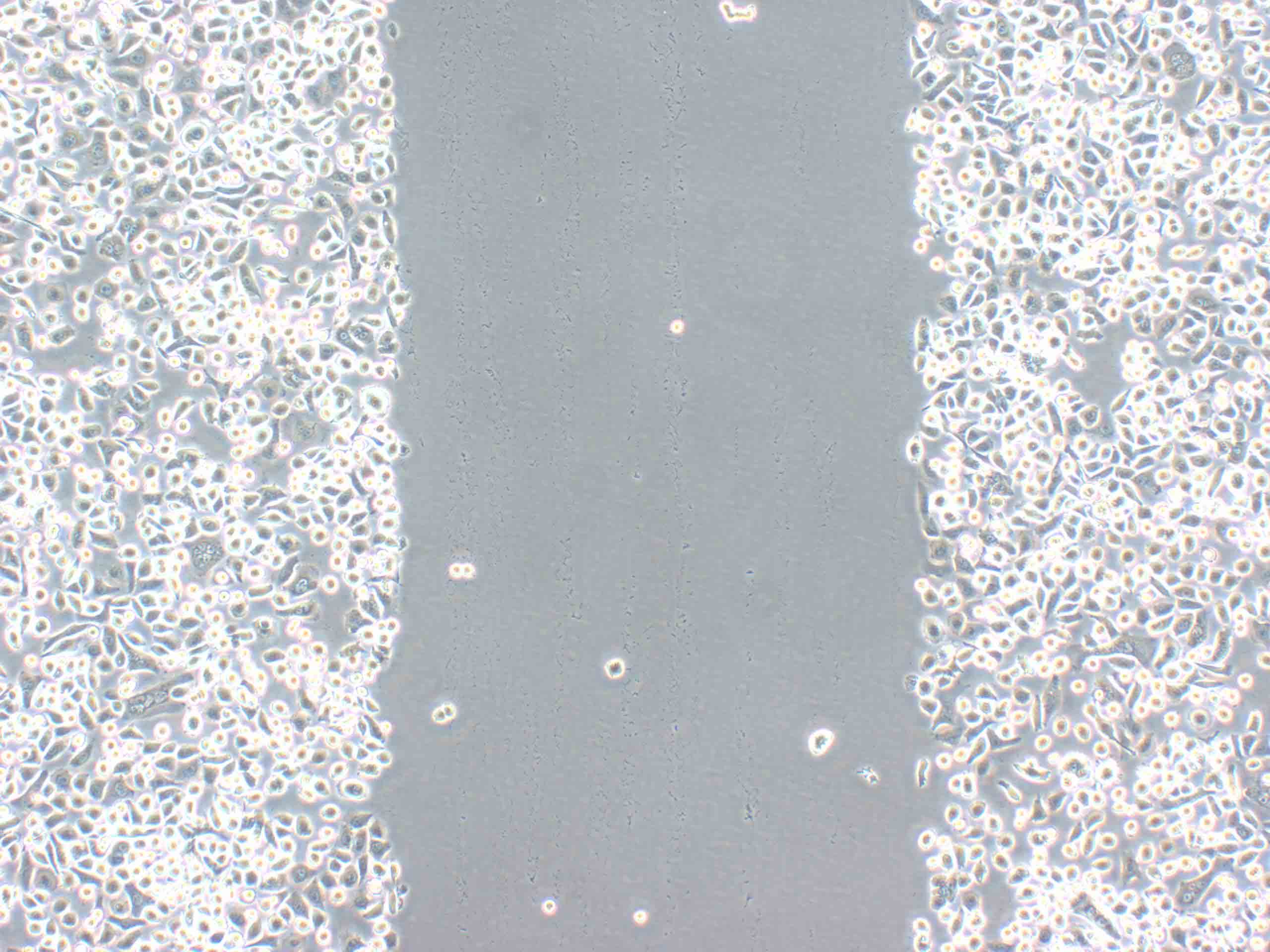

Supplement: Supplemental Information 1 [file peerj-08-8910-s001.zip › scratching_assay/acpc-1/2/Normal-0h.jpg]

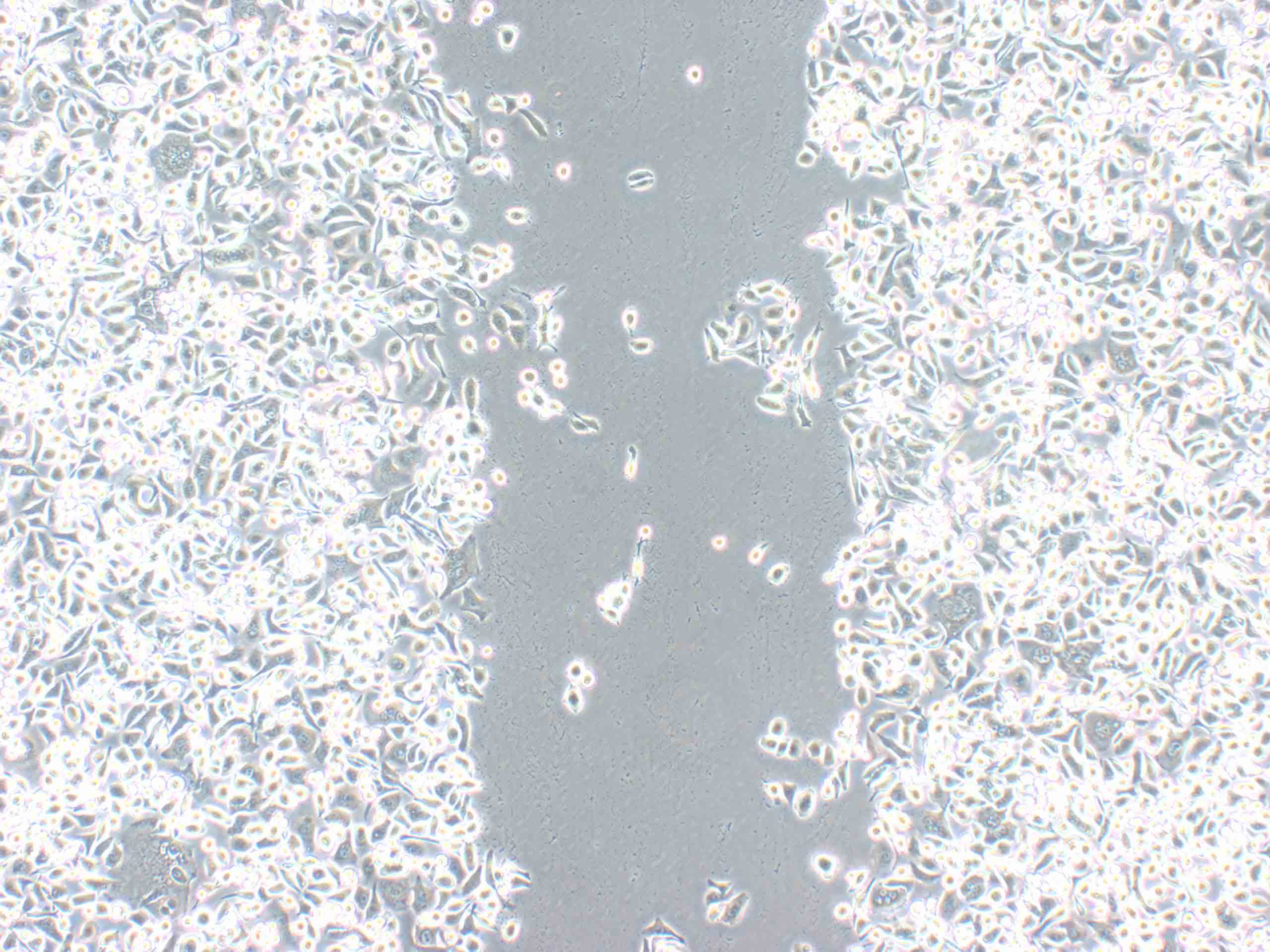

Supplement: Supplemental Information 1 [file peerj-08-8910-s001.zip › scratching_assay/acpc-1/2/Normal-12h.jpg]

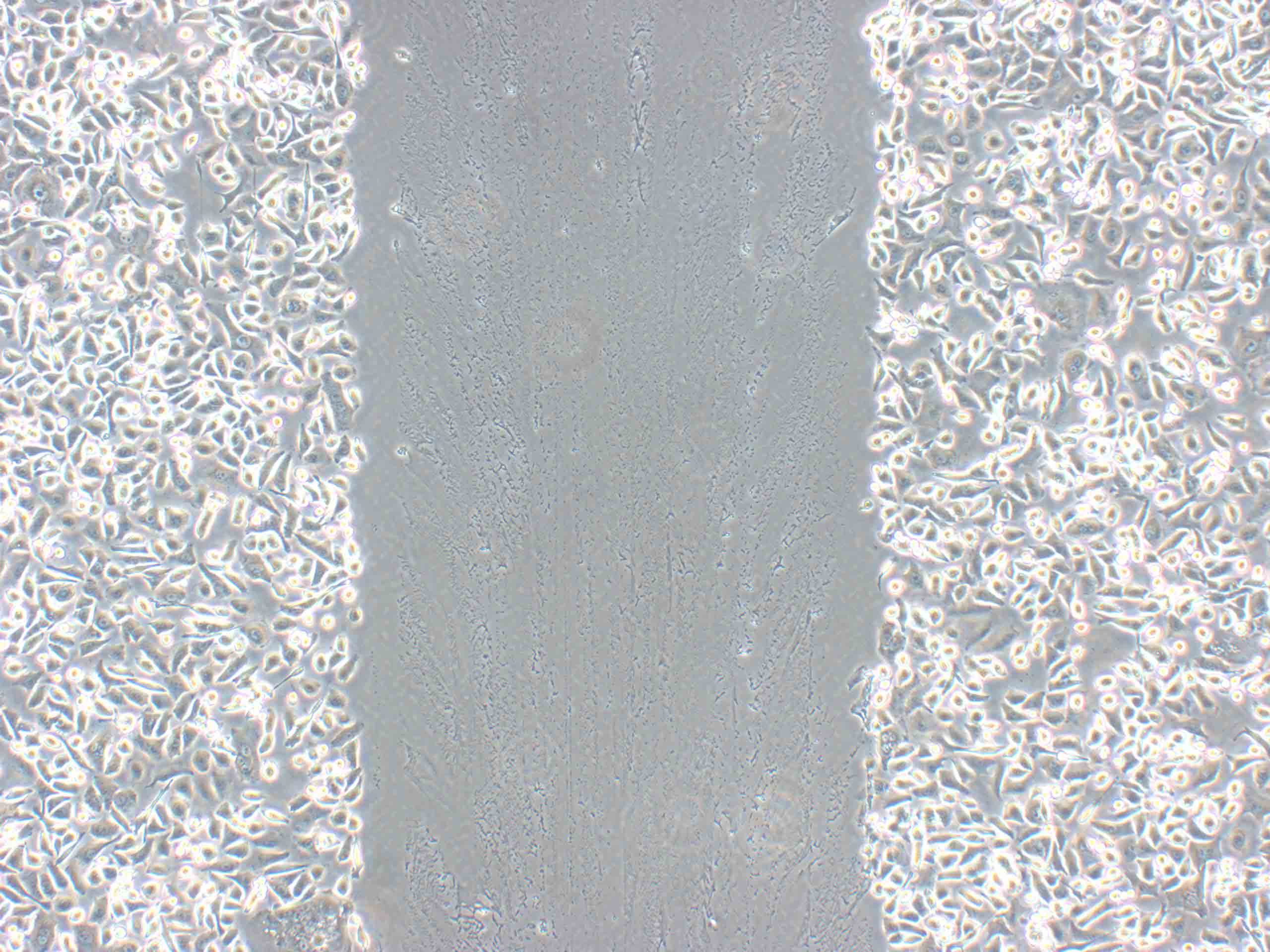

Supplement: Supplemental Information 1 [file peerj-08-8910-s001.zip › scratching_assay/acpc-1/2/si-0h.jpg]

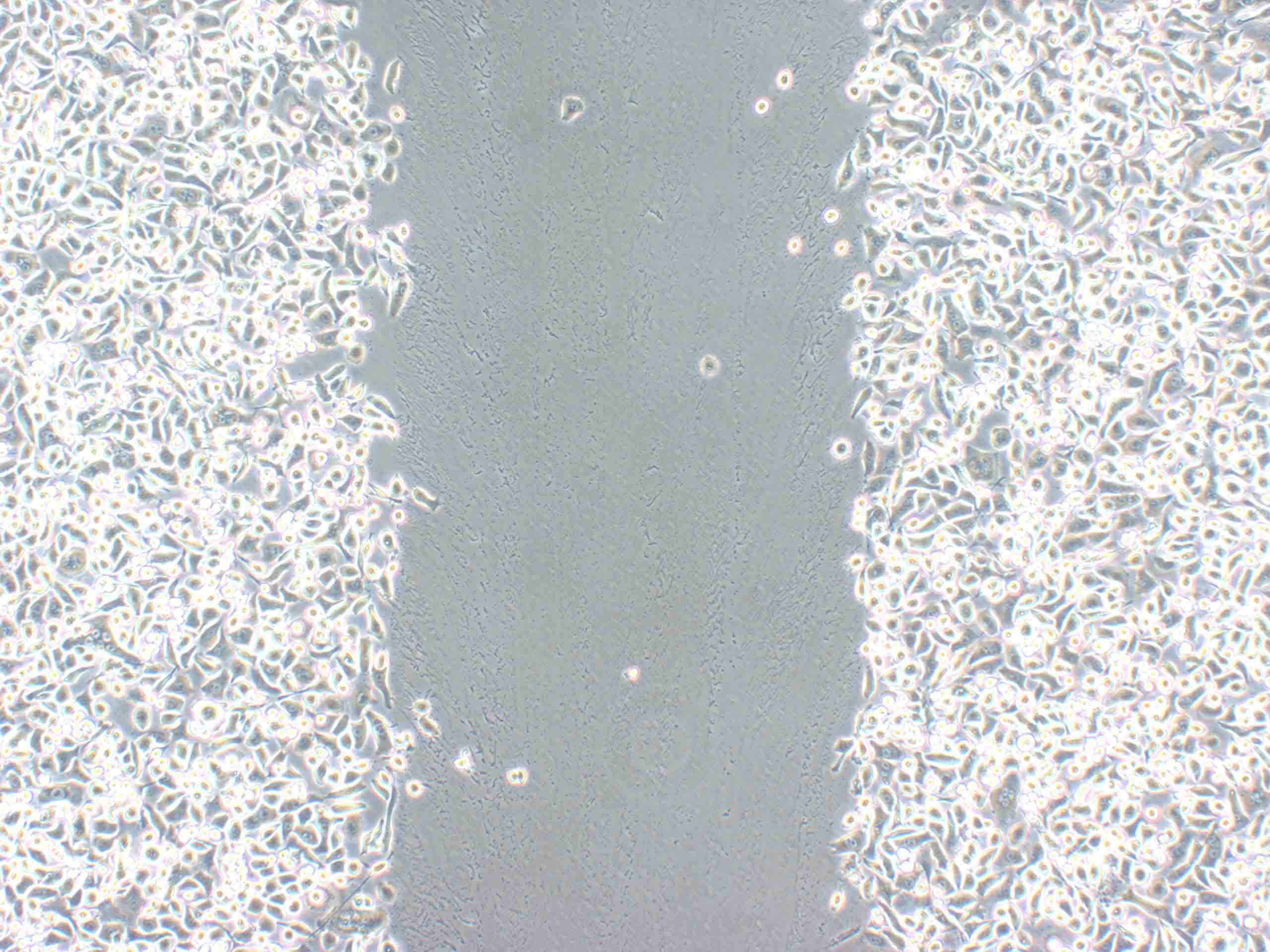

Supplement: Supplemental Information 1 [file peerj-08-8910-s001.zip › scratching_assay/acpc-1/2/si-12h.jpg]

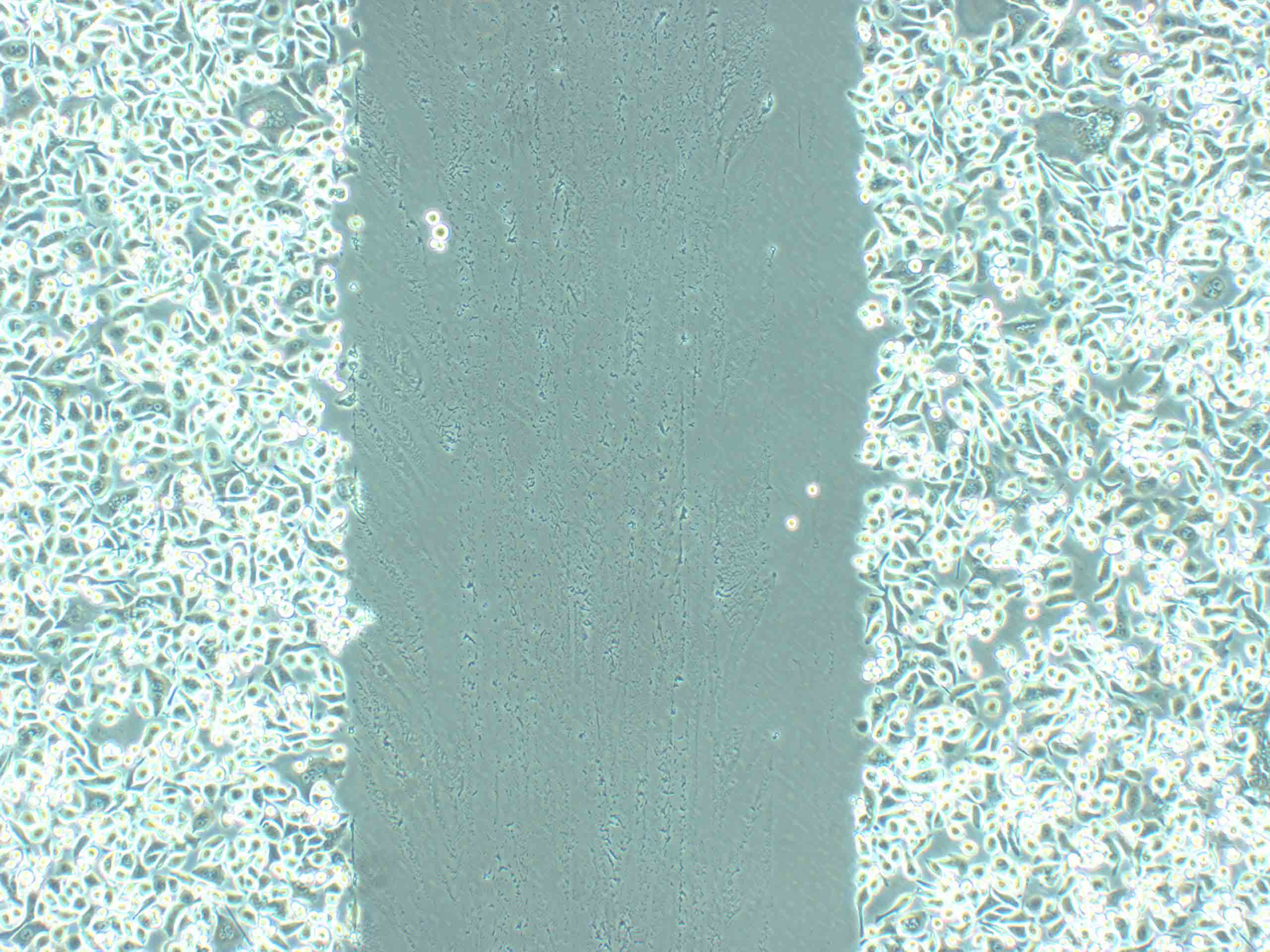

Supplement: Supplemental Information 1 [file peerj-08-8910-s001.zip › scratching_assay/acpc-1/3/Control-0h.jpg]

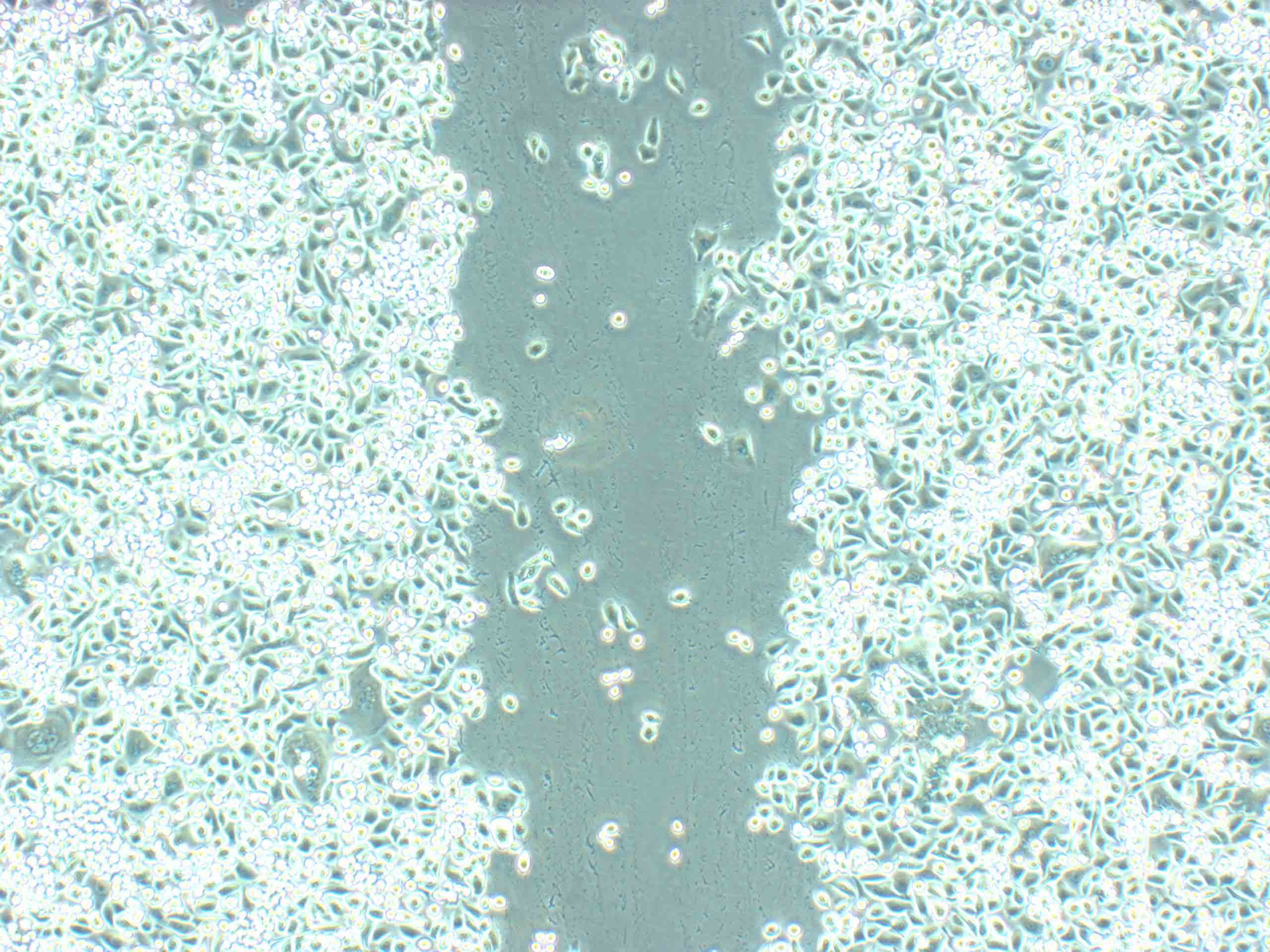

Supplement: Supplemental Information 1 [file peerj-08-8910-s001.zip › scratching_assay/acpc-1/3/Control-12h.jpg]

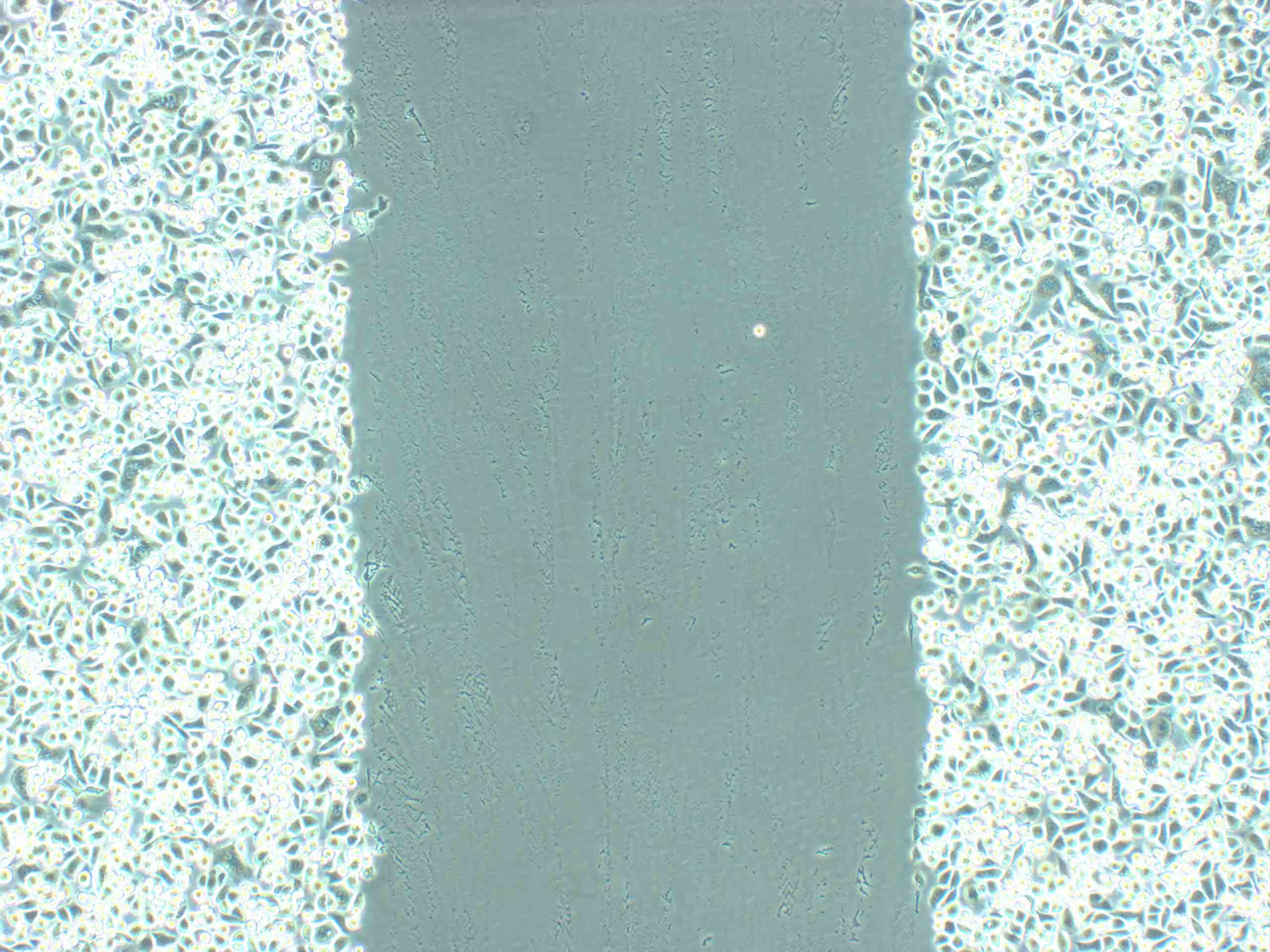

Supplement: Supplemental Information 1 [file peerj-08-8910-s001.zip › scratching_assay/acpc-1/3/Normal-0h.jpg]

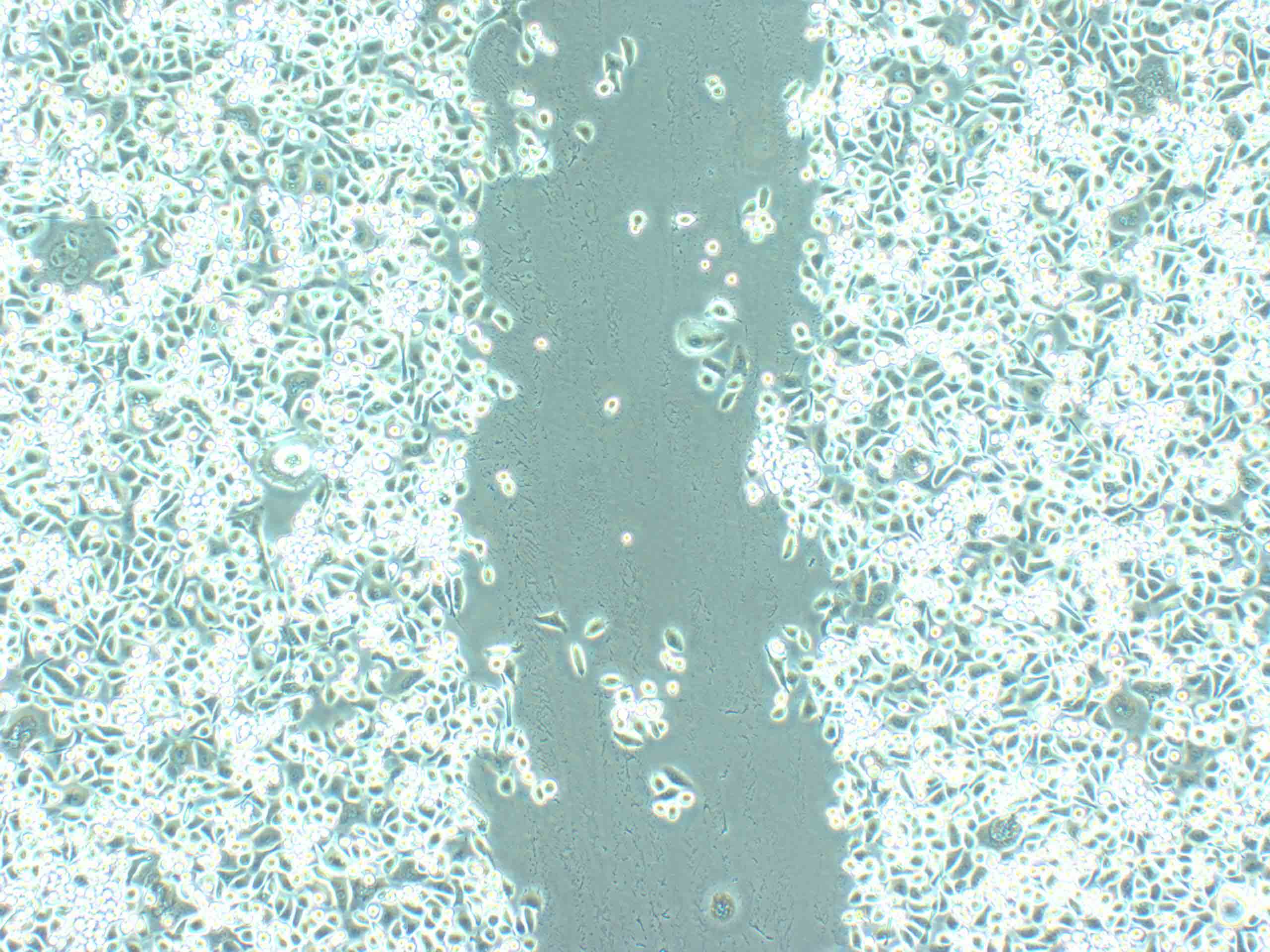

Supplement: Supplemental Information 1 [file peerj-08-8910-s001.zip › scratching_assay/acpc-1/3/Normal-12h.jpg]

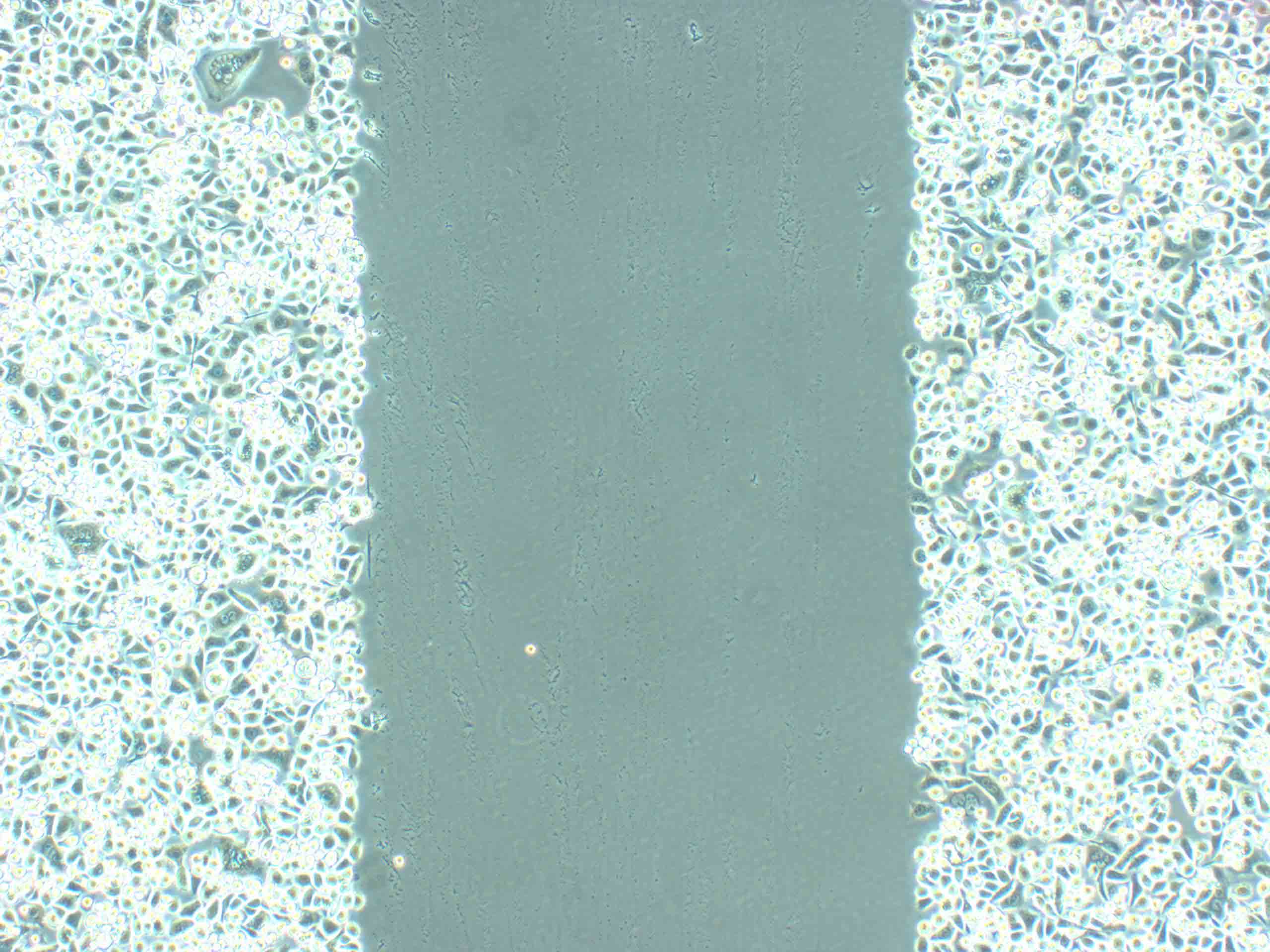

Supplement: Supplemental Information 1 [file peerj-08-8910-s001.zip › scratching_assay/acpc-1/3/si-0h.jpg]

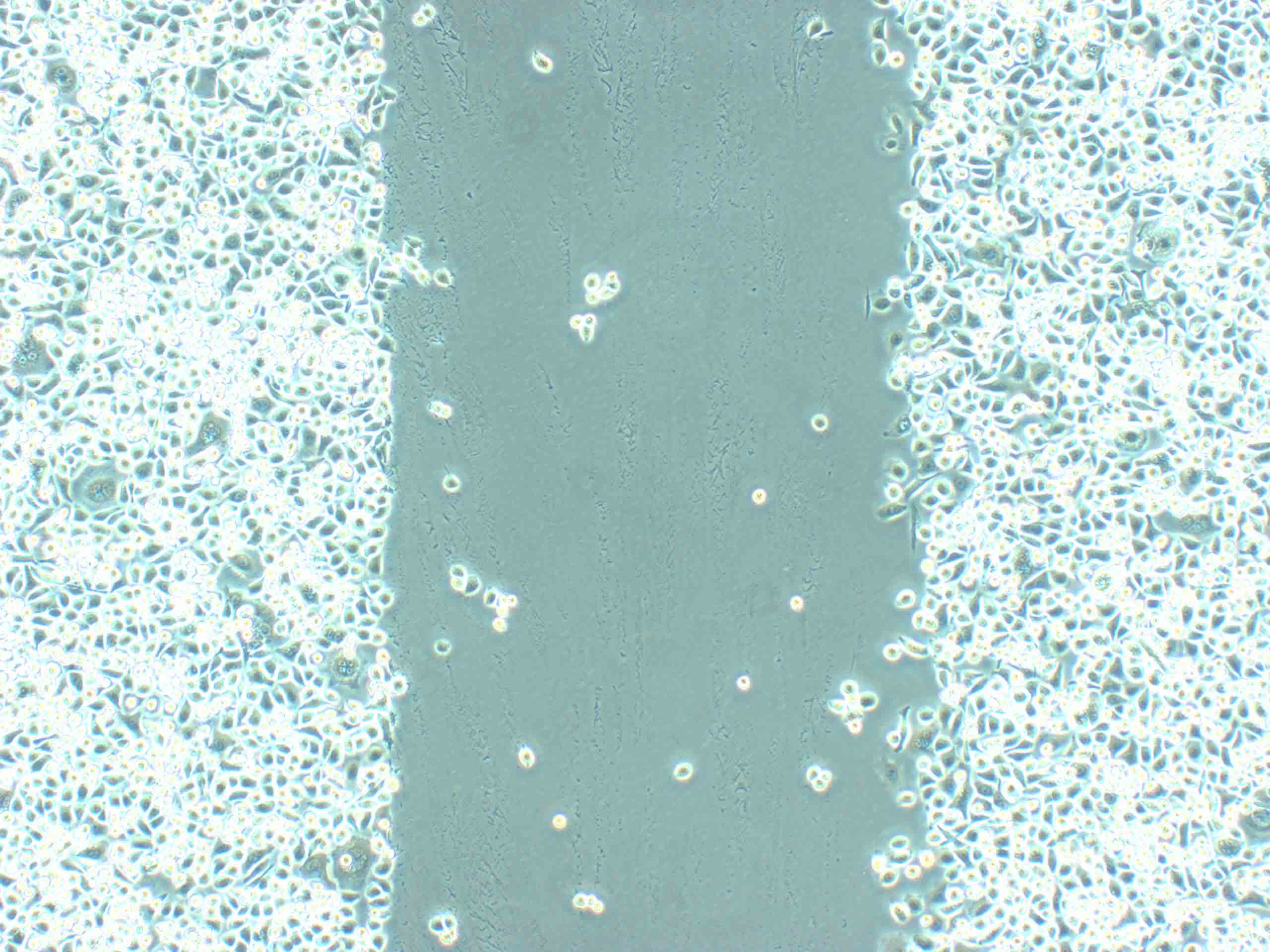

Supplement: Supplemental Information 1 [file peerj-08-8910-s001.zip › scratching_assay/acpc-1/3/si-12h.jpg]

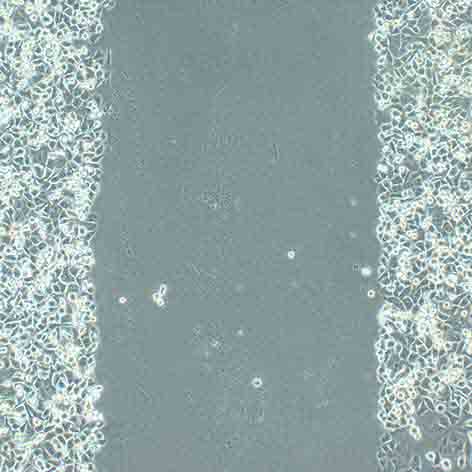

Supplement: Supplemental Information 1 [file peerj-08-8910-s001.zip › scratching_assay/panc-1/1/panc--0h--control.jpg]

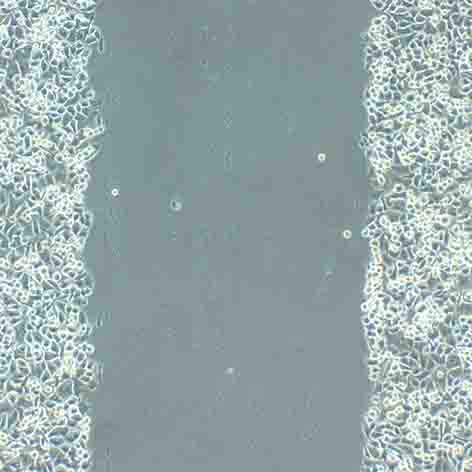

Supplement: Supplemental Information 1 [file peerj-08-8910-s001.zip › scratching_assay/panc-1/1/panc--0h--normal.jpg]

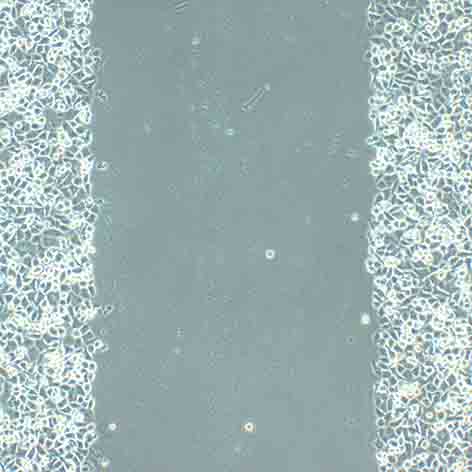

Supplement: Supplemental Information 1 [file peerj-08-8910-s001.zip › scratching_assay/panc-1/1/panc--0h--si.jpg]

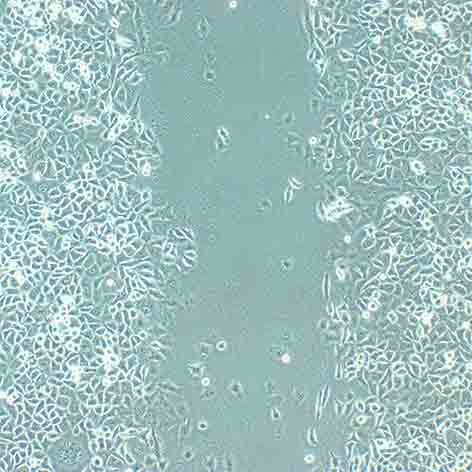

Supplement: Supplemental Information 1 [file peerj-08-8910-s001.zip › scratching_assay/panc-1/1/panc--12h--control.jpg]

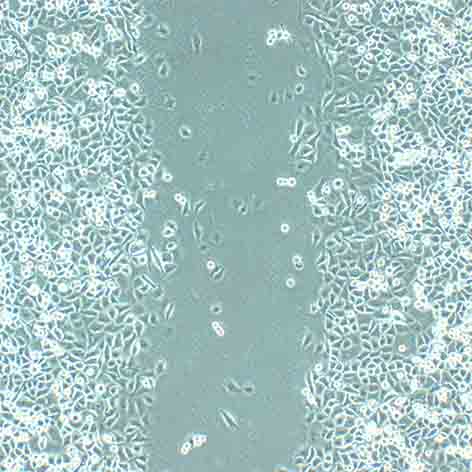

Supplement: Supplemental Information 1 [file peerj-08-8910-s001.zip › scratching_assay/panc-1/1/panc--12h--normal.jpg]

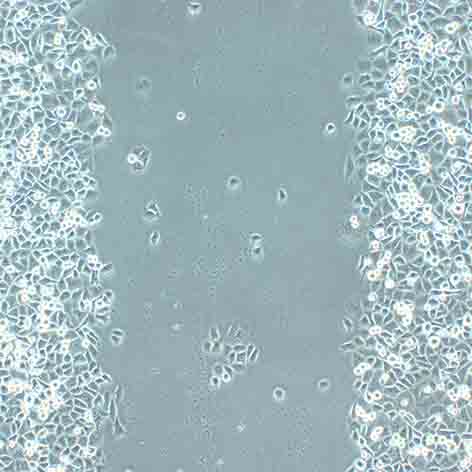

Supplement: Supplemental Information 1 [file peerj-08-8910-s001.zip › scratching_assay/panc-1/1/panc--12h--si.jpg]

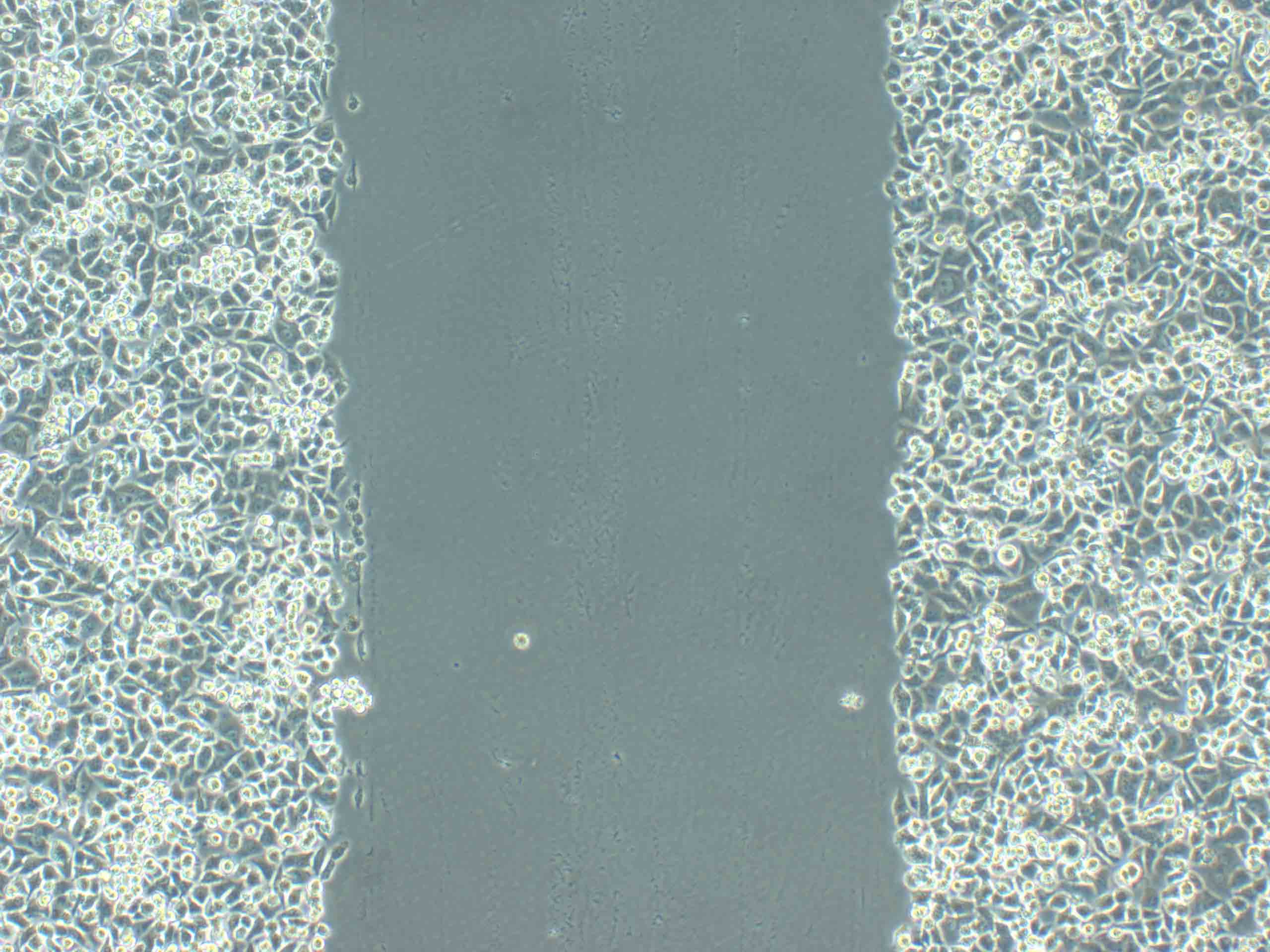

Supplement: Supplemental Information 1 [file peerj-08-8910-s001.zip › scratching_assay/panc-1/2/Control-0h.jpg]

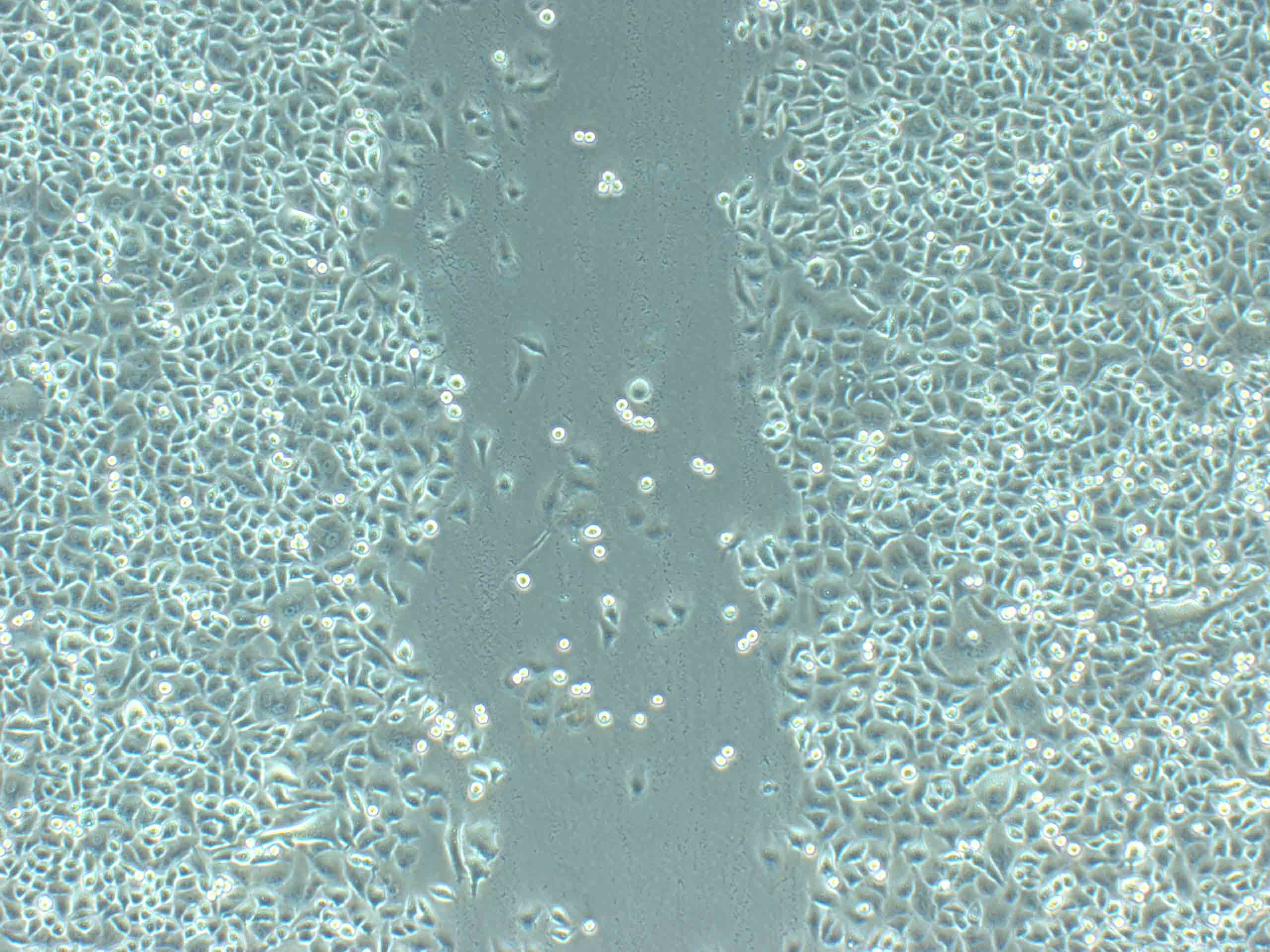

Supplement: Supplemental Information 1 [file peerj-08-8910-s001.zip › scratching_assay/panc-1/2/Control-12h.jpg]

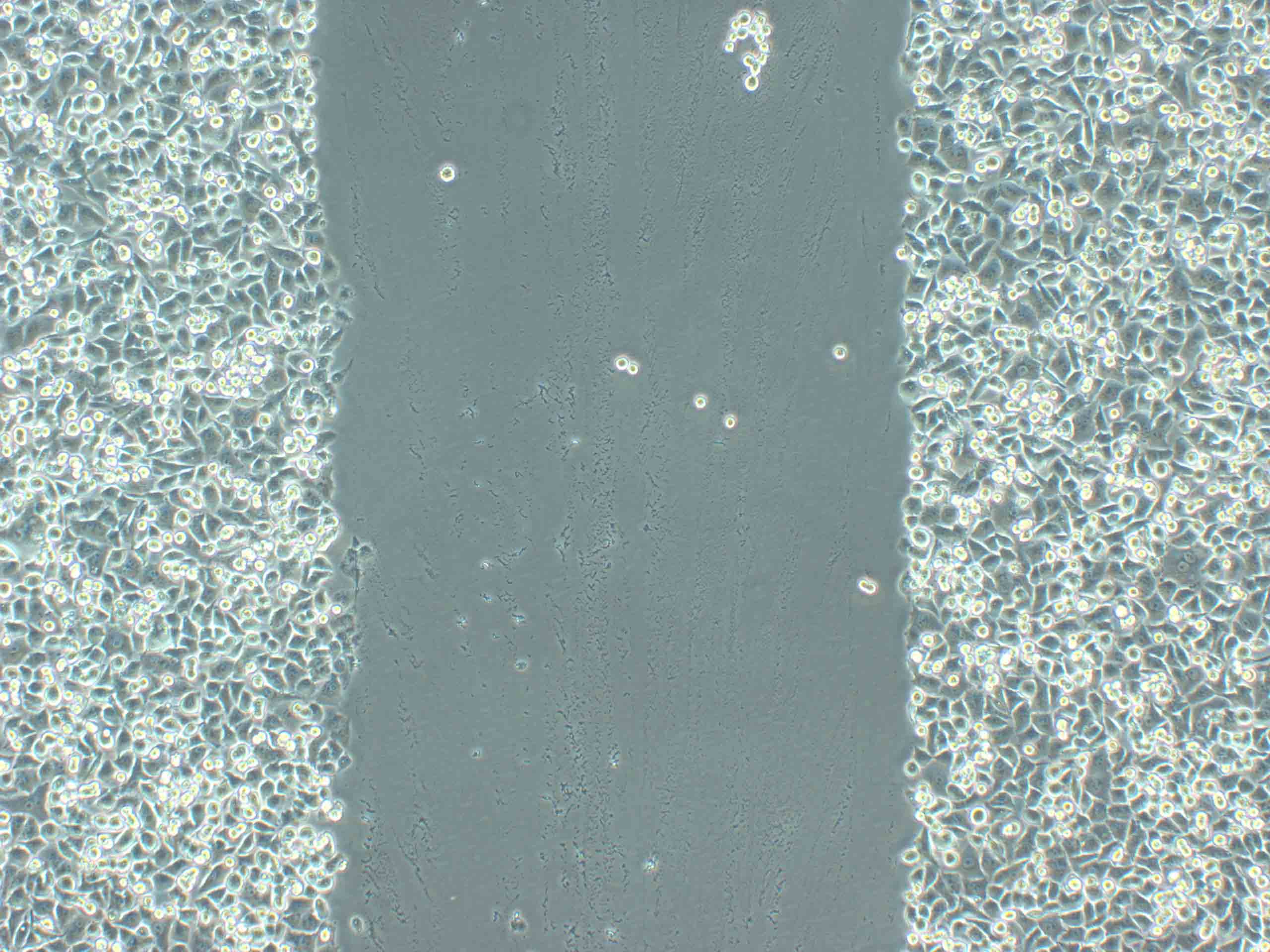

Supplement: Supplemental Information 1 [file peerj-08-8910-s001.zip › scratching_assay/panc-1/2/Normal-0h.jpg]

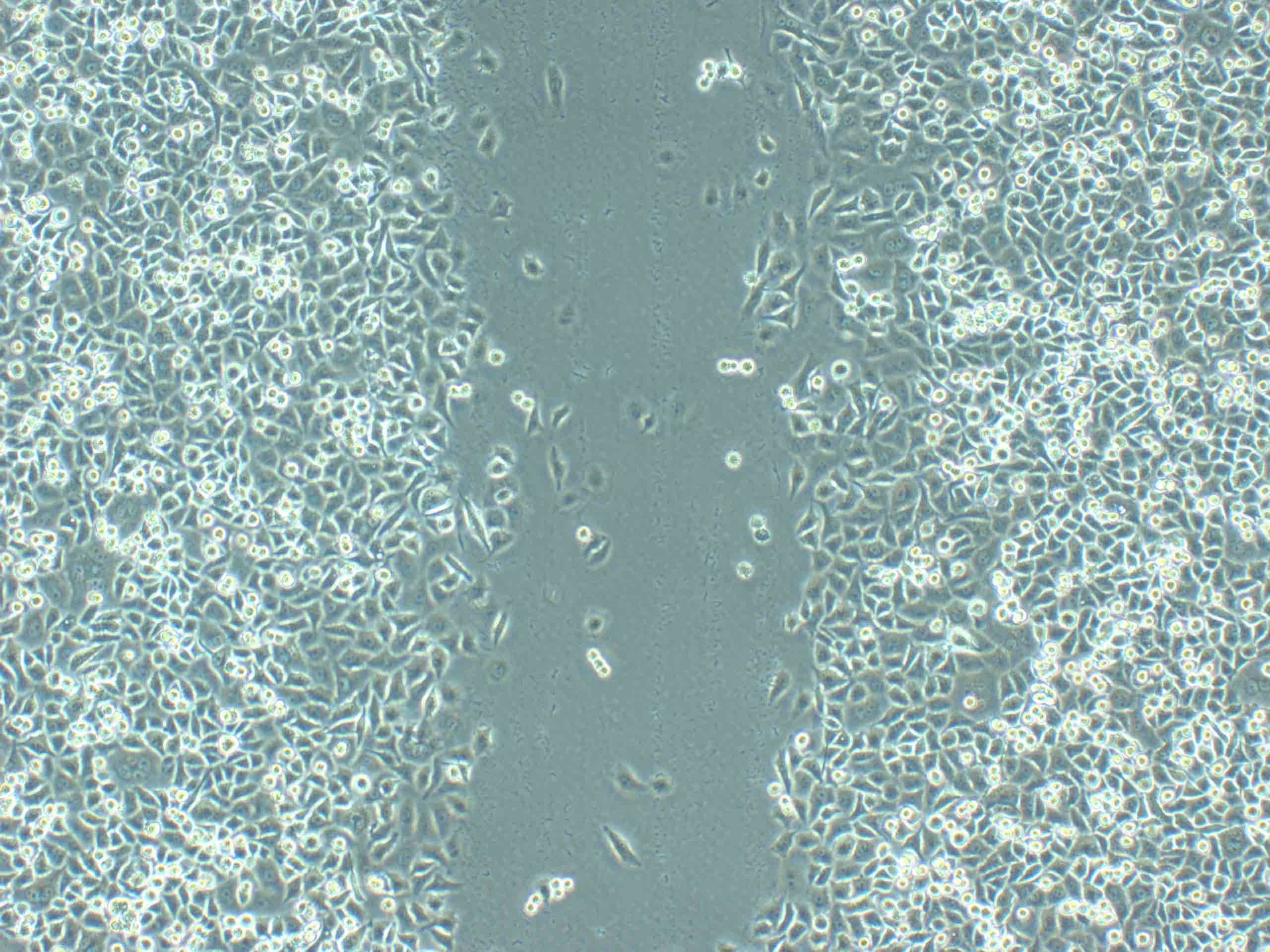

Supplement: Supplemental Information 1 [file peerj-08-8910-s001.zip › scratching_assay/panc-1/2/Normal-12h.jpg]

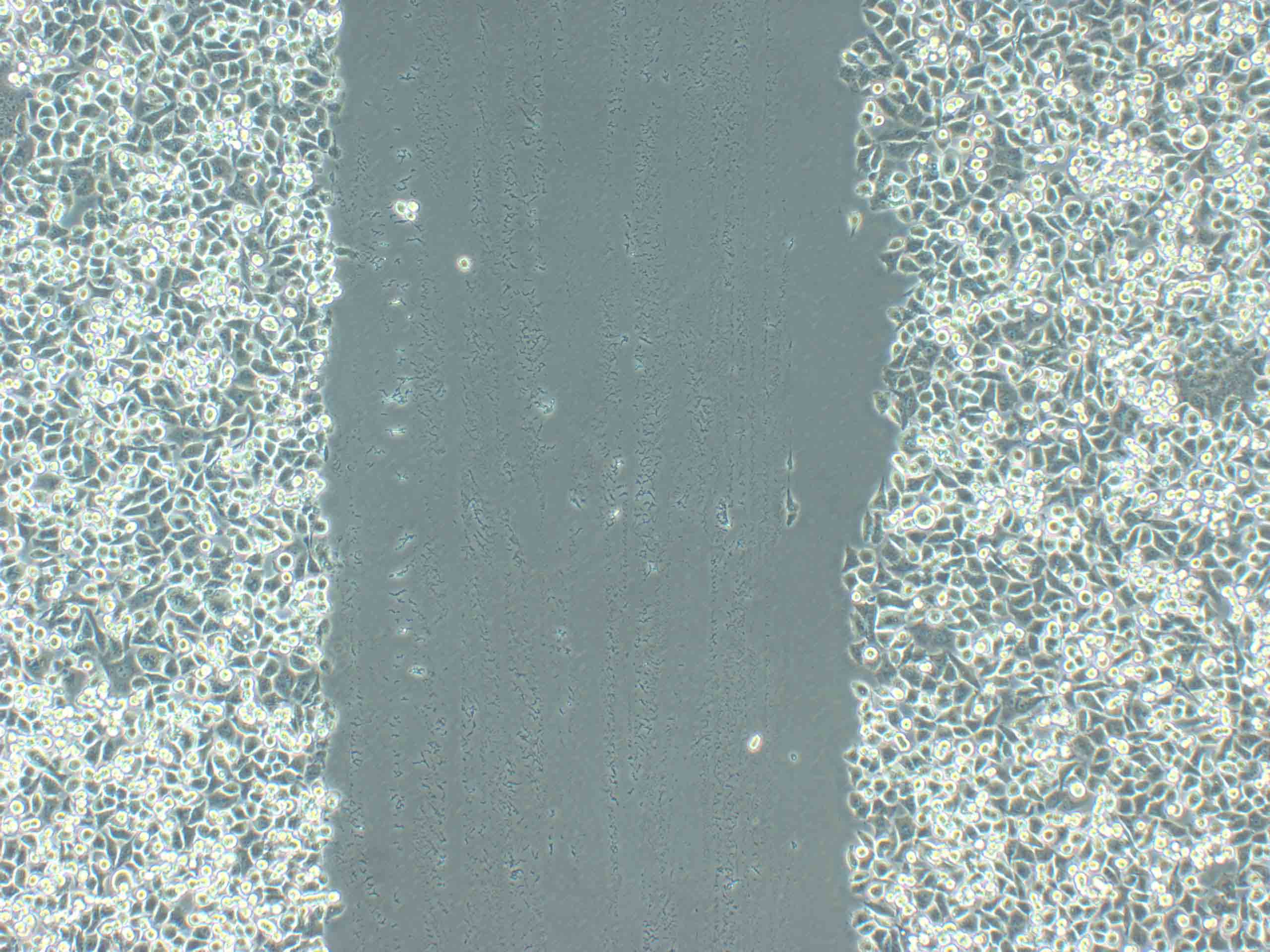

Supplement: Supplemental Information 1 [file peerj-08-8910-s001.zip › scratching_assay/panc-1/2/Si-0h.jpg]

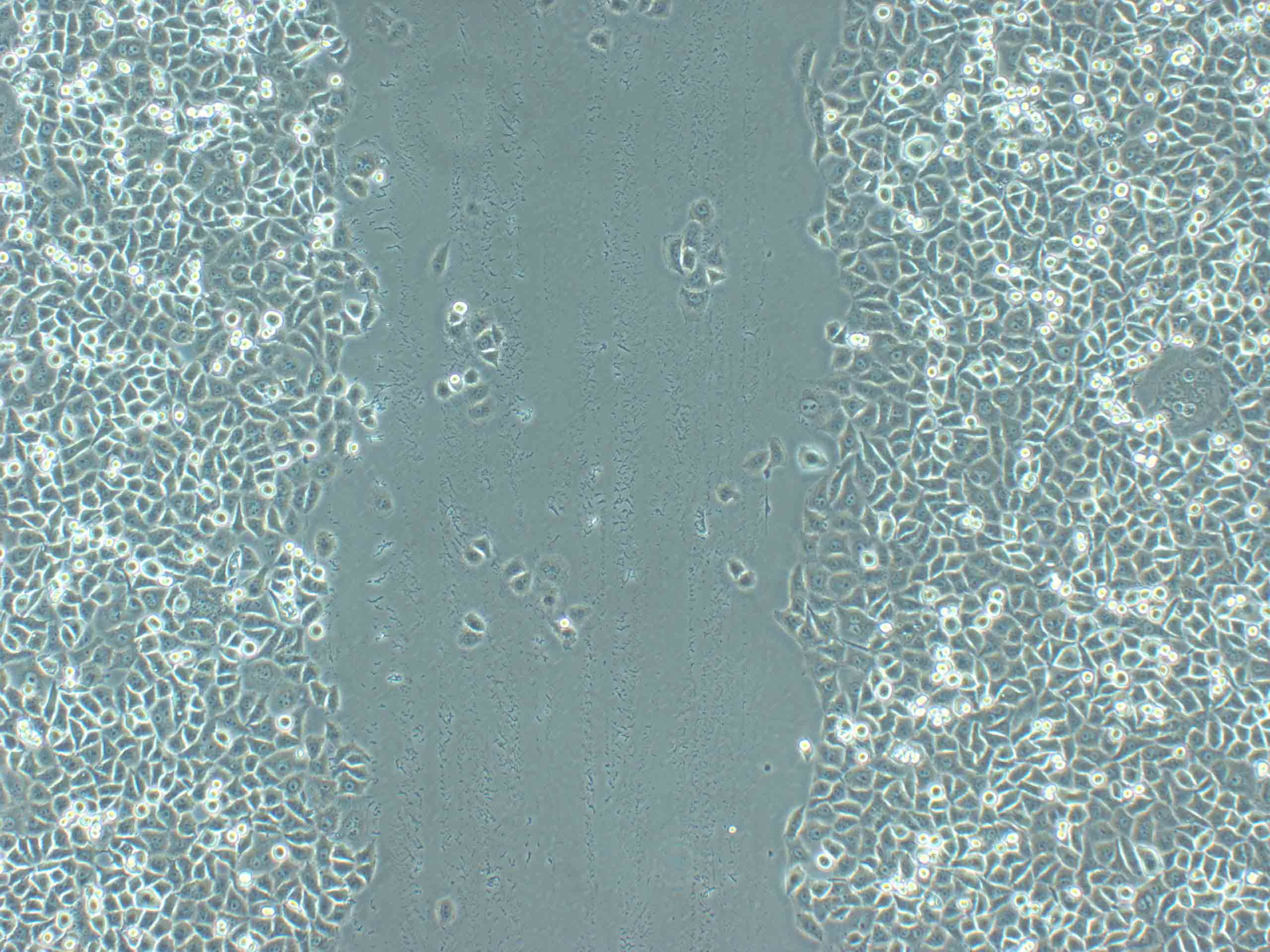

Supplement: Supplemental Information 1 [file peerj-08-8910-s001.zip › scratching_assay/panc-1/2/Si-12h.jpg]

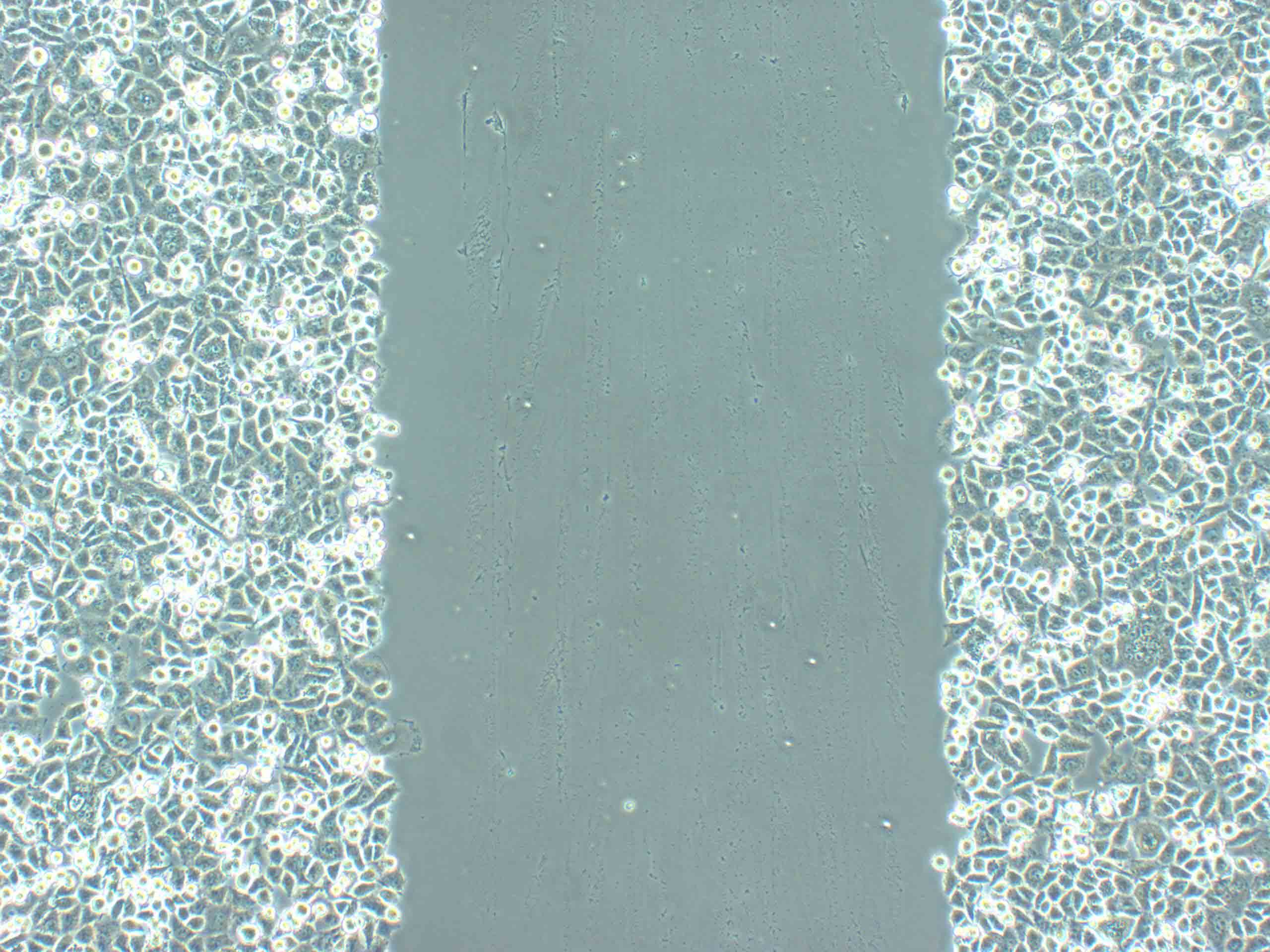

Supplement: Supplemental Information 1 [file peerj-08-8910-s001.zip › scratching_assay/panc-1/3/Control-0h.jpg]

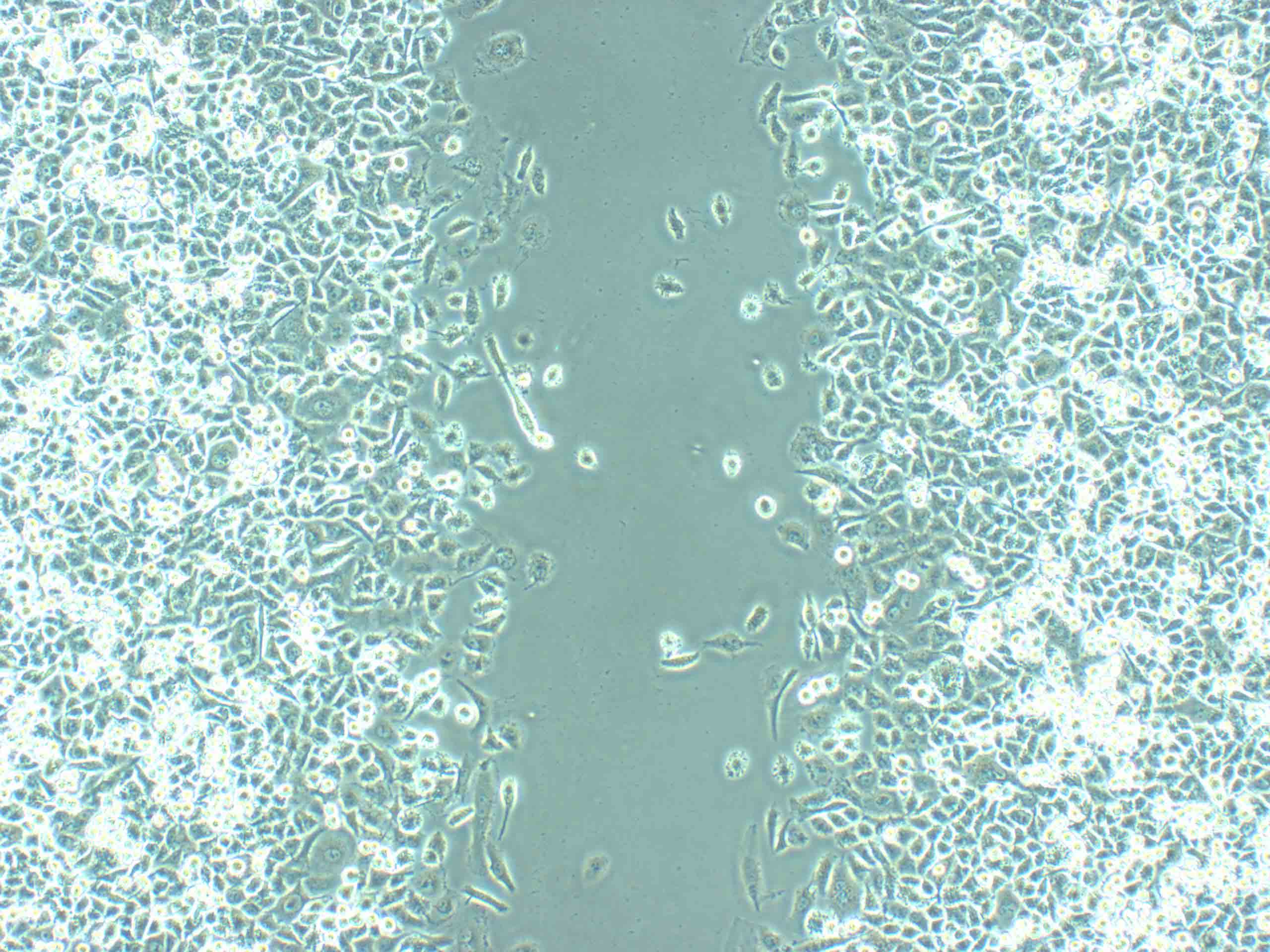

Supplement: Supplemental Information 1 [file peerj-08-8910-s001.zip › scratching_assay/panc-1/3/Control-12h .jpg]

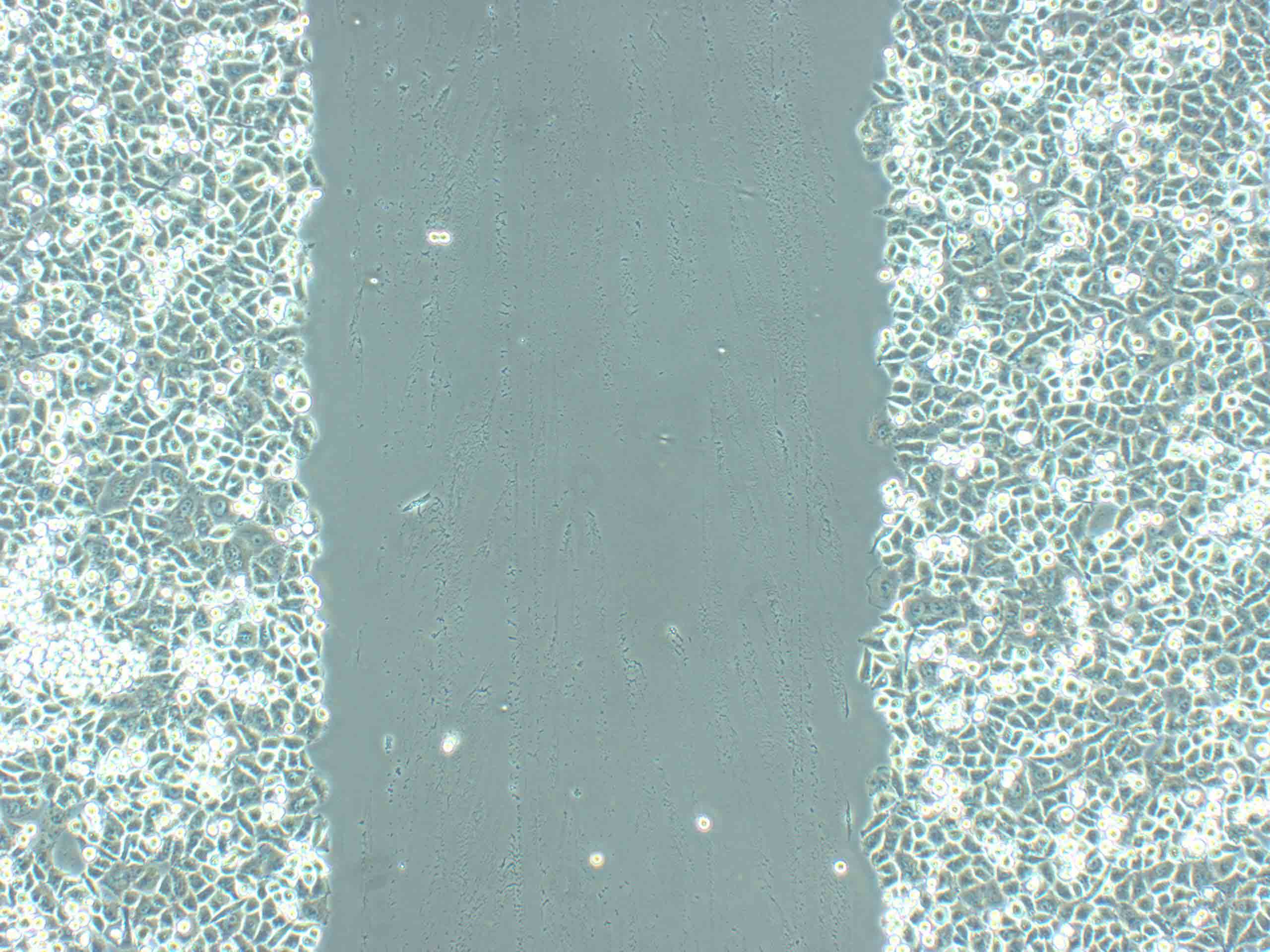

Supplement: Supplemental Information 1 [file peerj-08-8910-s001.zip › scratching_assay/panc-1/3/Normal-0h.jpg]

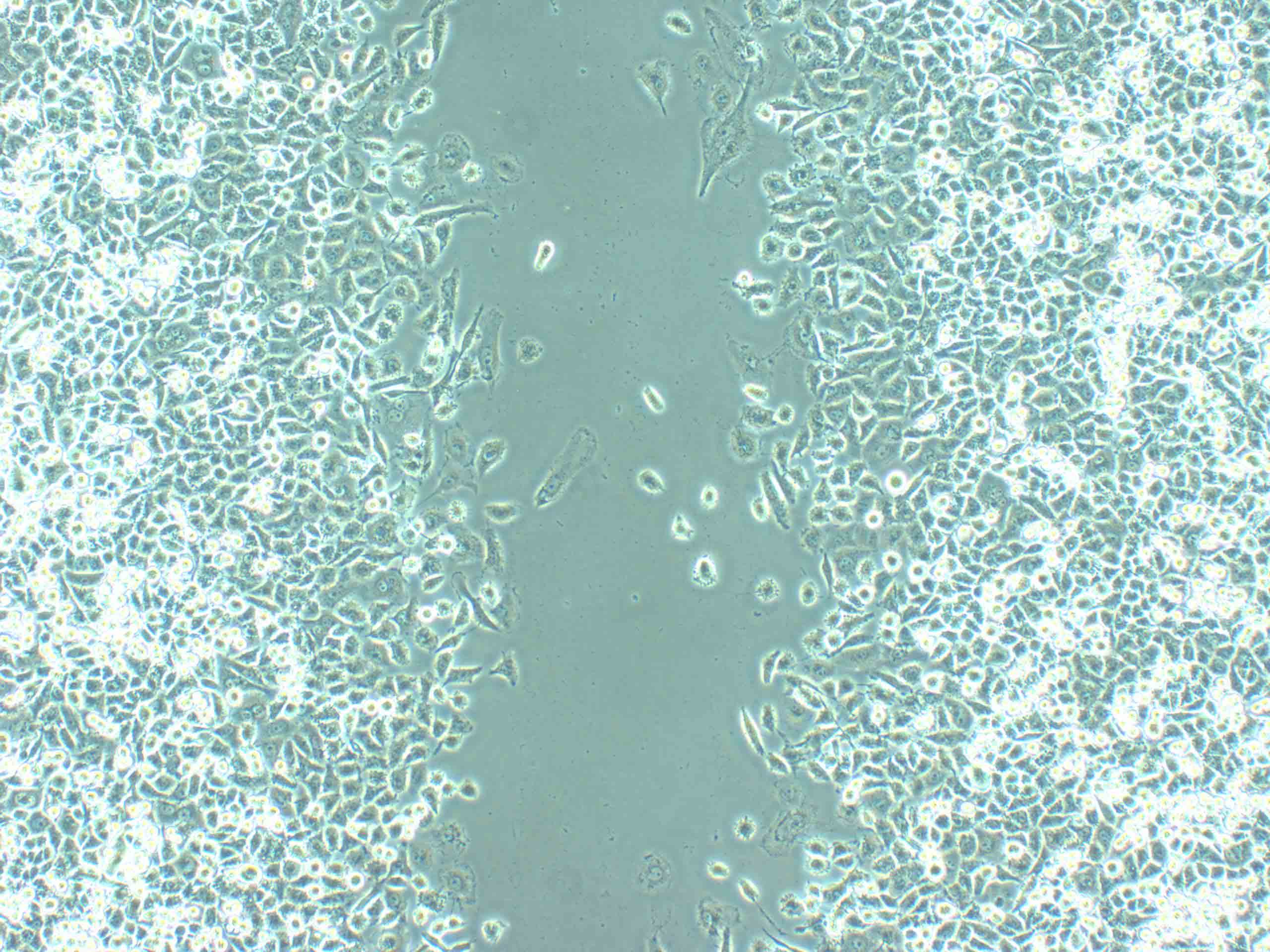

Supplement: Supplemental Information 1 [file peerj-08-8910-s001.zip › scratching_assay/panc-1/3/Normal-12h.jpg]

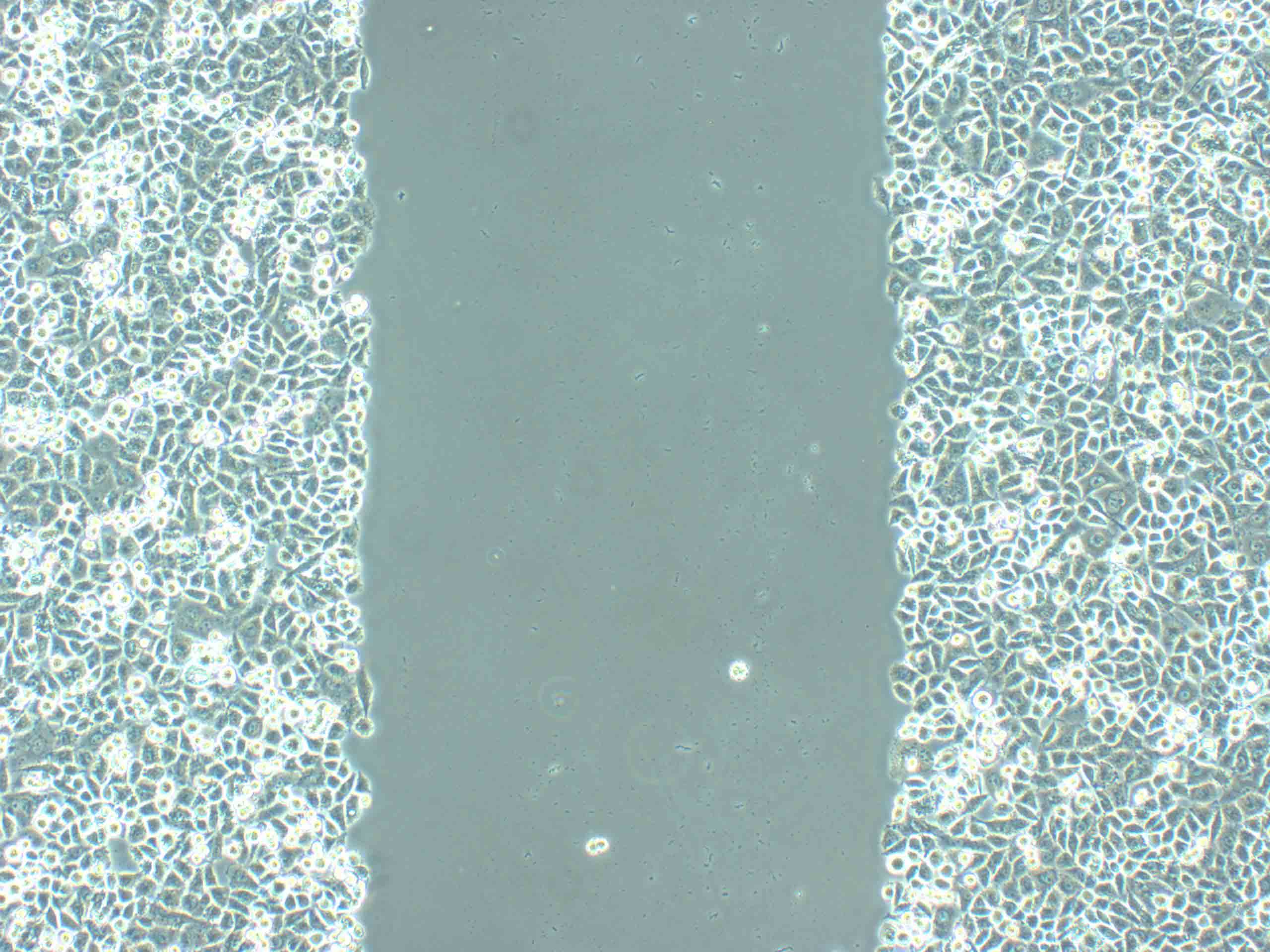

Supplement: Supplemental Information 1 [file peerj-08-8910-s001.zip › scratching_assay/panc-1/3/Si-0h.jpg]

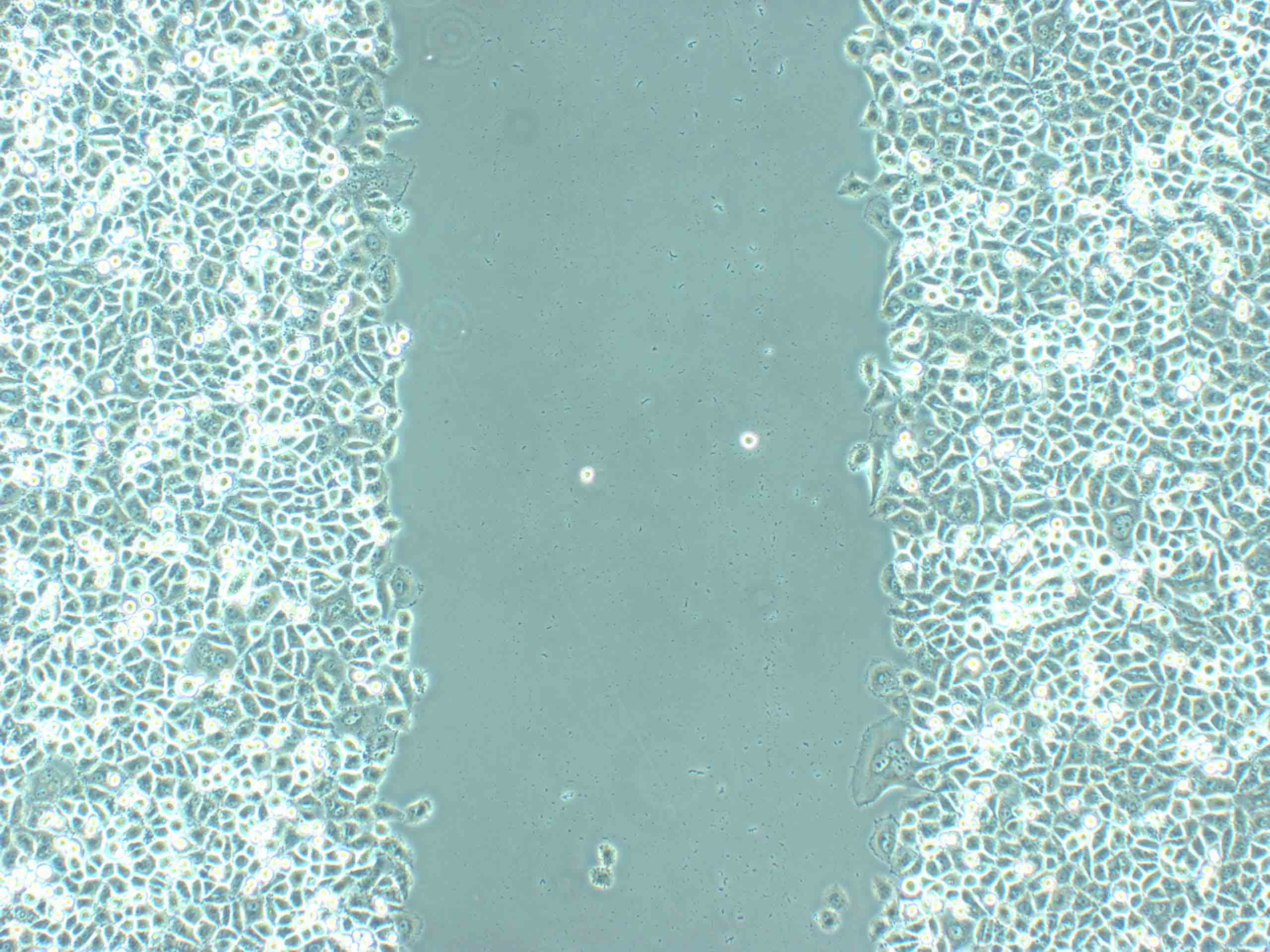

Supplement: Supplemental Information 1 [file peerj-08-8910-s001.zip › scratching_assay/panc-1/3/Si-12h.jpg]

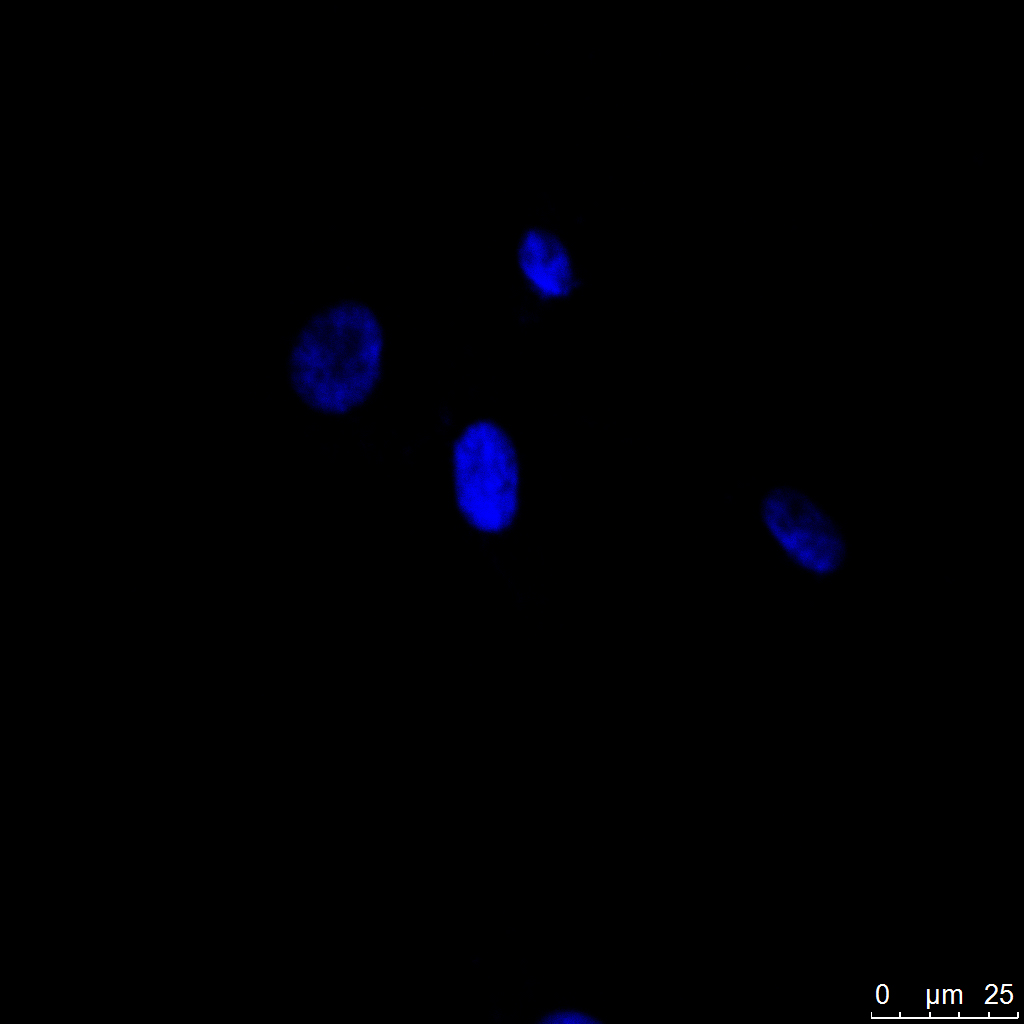

Supplement: Figure S2 — To further investigate interaction networks involving ELMO2 and Gαi2, immunofluorescence microscopy was used to examine the subcellular localization of the two proteins. [file peerj-08-8910-s002.zip › Immunofluorescence/20190715_Series013_z0_ch00.jpg]

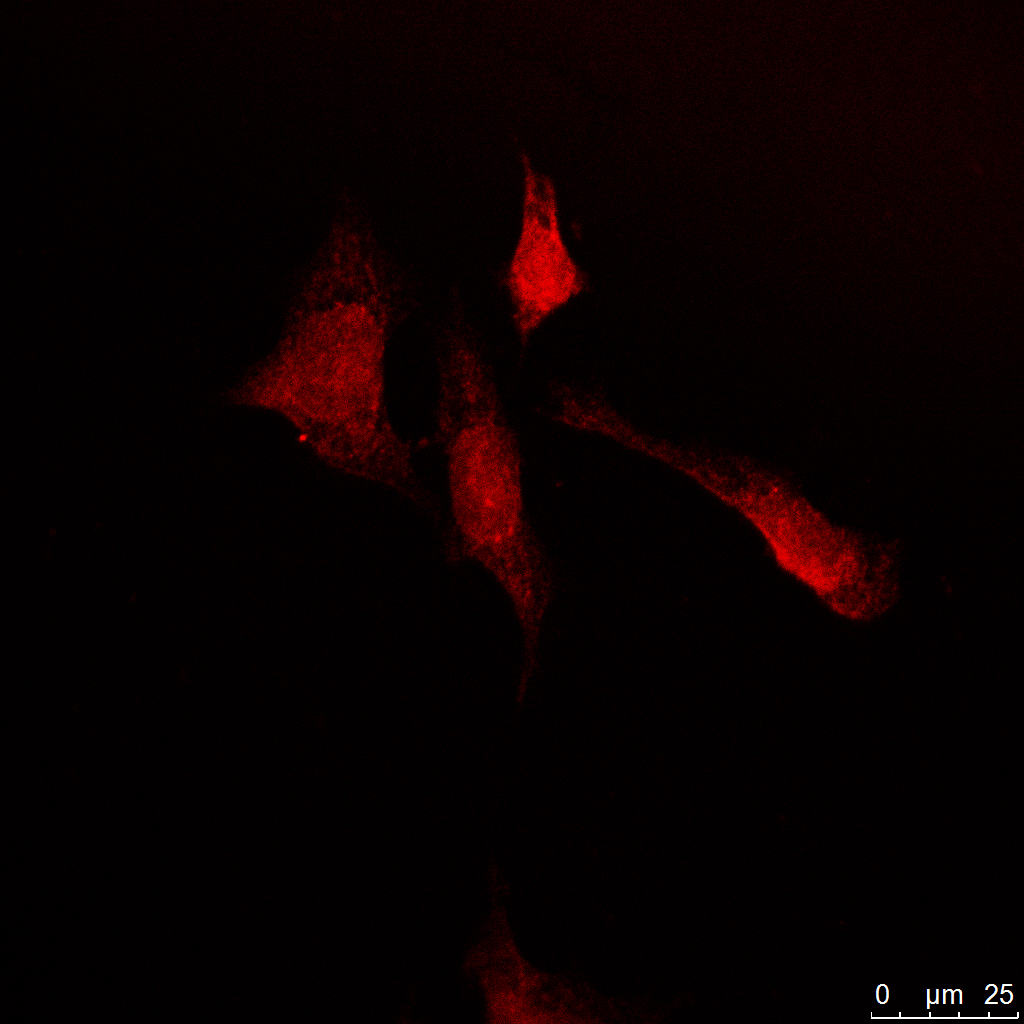

Supplement: Figure S2 — To further investigate interaction networks involving ELMO2 and Gαi2, immunofluorescence microscopy was used to examine the subcellular localization of the two proteins. [file peerj-08-8910-s002.zip › Immunofluorescence/20190715_Series013_z0_ch02.jpg]

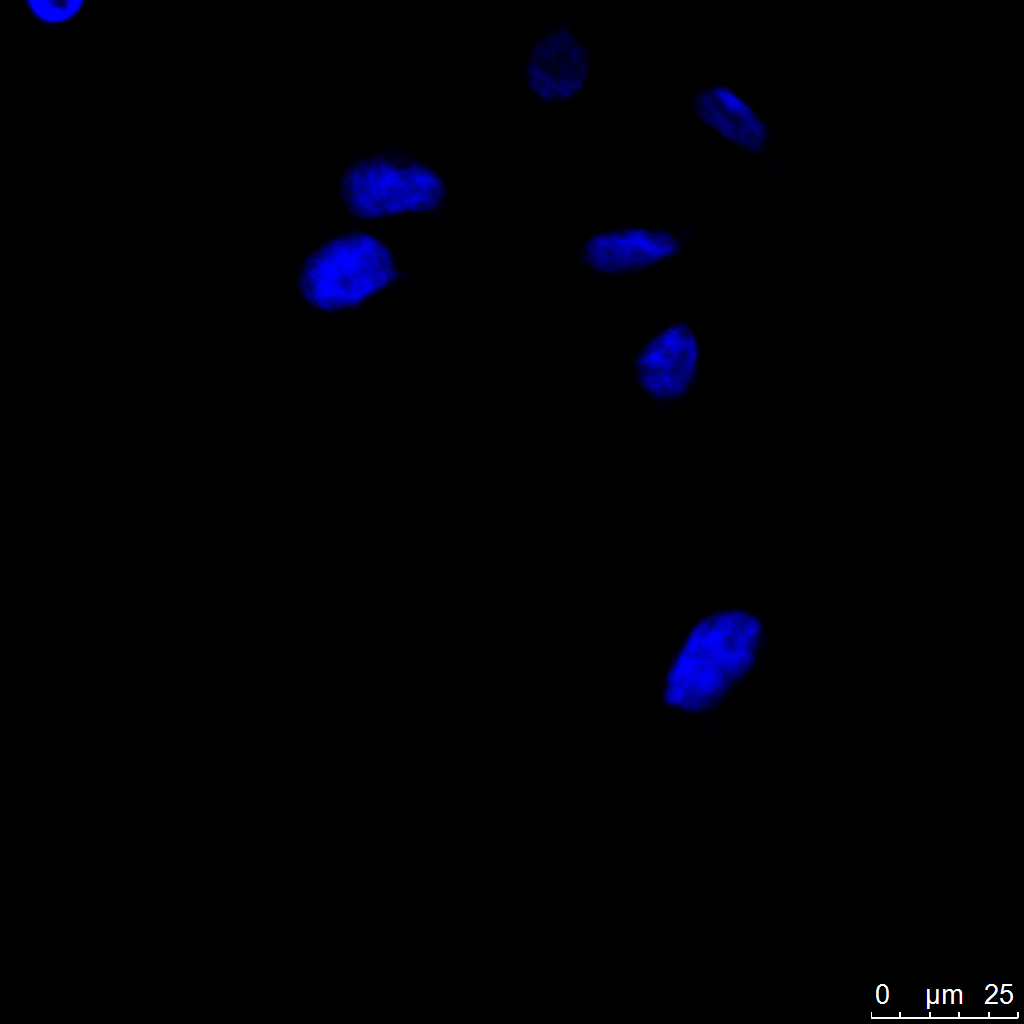

Supplement: Figure S2 — To further investigate interaction networks involving ELMO2 and Gαi2, immunofluorescence microscopy was used to examine the subcellular localization of the two proteins. [file peerj-08-8910-s002.zip › Immunofluorescence/20190715_Series021_z0_ch00.jpg]

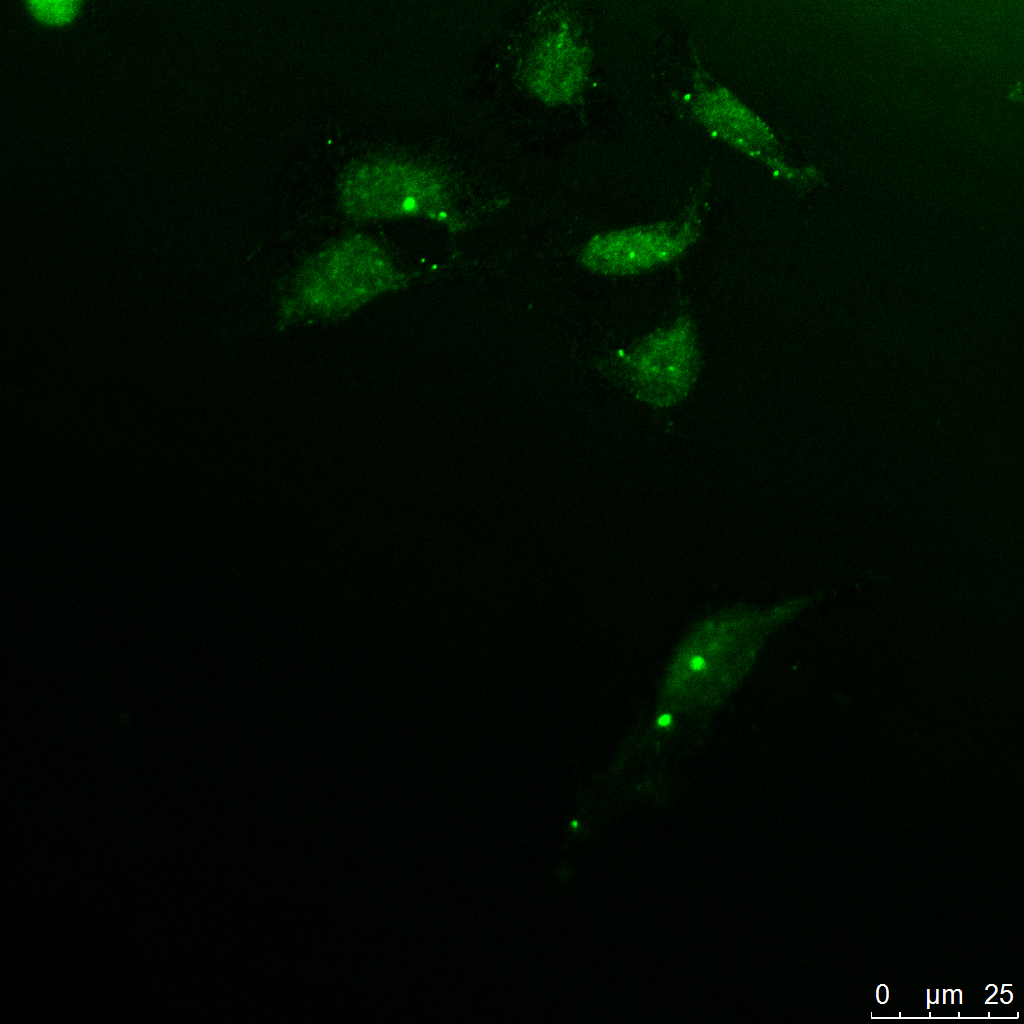

Supplement: Figure S2 — To further investigate interaction networks involving ELMO2 and Gαi2, immunofluorescence microscopy was used to examine the subcellular localization of the two proteins. [file peerj-08-8910-s002.zip › Immunofluorescence/20190715_Series021_z0_ch01.jpg]

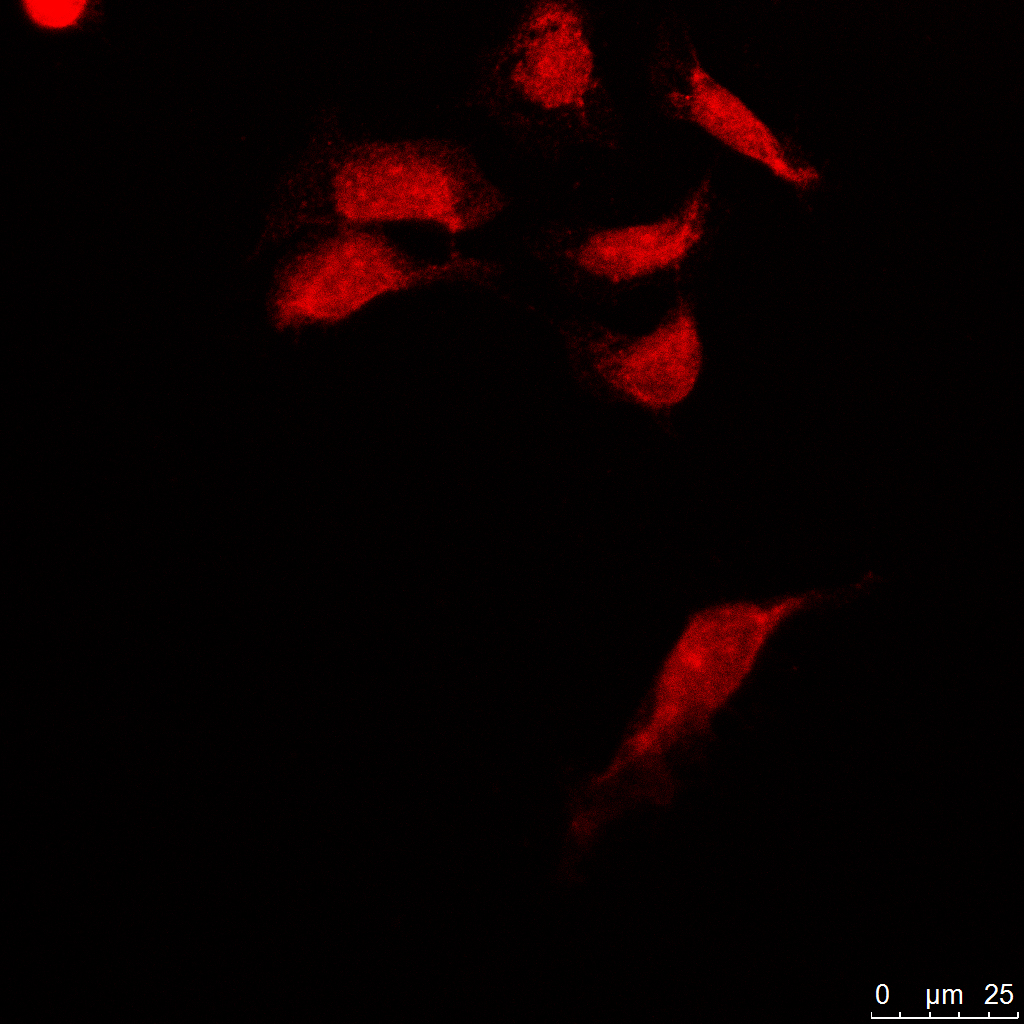

Supplement: Figure S2 — To further investigate interaction networks involving ELMO2 and Gαi2, immunofluorescence microscopy was used to examine the subcellular localization of the two proteins. [file peerj-08-8910-s002.zip › Immunofluorescence/20190715_Series021_z0_ch02.jpg]

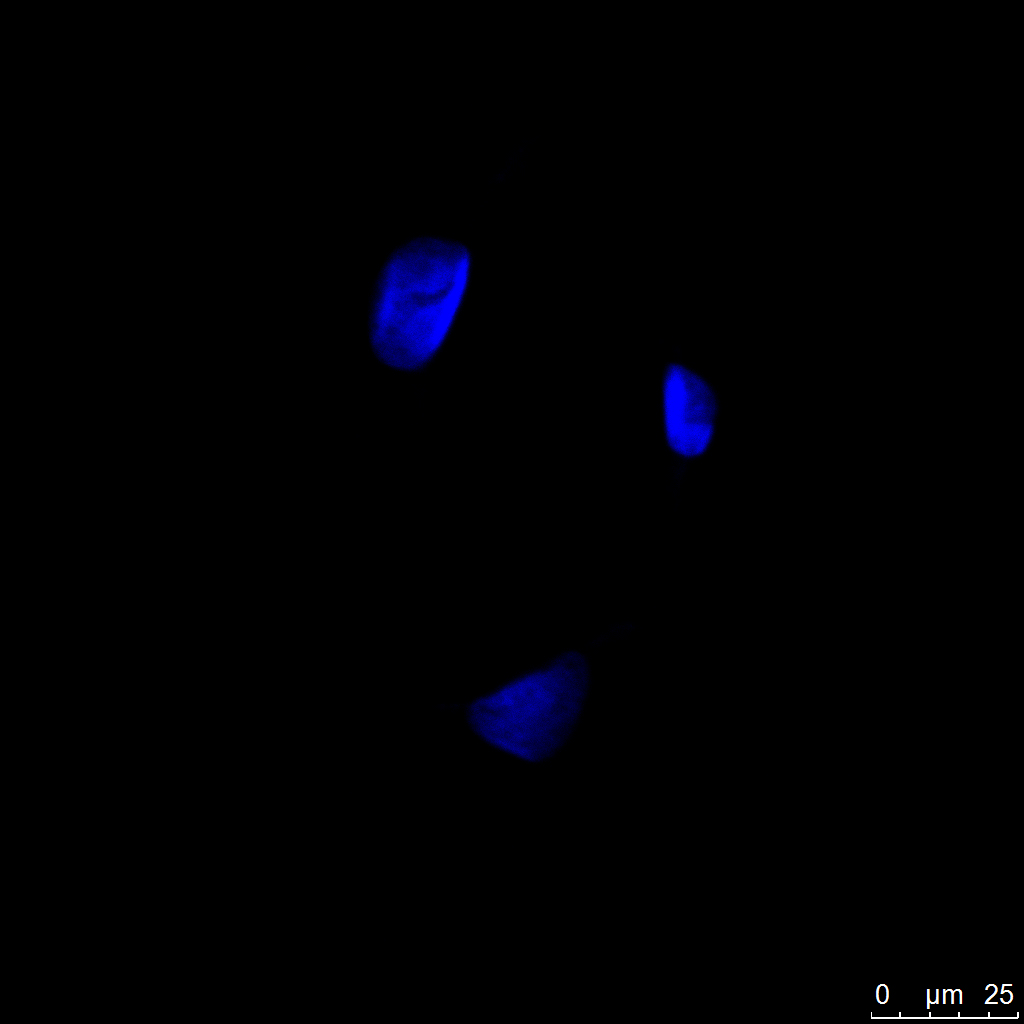

Supplement: Figure S2 — To further investigate interaction networks involving ELMO2 and Gαi2, immunofluorescence microscopy was used to examine the subcellular localization of the two proteins. [file peerj-08-8910-s002.zip › Immunofluorescence/20190715_Series029_z0_ch00.jpg]

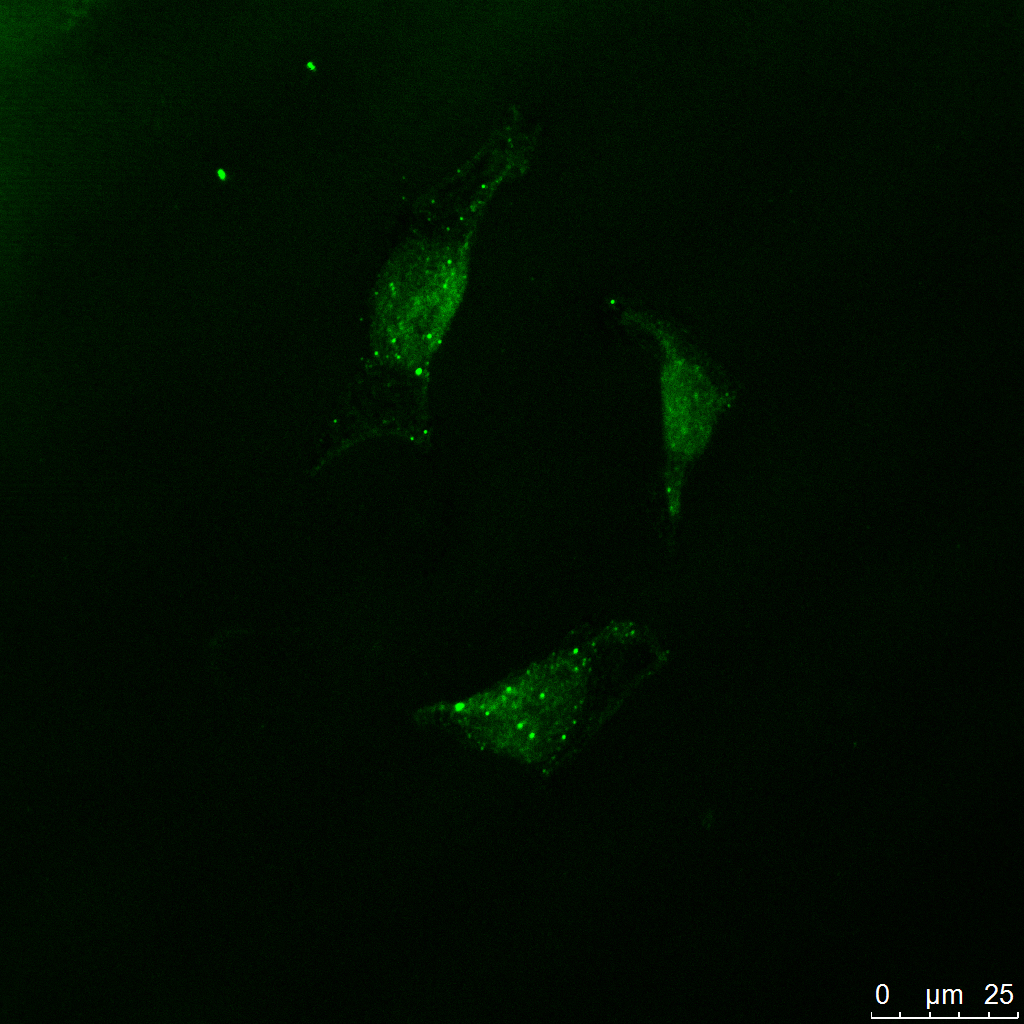

Supplement: Figure S2 — To further investigate interaction networks involving ELMO2 and Gαi2, immunofluorescence microscopy was used to examine the subcellular localization of the two proteins. [file peerj-08-8910-s002.zip › Immunofluorescence/20190715_Series029_z0_ch01.jpg]

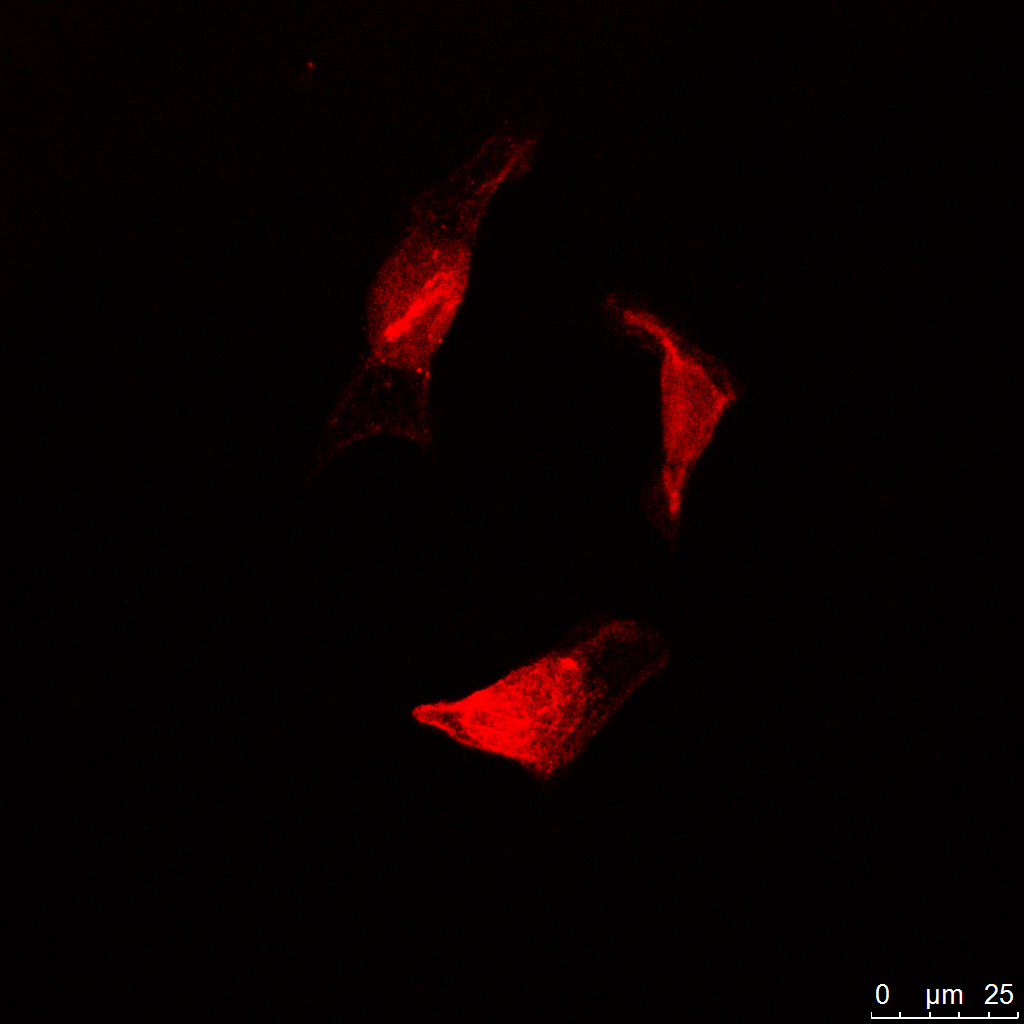

Supplement: Figure S2 — To further investigate interaction networks involving ELMO2 and Gαi2, immunofluorescence microscopy was used to examine the subcellular localization of the two proteins. [file peerj-08-8910-s002.zip › Immunofluorescence/20190715_Series029_z0_ch02.jpg]

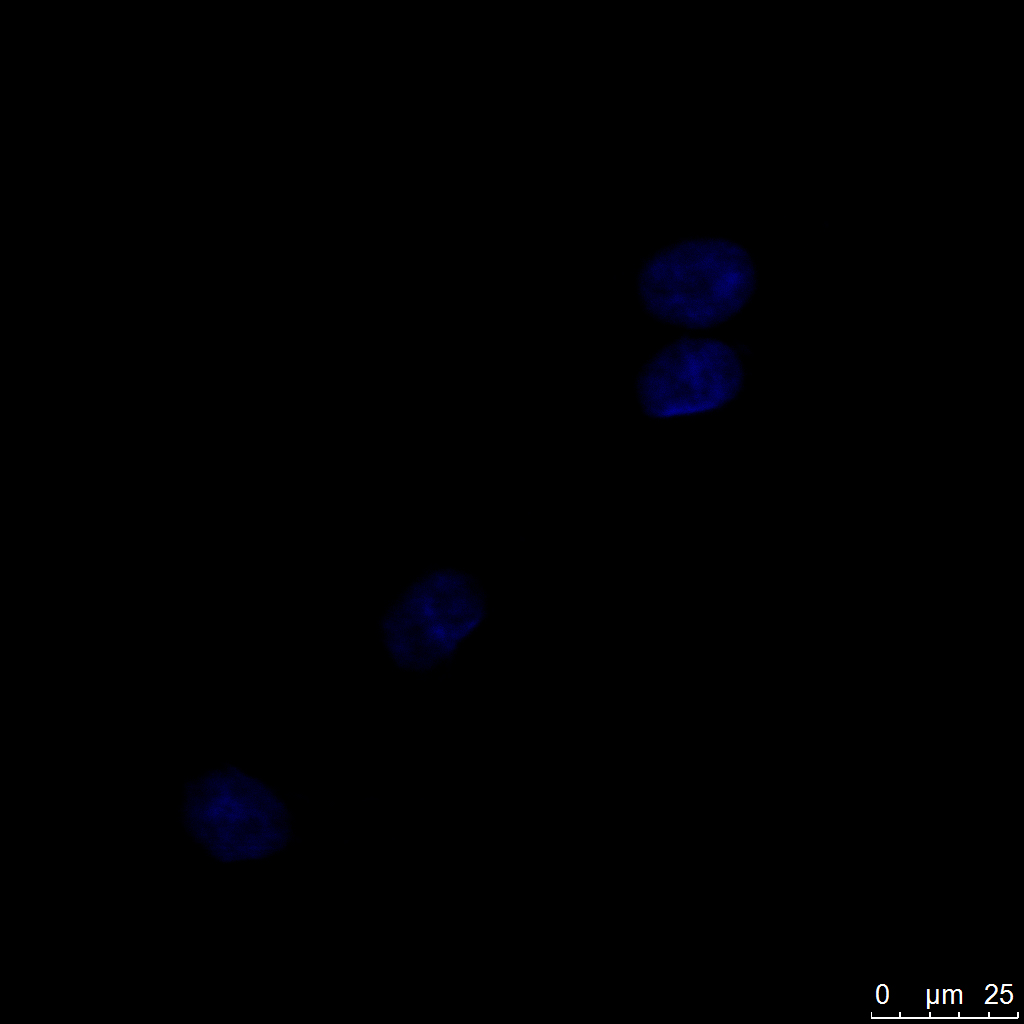

Supplement: Figure S2 — To further investigate interaction networks involving ELMO2 and Gαi2, immunofluorescence microscopy was used to examine the subcellular localization of the two proteins. [file peerj-08-8910-s002.zip › Immunofluorescence/20190715_Series043_z0_ch00.jpg]

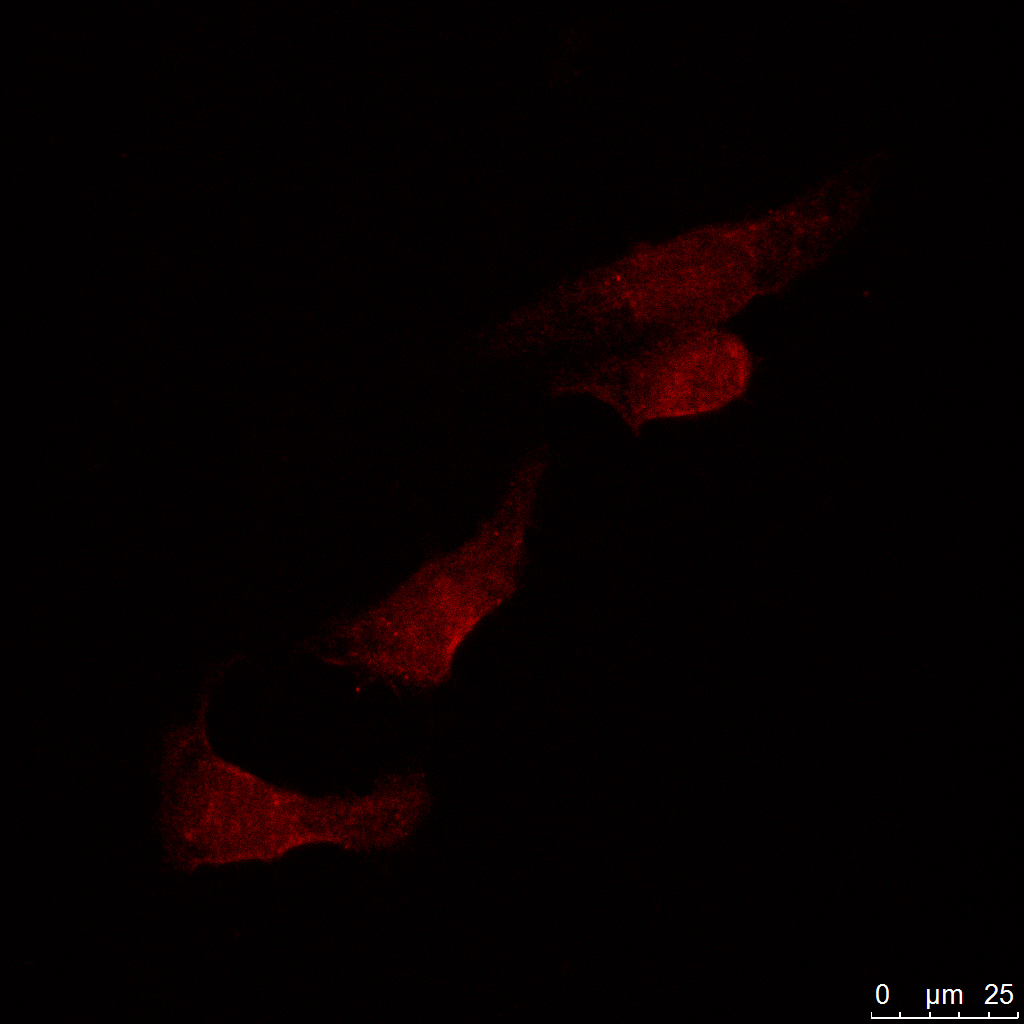

Supplement: Figure S2 — To further investigate interaction networks involving ELMO2 and Gαi2, immunofluorescence microscopy was used to examine the subcellular localization of the two proteins. [file peerj-08-8910-s002.zip › Immunofluorescence/20190715_Series043_z0_ch02.jpg]

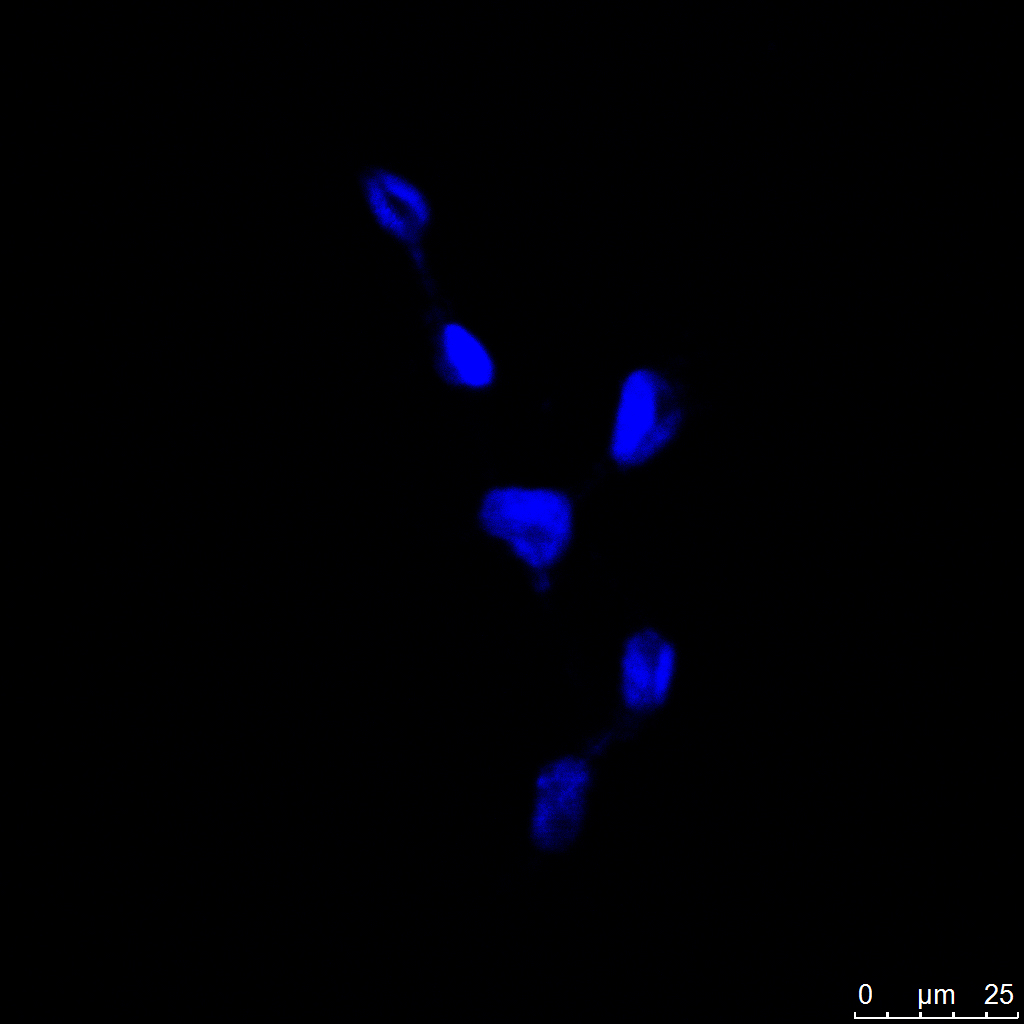

Supplement: Figure S2 — To further investigate interaction networks involving ELMO2 and Gαi2, immunofluorescence microscopy was used to examine the subcellular localization of the two proteins. [file peerj-08-8910-s002.zip › Immunofluorescence/20190722_Series020_z0_ch00.jpg]

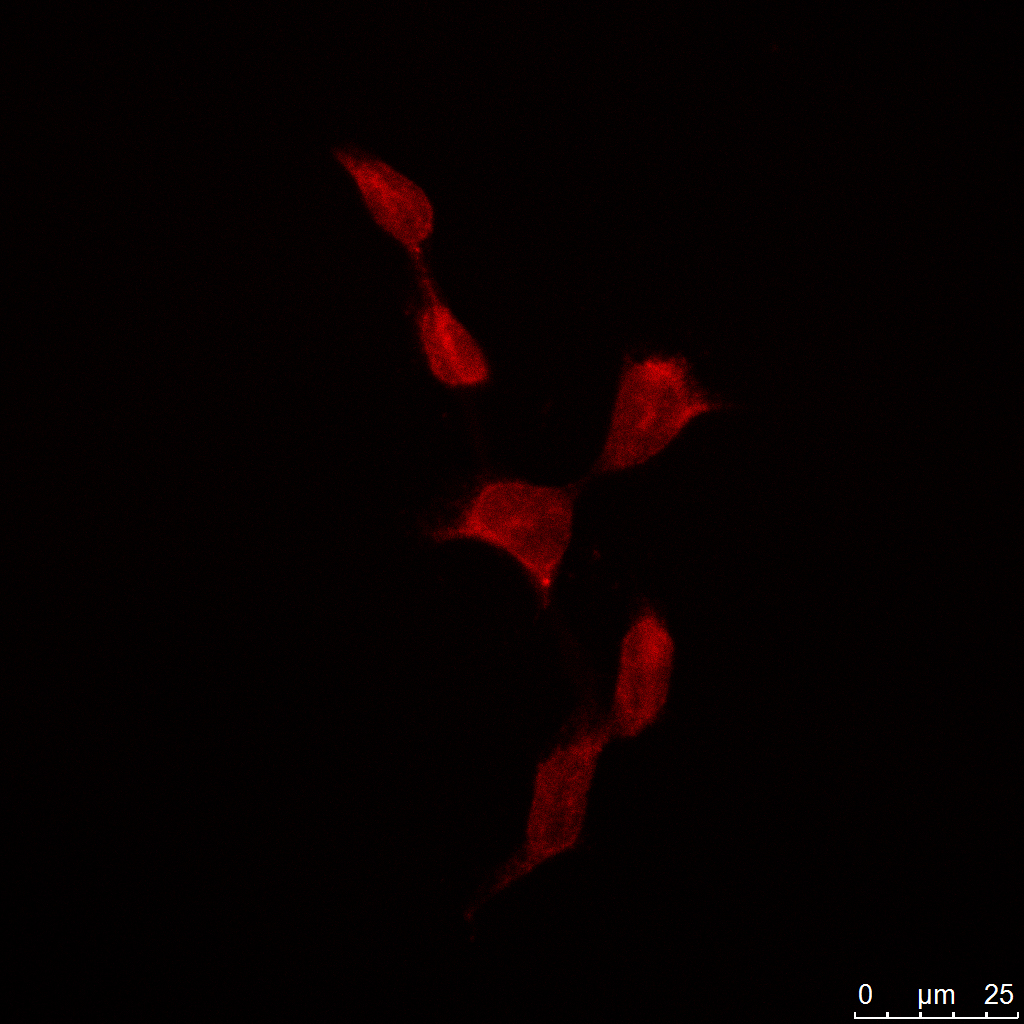

Supplement: Figure S2 — To further investigate interaction networks involving ELMO2 and Gαi2, immunofluorescence microscopy was used to examine the subcellular localization of the two proteins. [file peerj-08-8910-s002.zip › Immunofluorescence/20190722_Series020_z0_ch02.jpg]

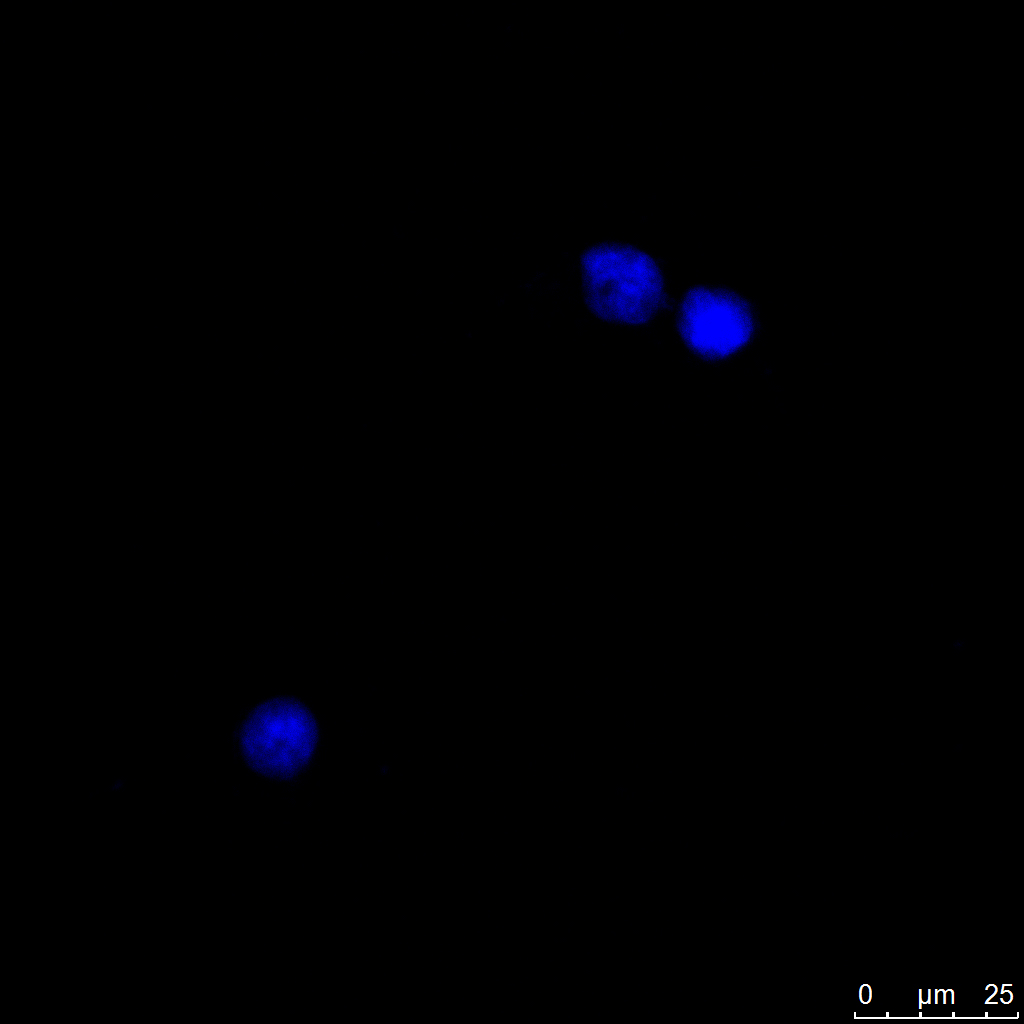

Supplement: Figure S2 — To further investigate interaction networks involving ELMO2 and Gαi2, immunofluorescence microscopy was used to examine the subcellular localization of the two proteins. [file peerj-08-8910-s002.zip › Immunofluorescence/20190722_Series028_z0_ch00.jpg]

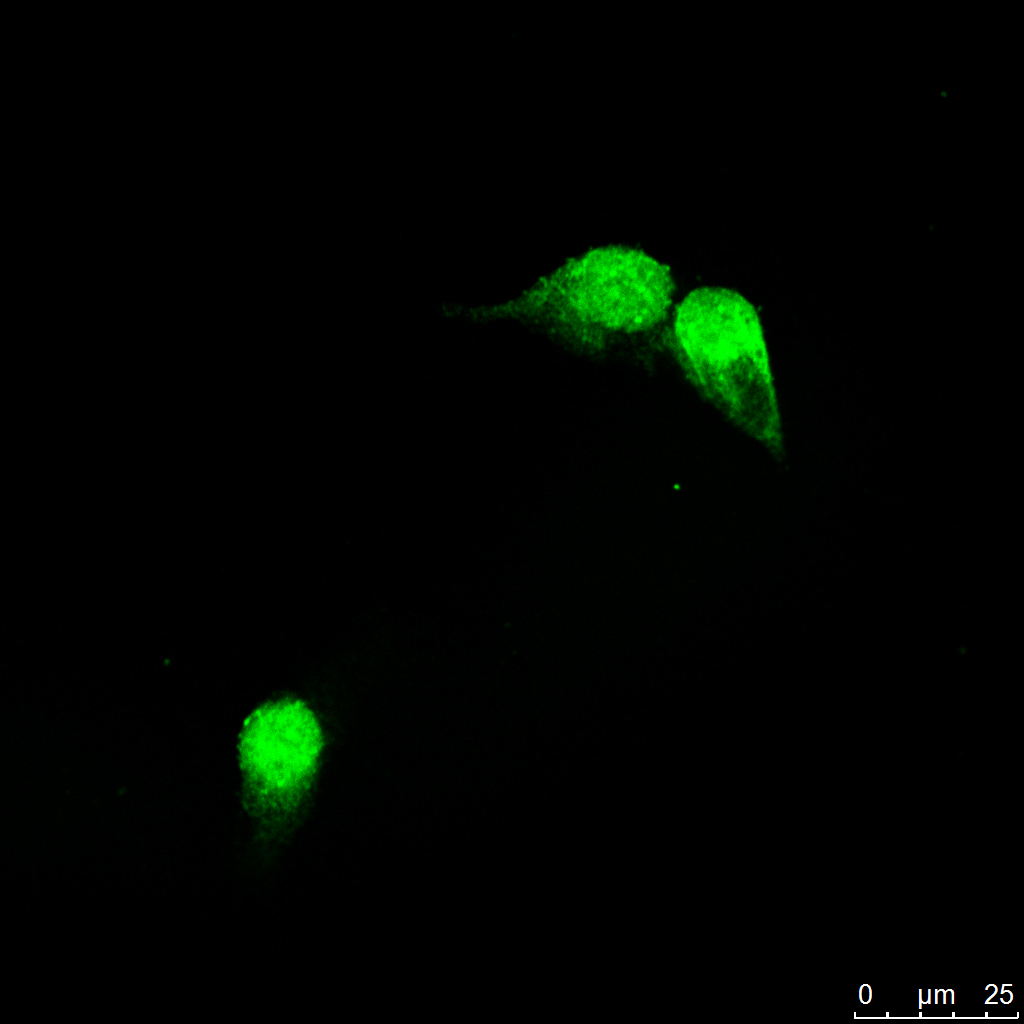

Supplement: Figure S2 — To further investigate interaction networks involving ELMO2 and Gαi2, immunofluorescence microscopy was used to examine the subcellular localization of the two proteins. [file peerj-08-8910-s002.zip › Immunofluorescence/20190722_Series028_z0_ch01.jpg]

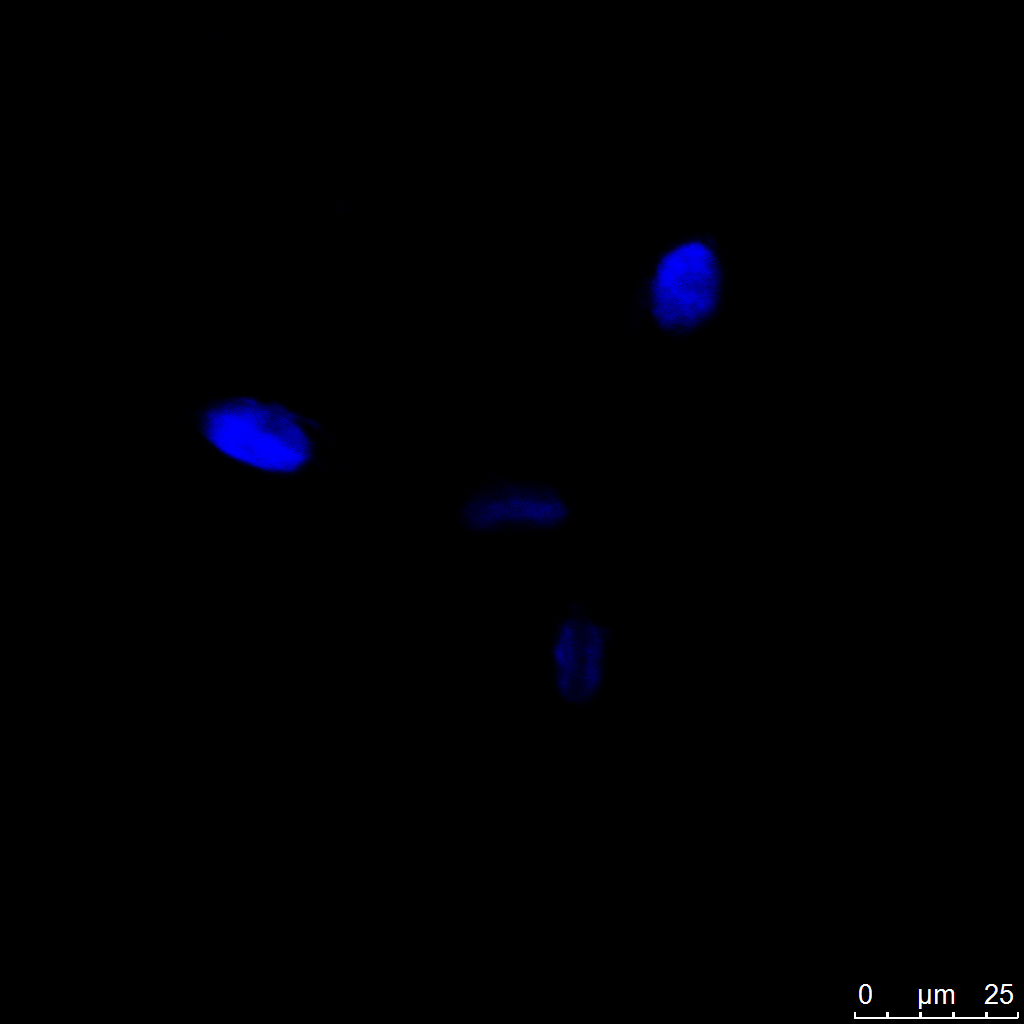

Supplement: Figure S2 — To further investigate interaction networks involving ELMO2 and Gαi2, immunofluorescence microscopy was used to examine the subcellular localization of the two proteins. [file peerj-08-8910-s002.zip › Immunofluorescence/20190722_Series040_z0_ch00.jpg]

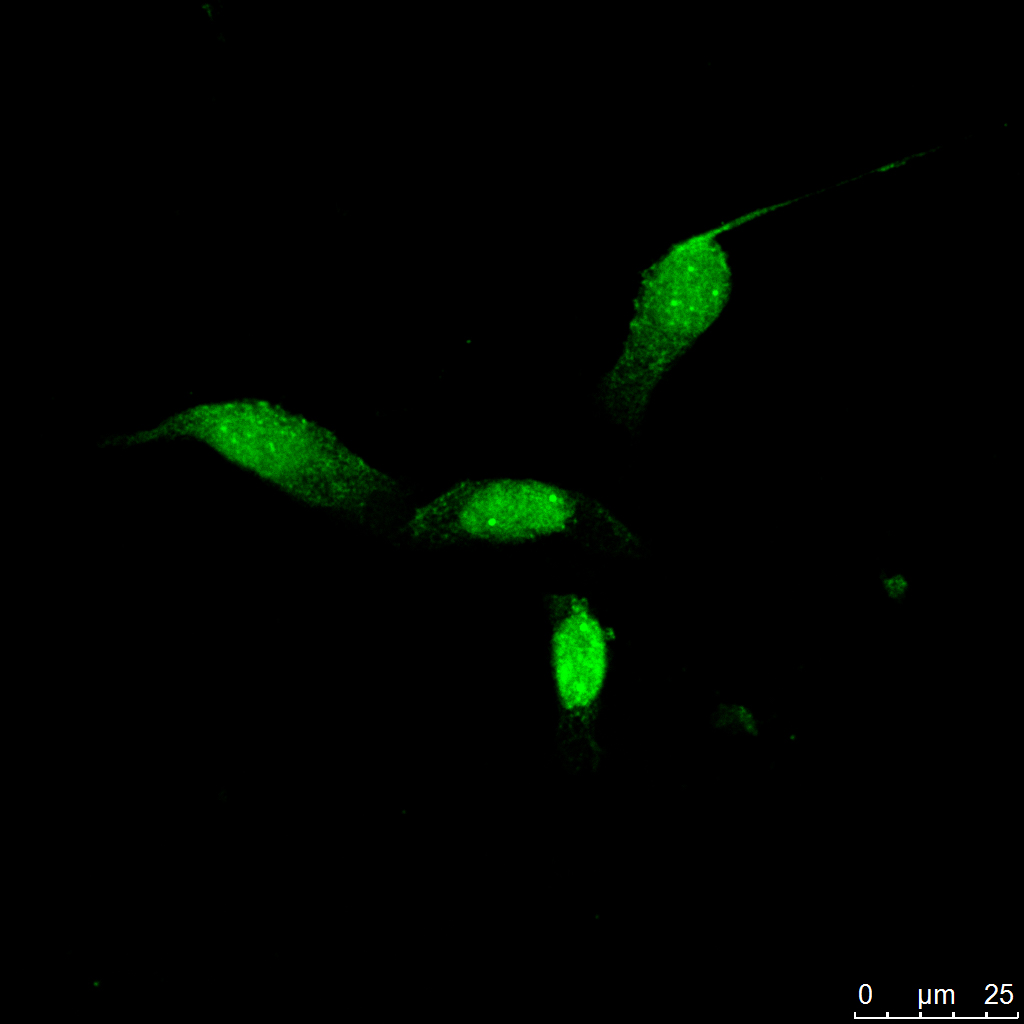

Supplement: Figure S2 — To further investigate interaction networks involving ELMO2 and Gαi2, immunofluorescence microscopy was used to examine the subcellular localization of the two proteins. [file peerj-08-8910-s002.zip › Immunofluorescence/20190722_Series040_z0_ch01.jpg]

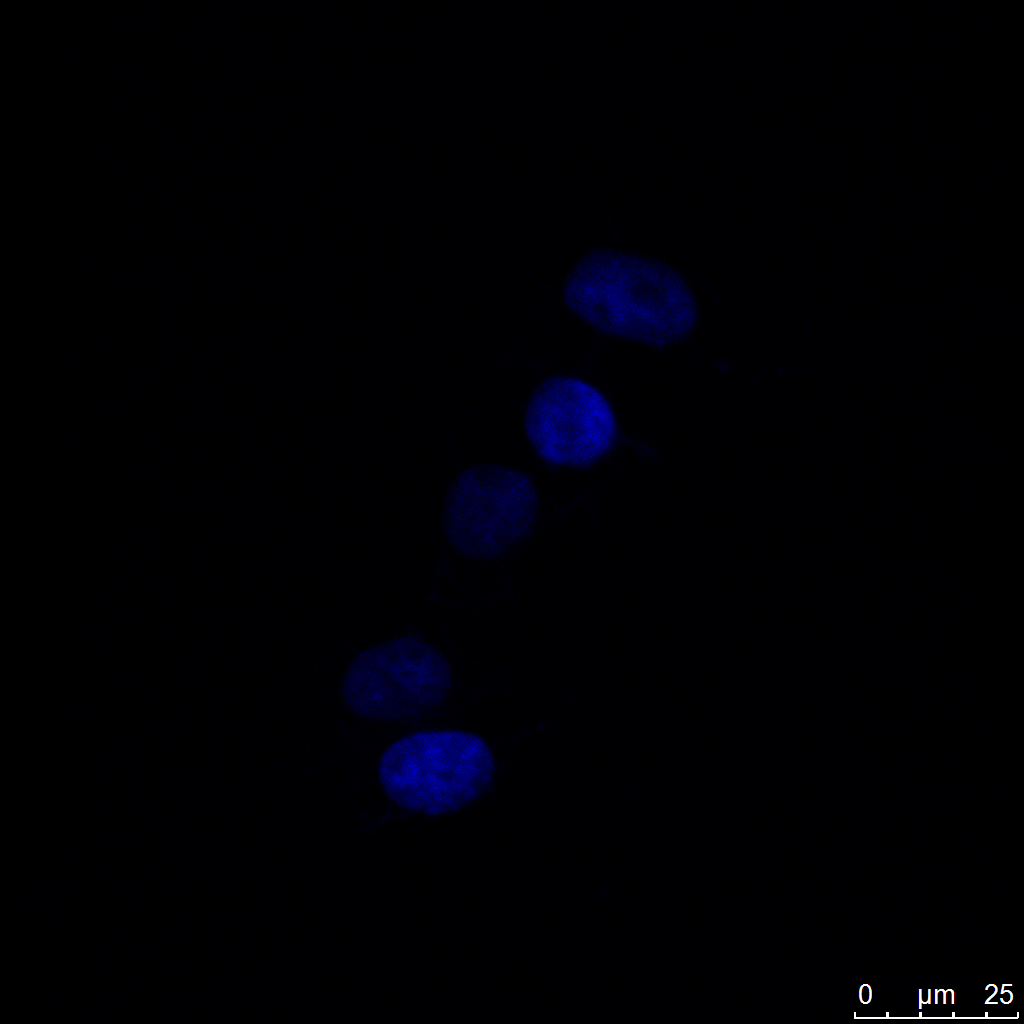

Supplement: Figure S2 — To further investigate interaction networks involving ELMO2 and Gαi2, immunofluorescence microscopy was used to examine the subcellular localization of the two proteins. [file peerj-08-8910-s002.zip › Immunofluorescence/20190722_Series059_z0_ch00.jpg]

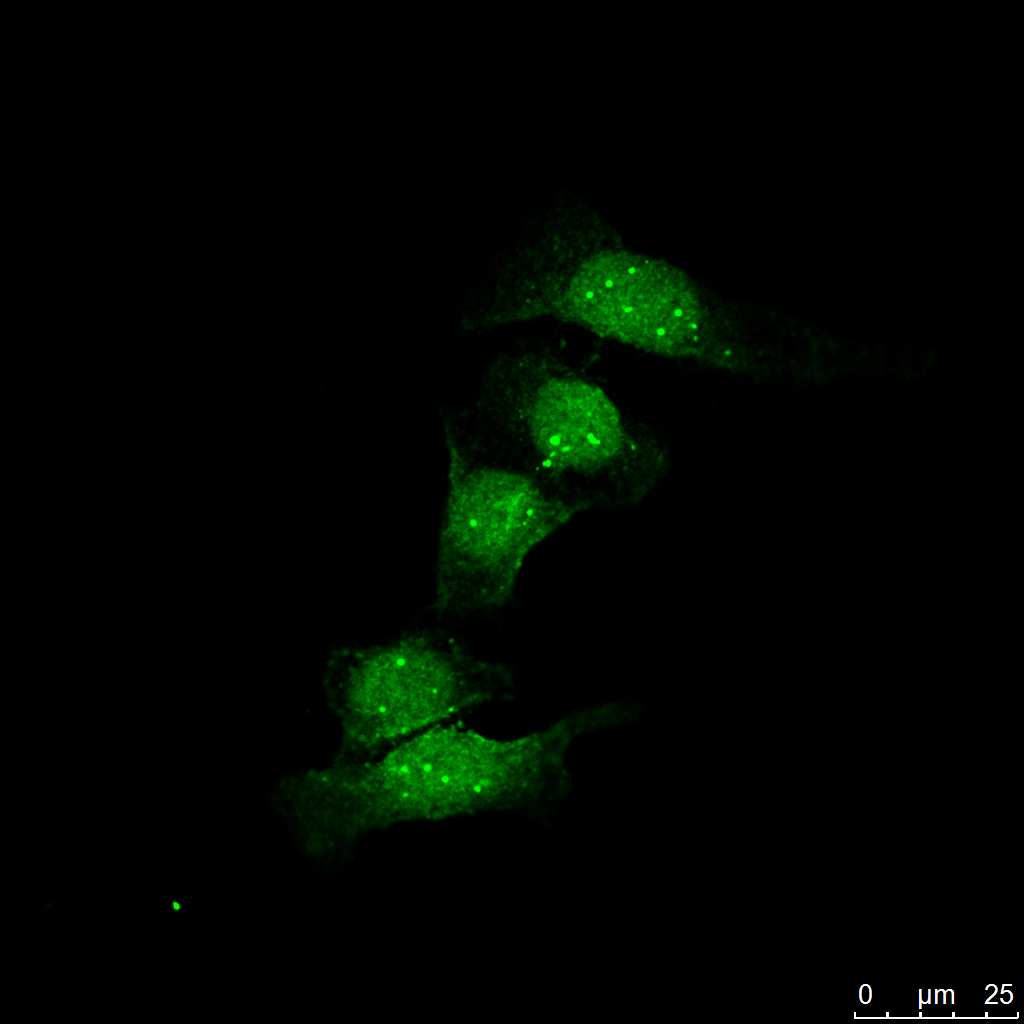

Supplement: Figure S2 — To further investigate interaction networks involving ELMO2 and Gαi2, immunofluorescence microscopy was used to examine the subcellular localization of the two proteins. [file peerj-08-8910-s002.zip › Immunofluorescence/20190722_Series059_z0_ch01.jpg]

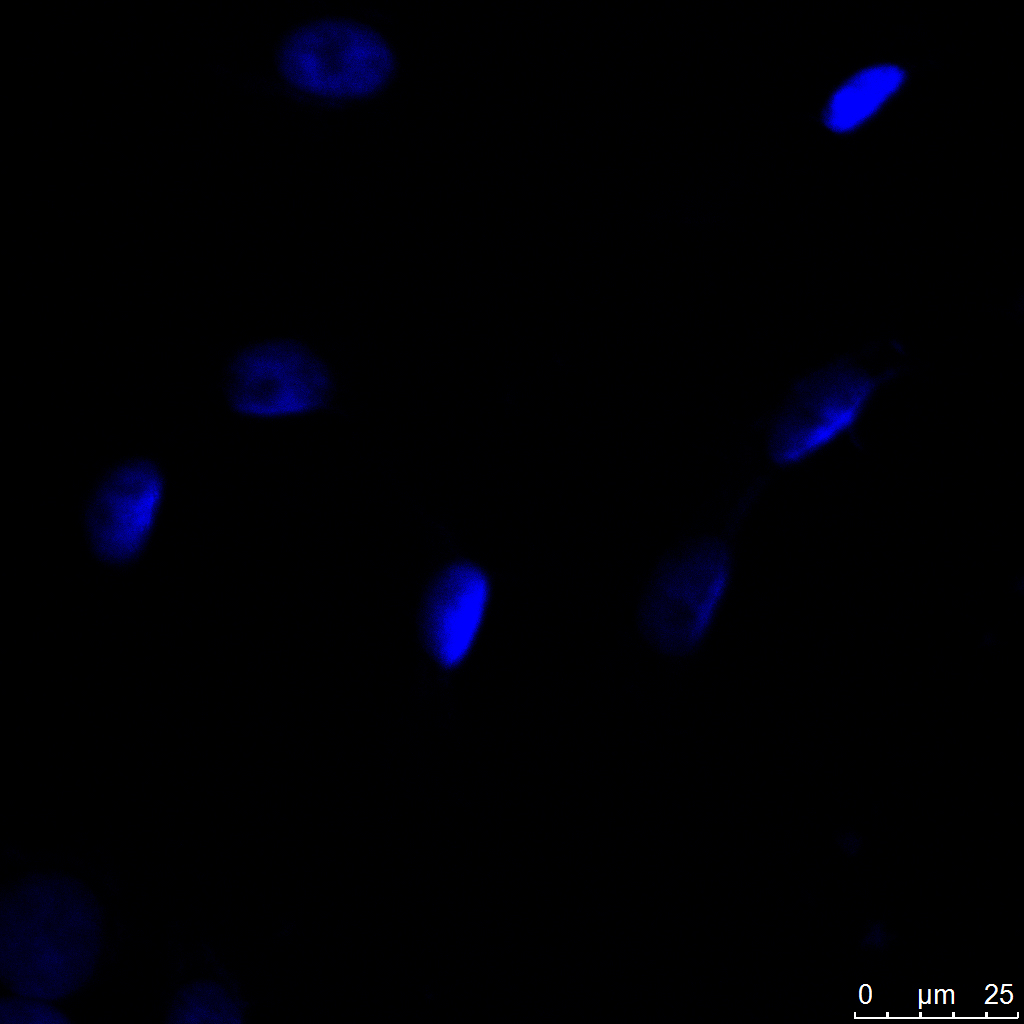

Supplement: Figure S2 — To further investigate interaction networks involving ELMO2 and Gαi2, immunofluorescence microscopy was used to examine the subcellular localization of the two proteins. [file peerj-08-8910-s002.zip › Immunofluorescence/20190722_Series063_z0_ch00.jpg]

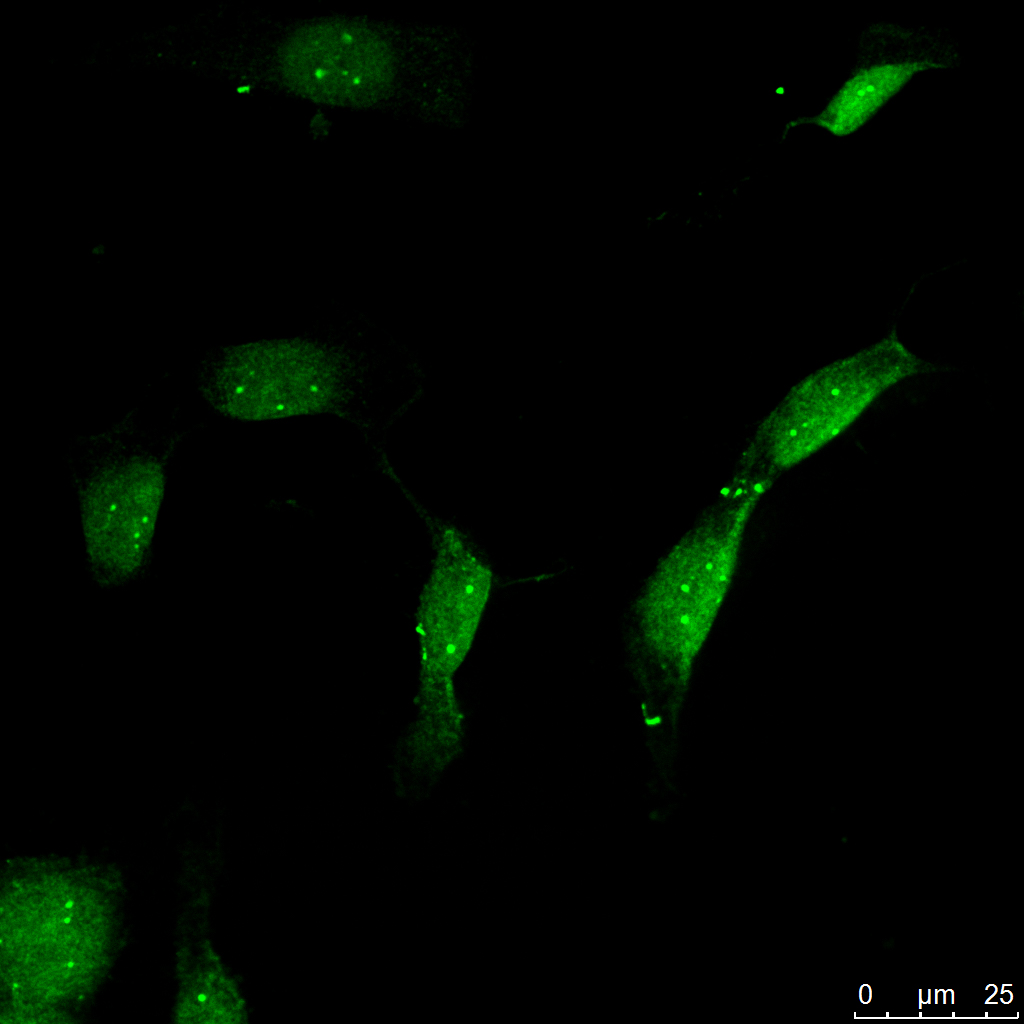

Supplement: Figure S2 — To further investigate interaction networks involving ELMO2 and Gαi2, immunofluorescence microscopy was used to examine the subcellular localization of the two proteins. [file peerj-08-8910-s002.zip › Immunofluorescence/20190722_Series063_z0_ch01.jpg]

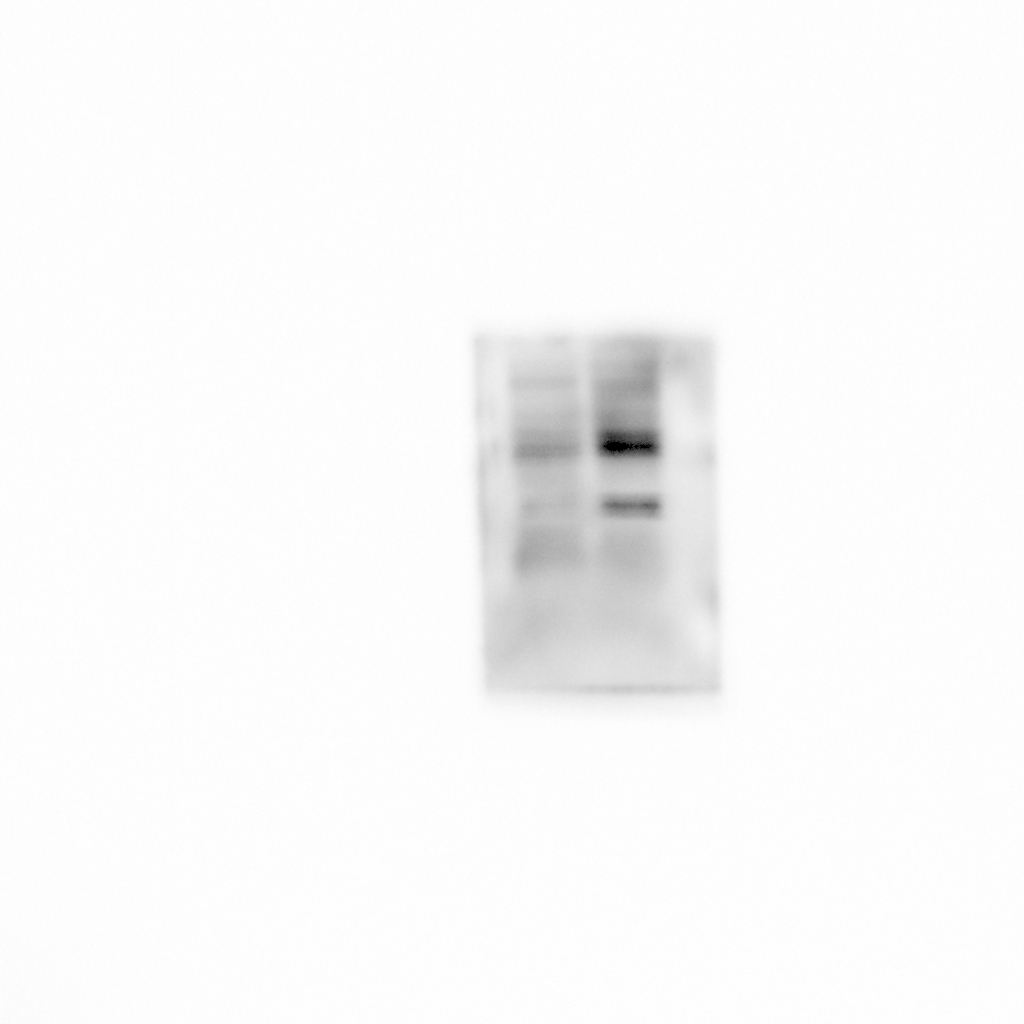

Supplement: Figure S3 — Our results confirmed the physical association between ELMO2 and Gαi2 in pancreatic cancer cells. [file peerj-08-8910-s003.zip › WesternBlotting/Co-ip/OverEx _GNAI2/iB_elmo2.jpg]

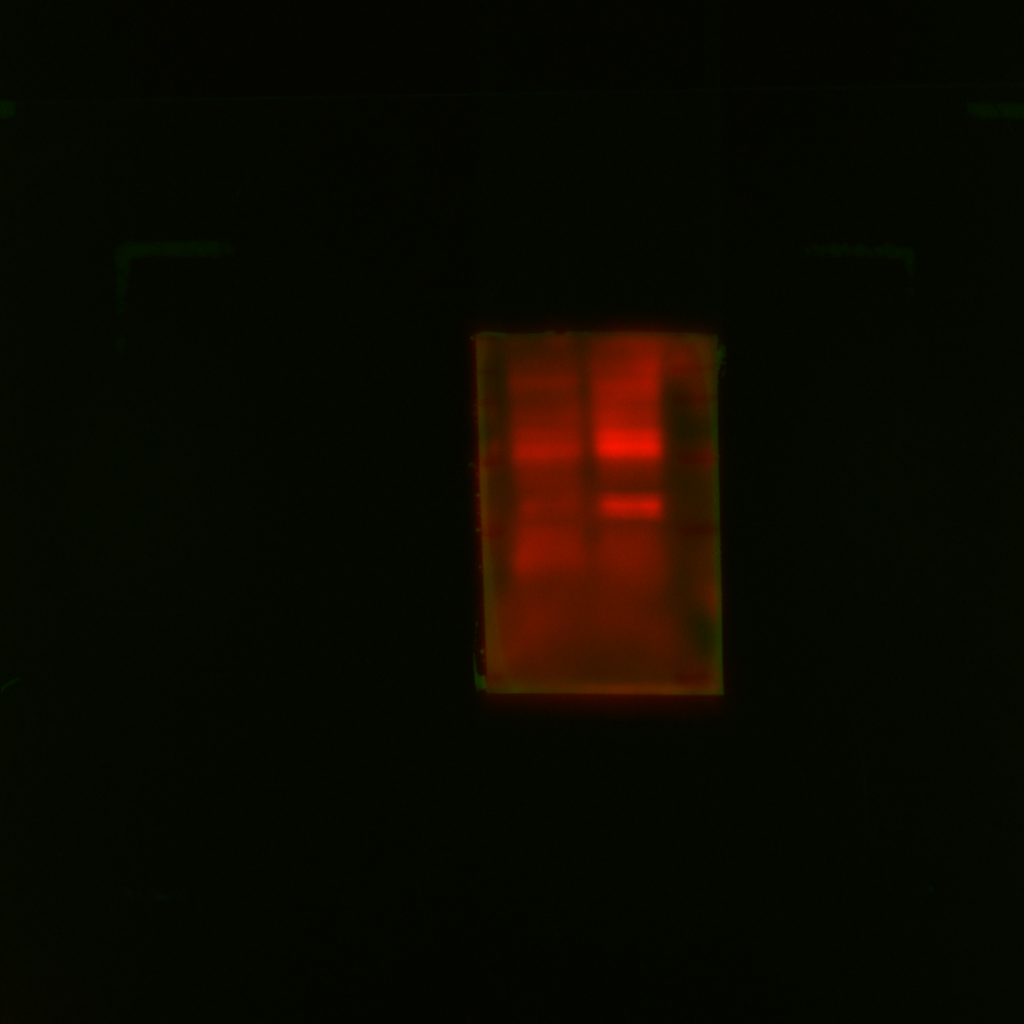

Supplement: Figure S3 — Our results confirmed the physical association between ELMO2 and Gαi2 in pancreatic cancer cells. [file peerj-08-8910-s003.zip › WesternBlotting/Co-ip/OverEx _GNAI2/merge_iB_elmo2.jpg]

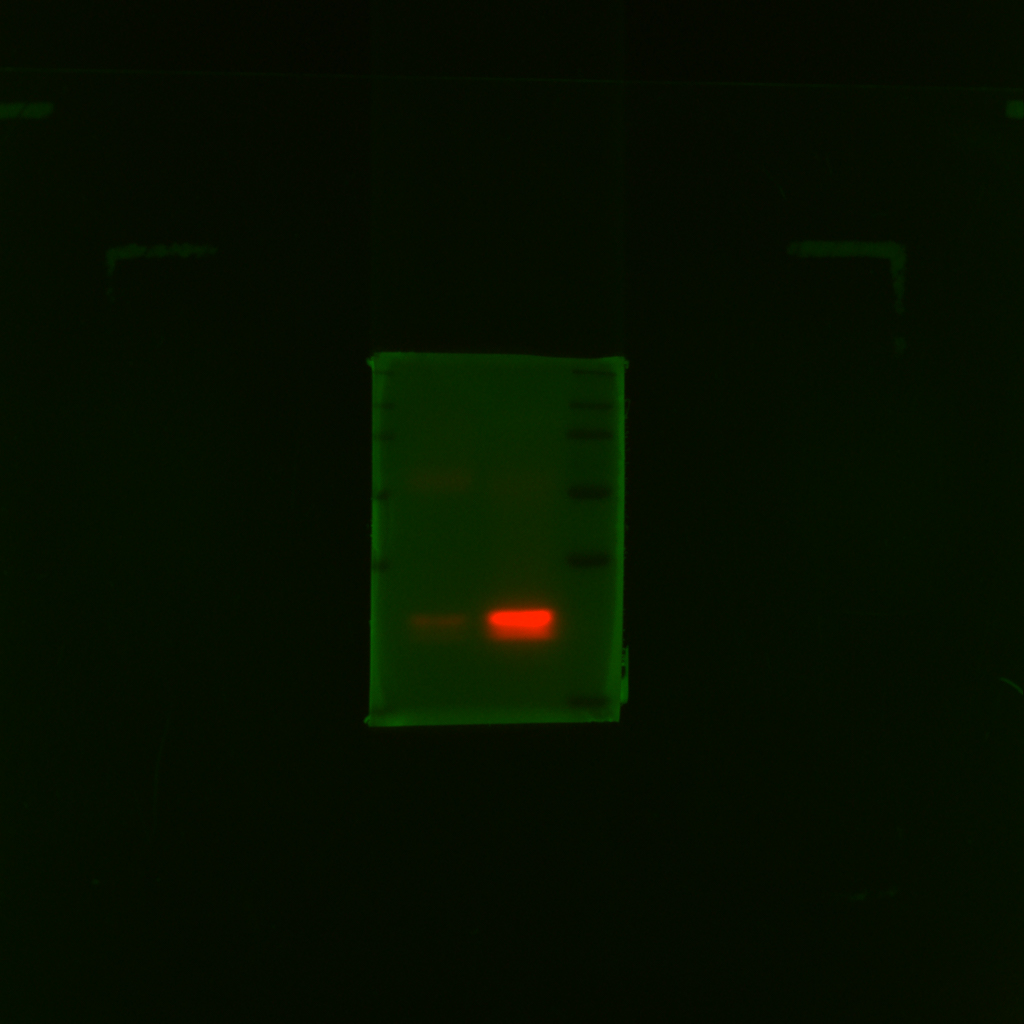

Supplement: Figure S3 — Our results confirmed the physical association between ELMO2 and Gαi2 in pancreatic cancer cells. [file peerj-08-8910-s003.zip › WesternBlotting/Co-ip/OverEx _GNAI2/merge_overEx_GNAI2.jpg]

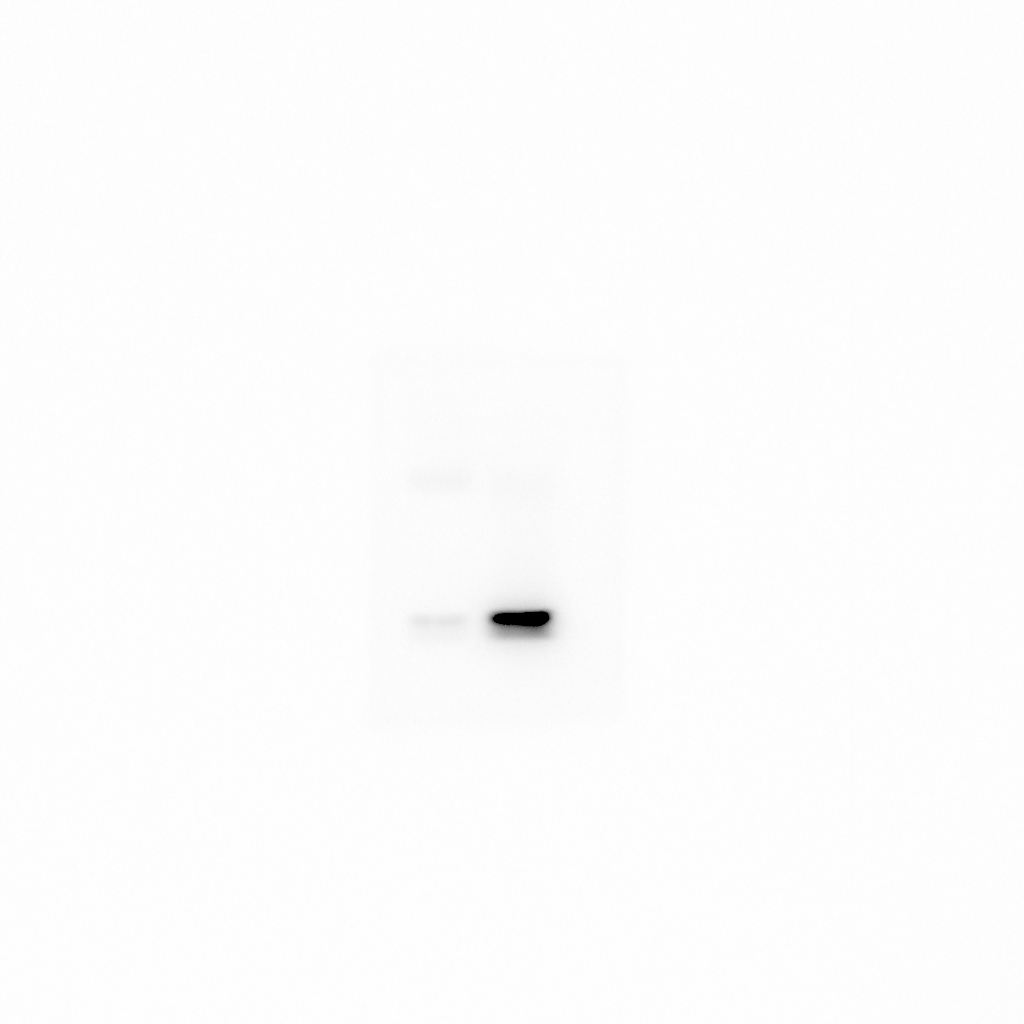

Supplement: Figure S3 — Our results confirmed the physical association between ELMO2 and Gαi2 in pancreatic cancer cells. [file peerj-08-8910-s003.zip › WesternBlotting/Co-ip/OverEx _GNAI2/overEx_GNAI2.jpg]

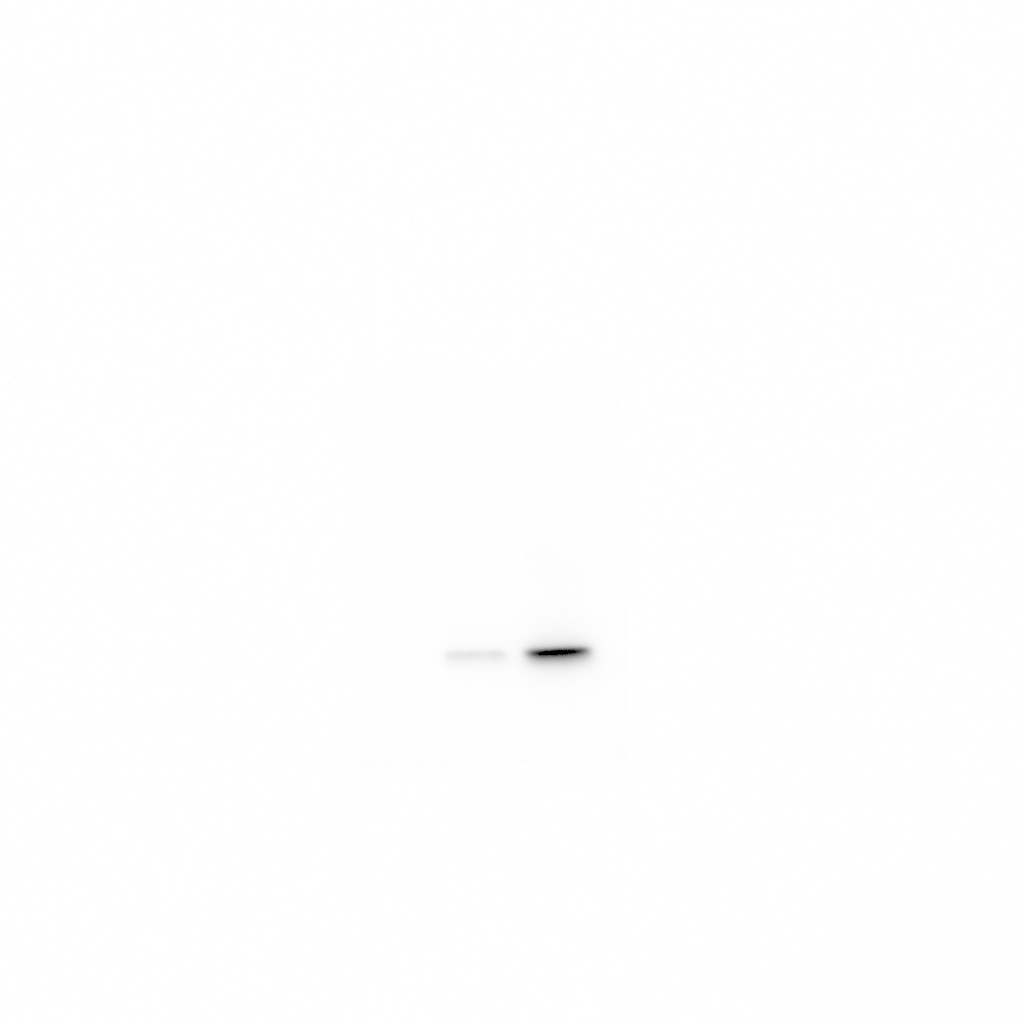

Supplement: Figure S3 — Our results confirmed the physical association between ELMO2 and Gαi2 in pancreatic cancer cells. [file peerj-08-8910-s003.zip › WesternBlotting/Co-ip/OverEx_elmo2/iB_GNAI2.jpg]

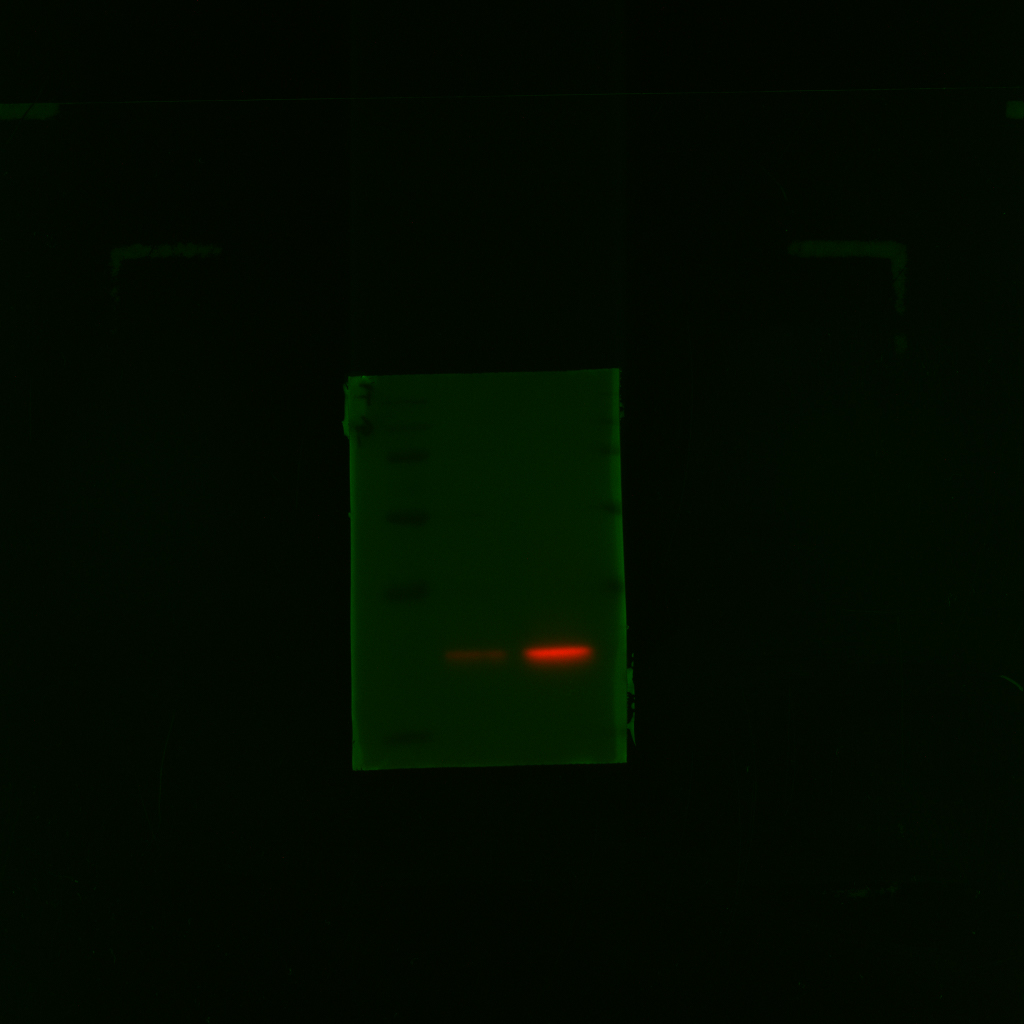

Supplement: Figure S3 — Our results confirmed the physical association between ELMO2 and Gαi2 in pancreatic cancer cells. [file peerj-08-8910-s003.zip › WesternBlotting/Co-ip/OverEx_elmo2/merge_iB_GNAI2.jpg]

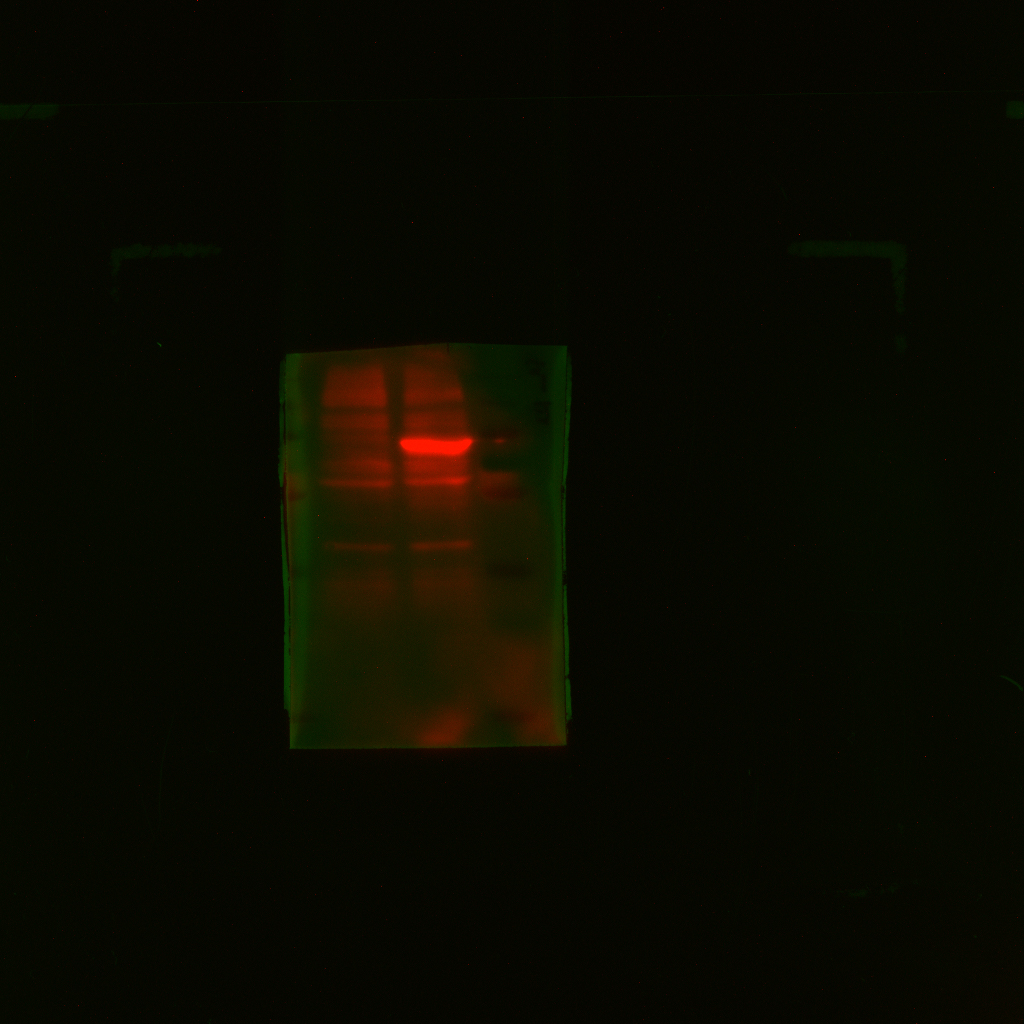

Supplement: Figure S3 — Our results confirmed the physical association between ELMO2 and Gαi2 in pancreatic cancer cells. [file peerj-08-8910-s003.zip › WesternBlotting/Co-ip/OverEx_elmo2/merge_overEx_ELMO2.jpg]

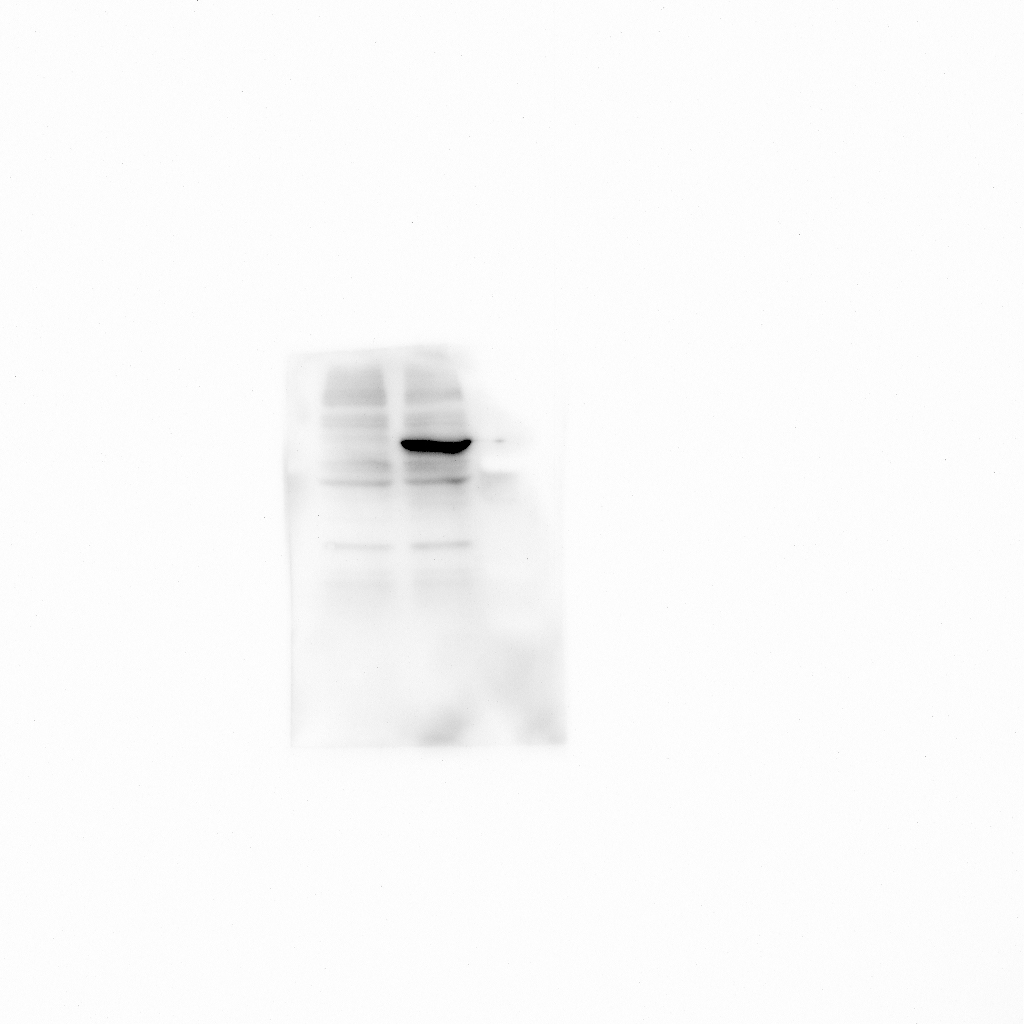

Supplement: Figure S3 — Our results confirmed the physical association between ELMO2 and Gαi2 in pancreatic cancer cells. [file peerj-08-8910-s003.zip › WesternBlotting/Co-ip/OverEx_elmo2/overEx_ELMO2.jpg]
