# Supplementary figures and images for: ELMO2 association with Gαi2 regulates pancreatic cancer cell chemotaxis and metastasis (part 2 of 2)
Source: PeerJ. 2020 Apr 6;8:e8910. doi: 10.7717/peerj.8910 (PMC7144586; doi:10.7717/peerj.8910)

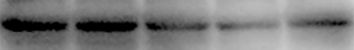

Supplement: Figure S3 — Our results confirmed the physical association between ELMO2 and Gαi2 in pancreatic cancer cells. [file peerj-08-8910-s003.zip › WesternBlotting/ELMO2_konckdown/Aspc-1---KD.jpg]

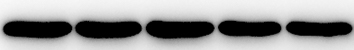

Supplement: Figure S3 — Our results confirmed the physical association between ELMO2 and Gαi2 in pancreatic cancer cells. [file peerj-08-8910-s003.zip › WesternBlotting/ELMO2_konckdown/Aspc-1--GAPDH.jpg]

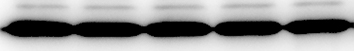

Supplement: Figure S3 — Our results confirmed the physical association between ELMO2 and Gαi2 in pancreatic cancer cells. [file peerj-08-8910-s003.zip › WesternBlotting/ELMO2_konckdown/panc-1--GAPDH.jpg]

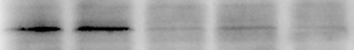

Supplement: Figure S3 — Our results confirmed the physical association between ELMO2 and Gαi2 in pancreatic cancer cells. [file peerj-08-8910-s003.zip › WesternBlotting/ELMO2_konckdown/panc-1--kd.jpg]

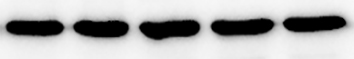

Supplement: Figure S3 — Our results confirmed the physical association between ELMO2 and Gαi2 in pancreatic cancer cells. [file peerj-08-8910-s003.zip › WesternBlotting/GNAI2_konckdown/KD--GNAI2--GAPDH--panc-1.jpg]

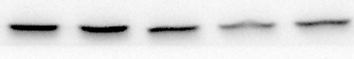

Supplement: Figure S3 — Our results confirmed the physical association between ELMO2 and Gαi2 in pancreatic cancer cells. [file peerj-08-8910-s003.zip › WesternBlotting/GNAI2_konckdown/KD--GNAI2--panc-1.jpg]
